# FAK- and TGF-β1-dependent biomechanical alterations in microvascular endothelial cells exposed to fluid shear stress gradients

**DOI:** 10.1038/s42003-026-11283-9

**Published:** 2026-11-03

**Authors:** Julio César Sánchez-Rendón, Erva Keskin, Nadine Oder, Annalena Reuss, Eleftheria Michalaki, Gerald G. Fuller, Alexander Dunn, Effie E. Bastounis

**Affiliations:** 1 Institute for Biology, Humboldt-Universität zu Berlin, Berlin, Germany; 2 Interfaculty Institute of Microbiology and Infection Medicine, Cluster of Excellence “Controlling Microbes to Fight Infections” (CMFI, EXC 2124), Universität Tübingen, Tübingen, Germany; 3 Department of Chemical Engineering, Stanford University School of Engineering, Stanford, CA USA

**Keywords:** Mechanotransduction, Collective cell migration

## Abstract

Endothelial cells (EC) form a dynamic monolayer barrier that regulates vascular permeability and is highly sensitive to mechanical cues, including shear stresses (SS) generated by blood flow. While spatial shear stress gradients (SSG) are linked to vascular pathologies such as atherosclerosis, their direct effects on cellular biomechanics across EC subtypes remain underexplored. Here, using an impinging flow device compatible with videomicroscopy, we demonstrate that exposure of two EC types to SSG causes an immediate and sustained two-fold decrease in migration speed, accompanied by enhanced coordination of collective motion and directional migration against the flow. Concurrently, EC traction and monolayer stresses increase acutely upon flow onset and continue to rise linearly over time. Mechanistically, FAK-mediated mechanotransduction governs flow-opposed migration, whereas the time-dependent increase in traction depends on TGF-β1 signaling and actomyosin contractility. Together, these findings identify SSG as key regulators of EC mechanics, collective dynamics, and signaling, with important implications for vascular disease diagnostics and therapeutics.

## Abstract (web summary)

Using an impinging flow device, we investigate how spatial shear stress gradients shape microvascular endothelial cell mechanics and collective migration. In response to flow endothelial cells slow down and migrate against the flow, while enhancing their traction and monolayer stresses through distinct FAK- and TGF-β1-dependent mechanisms.

## Introduction

Endothelial cells (EC) form a single monolayer lining the inner lumen of blood vessels and serve as a selective barrier that regulates molecular transport while limiting the spread of pathogens and other insults. To fulfill these functions, EC continuously sense and respond to mechanical cues from blood flow, including shear stresses (SS), which regulate major endothelial functions including angiogenesis, vessel remodeling, and even disease progression^1–4^. Unlike macrovascular EC, which experience relatively uniform flow with nearly constant magnitude SS, microvascular endothelial cells (μEC) in capillary networks are exposed to highly heterogeneous flow environments characterized by steep spatial shear stress gradients (SSG). These gradients naturally arise from vessel bifurcations or curvature, but can also be caused by pathological conditions such as atherosclerosis^5–7^. Despite their (patho)physiological relevance, most studies on endothelial mechanobiology have focused on macrovascular EC exposed to uniform SS, leaving μEC responses to SSG comparatively underexplored^3, 8–10^.

To address this gap, an impinging flow device compatible with long-term live-cell microscopy was developed and mounted on multi-well glass-bottom plates, enabling the simultaneous monitoring of multiple conditions^9, 11^. This platform was used to study the kinematic response of EC to SSG, and it was found that lymphatic μEC exhibit density-dependent rheotactic behavior; that is, at high confluency, they migrate against the flow (reverse rheotaxis) and accumulate in regions of high SS, whereas at lower density, they migrate with the flow (forward rheotaxis)^11^. Additionally, lymphatic μEC align parallel to flow at low SS but reorient perpendicularly above a critical threshold^11^. Reverse rheotaxis in response to SSG is specific to lymphatic μEC, as macrovascular EC and other cell types migrate with the flow^9^. Constant SS can also stimulate rheotaxis of varying direction depending on the EC origin, with arterial EC exhibiting forward rheotaxis, venous EC reverse rheotaxis, and μEC a more complex migratory behavior possibly due to arising gradients^12^.

EC detect SS through a range of surface receptors and mechanosensitive complexes^13–15^. Studies in μΕC identified the S1P-S1PR1 axis and shear-correlated calcium signaling as molecular regulators of their response to flow-induced SSG^10^. While additional signaling components may also be implicated, it is unknown how cell-generated forces through alterations in cytoskeletal and focal adhesion dynamics contribute to rheotaxis, and whether similar force-dependent mechanisms operate in other μEC subtypes^16, 17^. Given the functional importance of μEC in processes such as wound healing, angiogenesis, and barrier function, deciphering the mechanosensitive migration patterns of these cells in response to flow is crucial.

Some insights have come from studies on human umbilical vein EC (HUVEC). Confluent HUVECs increase the traction stresses they exert on their matrices and their alignment during prolonged ( ~ 24 h) exposure to laminar but not oscillatory shear^18^, as do human pulmonary artery EC^19^. Consistently, another study showed that exposure to constant SS increases HUVEC traction stresses relative to static conditions, with focal adhesion-mediated mechanotransduction being critical in driving cytoskeletal reorganization and polarization along the flow direction^20^. Contrary to these prior studies, another study reported that in HUVECs under laminar flow, both traction and monolayer stresses (inter- and intra-cellular stresses) promptly decrease in magnitude but align along the flow direction, which is followed by HUVEC elongation and alignment^21^. The discrepancies between these studies may stem from experimental differences in SS magnitude, time windows of observation, stiffness of the cells’ matrix or confluence. While providing mechanistic insight into biomechanical responses to uniform flow-induced SS, these studies are largely restricted to HUVEC and do not address EC responses to SSG.

In this study, we leveraged an impinging flow device to investigate how SSG influences μEC biomechanics. Collectively, our findings suggest that SSG critically regulates EC rheotaxis, with μEC migrating against the flow direction in a FAK-dependent manner by increasing their traction stresses in response to TGF-β1. These insights provide a mechanistic framework for understanding how EC adapt to complex hemodynamic environments, with crucial implications in advancing our understanding of vascular homeostasis and disease progression.

## Results

### Exposure of μEC to SSG reduces migration speed while enhancing collective motion

**Fig. 1:**
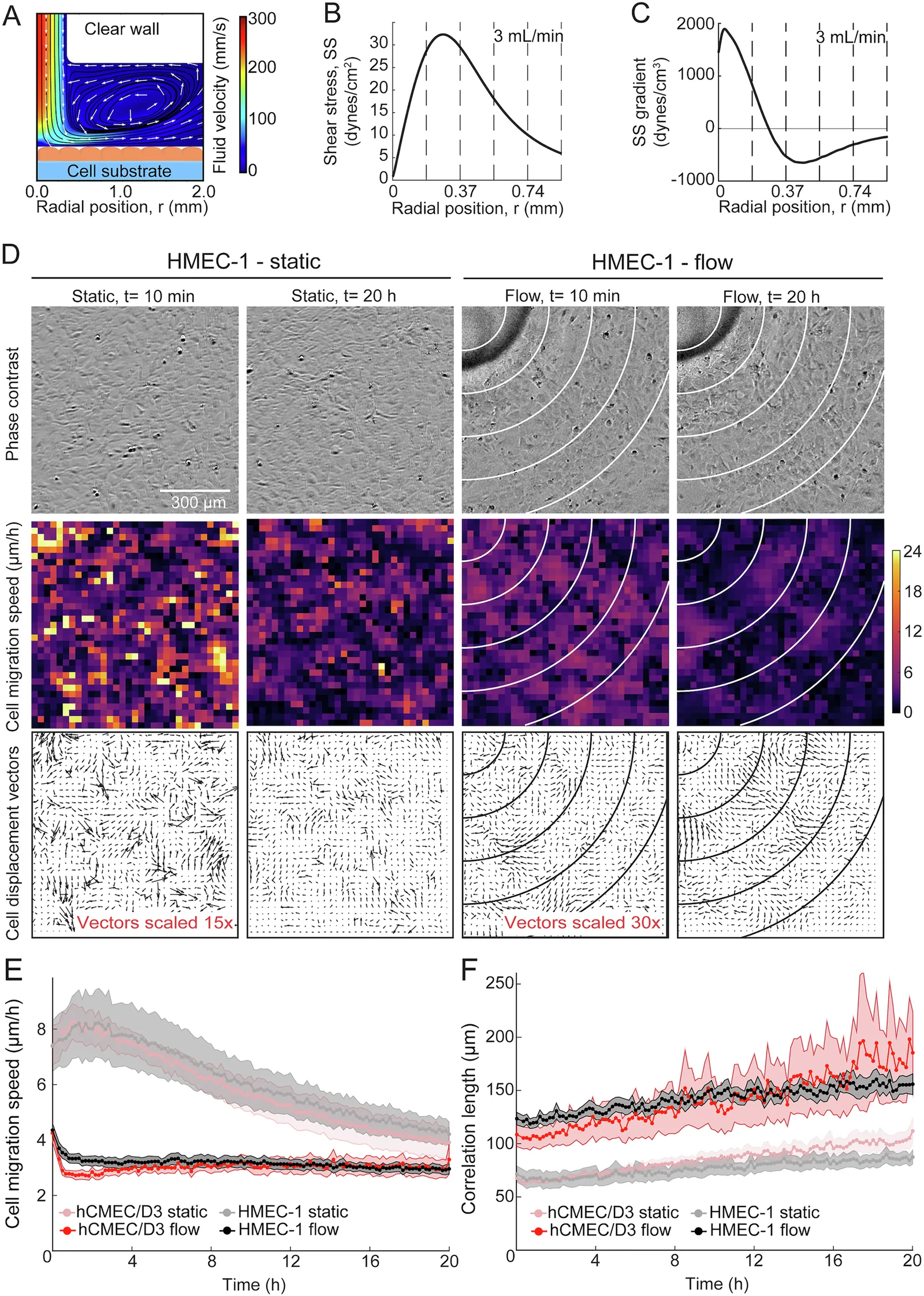
SSG slows μEC migration speed while enhancing motion coordination. **A** Finite element simulation of the cross-sectional flow velocity profile and fluid streamlines (white arrows) at 3 mL/min flow rate, illustrating the spatially varying axisymmetric flow experienced by cells using the impinging flow jet device. **B-C** Simulated SS (Β) and SSG (C) as a function of the radial distance from the stagnation point (*r* = 0 μm), which is located directly below the flow orifice center at a 3 mL/min flow rate. Vertical dashed lines indicate equally spaced radial positions from the jet center and correspond directly to the concentric contours overlaid on the microscopy images in D and throughout subsequent figures, thereby relating the local SS and SSG values to the analyzed cells. **D** Representative phase contrast images, cell migration speed (μm/h), and cell displacement vectors (calculated from consecutive frames acquired 10 min apart) for HMEC-1 in monolayer at t = 10 min and t = 20 h under static (columns 1, 2) or flow exposed conditions (columns 3, 4). Under flow conditions, the jet center is positioned at the center of the full microscopy field of view; for clarity, only the lower-right quadrant is shown here, such that the jet center corresponds to the upper-left corner of the displayed image. White or black contours indicate concentric radial positions spaced 185 μm apart from the jet center and correspond to the dashed lines in (**B**,**C**). Cell displacements are scaled 15x and 30x larger for static and flow-exposed cells, respectively. Scale bar: 300 μm. **E** Cell migration speed (μm/h) over time (h) for HMEC-1 (black) and hCMEC/D3 (red) under static and flow conditions (opaque vs. bright lines). Data represent mean ± SEM of *n* = 10 ± 4 recordings from at least *N* = 3 independent experiments. **F** Same as (**E**) but showing correlation length of cell motion (μm) over time (x-axis, h).

To investigate how flow-driven SSG regulates μEC migration, we used human dermal μEC (HMEC-1) and validated key findings also in human cerebral μEC (hCMEC/D3). These two μEC lines were chosen because they have been widely used for studying wound healing, angiogenesis, and barrier function, and because μEC have been shown to experience varying SS and SSG, unlike macrovascular EC whose shear stresses are more uniform^22^. μEC were cultured in monolayer, and an impinging flow device was used to apply flow while enabling concurrent videomicroscopy^9^. Since μEC experience SS between 1 and 10 dynes/cm², with hypertensive conditions exceeding 30 dynes/cm², we used flow rates mimicking (patho)physiological levels, setting the flow at 3 mL/min^23–25^. This produced the characteristic radially outward flow pattern shown in Fig. 1A, with the corresponding SS and SSG profiles shown in Fig. 1B, C (Supplementary Video 1, part A).

Exposure to flow resulted in an approximately two-fold reduction in μEC migration speed, which remained remarkably stable throughout the 20 h recording period. This reduction in migration speed was accompanied by a pronounced increase in coordination of cell motion, as evidenced by enhanced alignment of neighboring displacement vectors (Fig. 1D, E, Supplementary Fig. 1A and Supplementary Video 2). In contrast, under static (control) conditions, HMEC-1 and hCMEC/D3 displayed random migration with a progressive decrease in migration speed over time, due to increased cell packing likely resulting from cell proliferation (Fig. 1D, E and Supplementary Fig. 1A). To confirm this hypothesis, we first assessed cell density via flow cytometry at the end of the 20 h recordings. HMEC-1 under flow showed 23% lower density compared to static controls (Supplementary Fig. 1B). We also immunostained HMEC-1 samples for the proliferation marker Ki-67 at 10 h and 20 h post flow exposure and observed reduced staining in flow-exposed cells compared to static controls (Supplementary Fig. 1C, D).

Considering that flow-exposed μEC appeared to enhance their coordinated motion, we sought to better quantify this observation by calculating the correlation length of neighboring cell displacement vectors (Fig. 1F)^26^. We found that throughout the course of the recording, both μEC types exposed to flow exhibited a two-fold increase in their correlation length compared to μEC under static conditions. Collectively, these findings demonstrate that flow exposure using the impinging flow device increases μEC migration coordination while reducing and stabilizing migration speed throughout the 20 h of videomicroscopy.

### μEC in monolayer reverse rheotax in response to flow-driven SSG

**Fig. 2:**
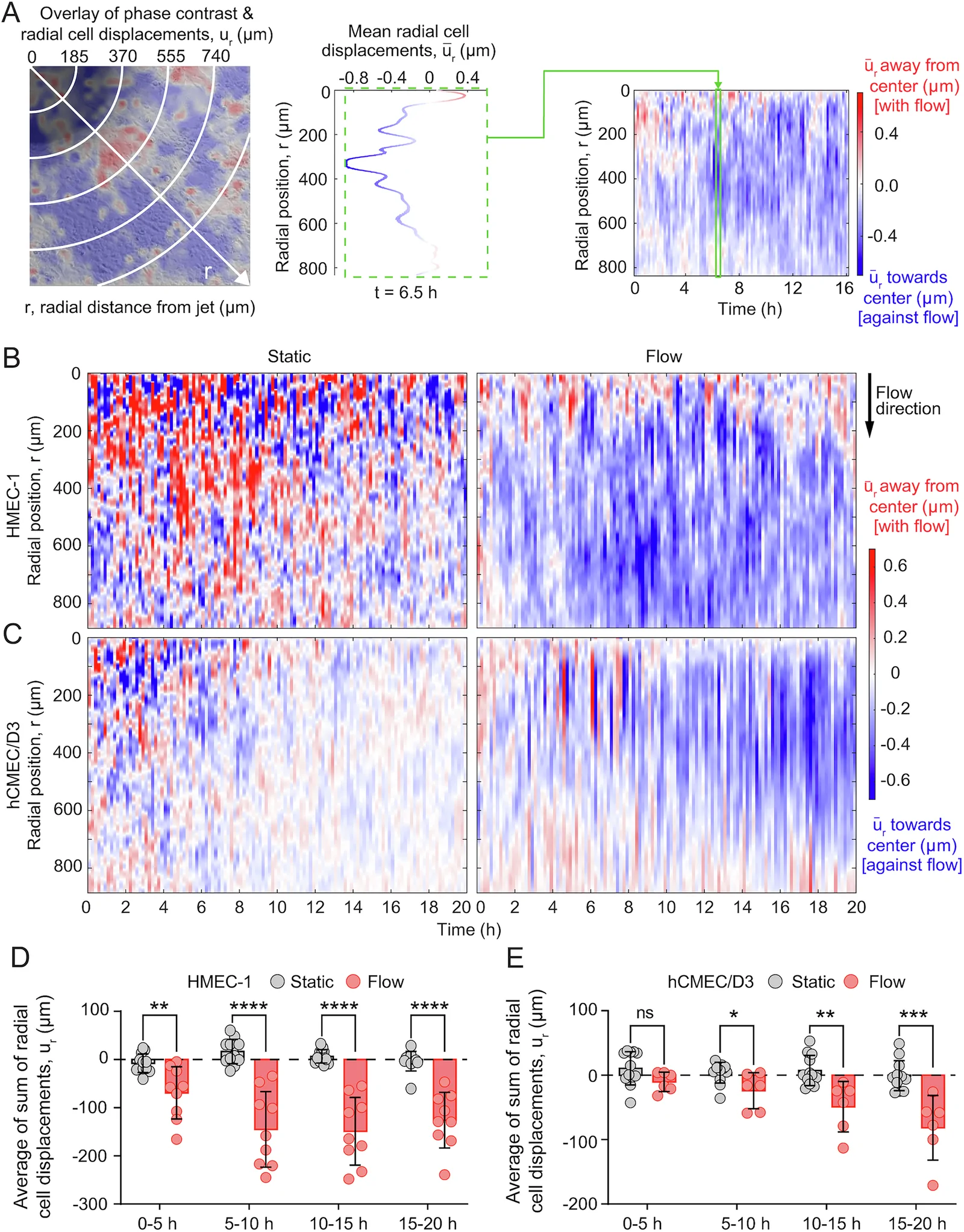
μEC in monolayer exposed to SSG migrate collectively against the flow direction. **A** Left: phase contrast image of HMEC-1 in monolayer with superimposed radial cell displacement map, *u*_*r*_ (μm), where blue indicates cell movement toward the jet center (negative *u*_*r*_) and red indicates movement away from the jet center (positive *u*_*r*_). Middle: Plot of mean *u*_*r*_, along the radial direction, as a function of radial distance from the jet center at a specific time point (blue for negative and red for positive mean *u*_*r*_). Right: Sequential measurements of mean radial cell displacements *u*_*r*_ (μm) stacked next to each other over time (h) enable the construction of a kymograph showing spatiotemporal evolution of radial cell displacements, *u*_*r*_ (y-axis: radial distance r, μm; x-axis: time h). **B** Kymograph of the mean radial cell displacements, *u*_*r*_ (in μm, see colorbar) for HMEC-1 in monolayer under static (left) or flow (right) conditions. The center of the polar coordinate system is at the center of the field of view (where the orifice of the jet is for flow-exposed cells). Kymographs are representative of *n* = 10 recordings on average from at least *N* = 3 independent experiments. **C** Kymograph as panel B but for hCMEC/D3. Kymographs are representative of *n* = 9 recordings on average from at least *N* = 3 independent experiments. **D** Bar plots of the integral of radial HMEC-1 displacements, *u*_*r*_ (μm), over radial distance, averaged over the indicated time periods, for HMEC-1 exposed to flow or maintained under static conditions. Data were binned according to the experimental recording phase: 0–5 h, 5–10 h, 10–15 h, and 15–20 h. Flow was imposed as soon as the recordings were initiated. Superimposed circles refer to average values of independent recordings. An average of *n* = 10 recordings from at least *N* = 3 independent experiments was analyzed. Mean ± SD. WRST, *p* < 0.01: **; *p* < 0.0001: ****. **E** Same as (**D**) but for hCMEC/D3. An average of *n* = 9 recordings from at least *N* = 3 independent experiments was analyzed. Mean ± SD. WRST, *p* > 0.05: non-significant, ns; *p* < 0.05: *; *p* < 0.01: **: *p* < 0.01; ***: *p* < 0.001.

Previous work using an impinging flow device showed that macrovascular EC typically migrate with the flow (forward rheotaxis), while lymphatic μEC migrate against it (reverse rheotaxis)^9^. We observed that displacement vectors of both HMEC-1 and hCMEC/D3 pointed toward the jet center under flow, suggesting they undergo reverse rheotaxis, and sought to systematically investigate this behavior in a spatiotemporal manner. Exploiting the axisymmetric nature of the impinging flow, we quantified mean radial cell displacements (*u*_*r*_) as a function of distance from the jet center (or center of the field of view for static controls) and time. These data are represented as kymographs, with time on the horizontal axis and radial position on the vertical axis (Fig. 2A). This approach enables direct visualization and quantification of rheotaxis, where negative *u*_*r*_ (blue) denotes migration toward the jet center, i.e., against the flow.

Under static conditions, both HMEC-1 and hCMEC/D3 showed random migration with no net directional bias, as expected (Fig. 2B, C). In contrast, during flow exposure, both μEC lines consistently exhibited reverse rheotaxis (blue), with HMEC-1 responding faster (within 0- 5 h post flow exposure as opposed to within 5–10 h post-flow exposure) and showing stronger rheotaxis than hCMEC/D3 (Fig. 2D, E). HMEC-1 rheotaxis peaked in the region between 370 to 555 μm from the jet center, aligning with the region of minimal gradient (Supplementary Fig. 2A). Consistently, imaging fixed HMEC-1 monolayers from the jet center toward the well edge after 20 h of flow revealed a ~ 20% increase in nuclear density within the high-SSG region compared to static controls, whereas cell densities were comparable between conditions at larger radial distances (Supplementary Fig. 2B, C).

To confirm that reverse rheotaxis results from the SSG and not merely from constant SS due to flow, we exposed HMEC-1 to constant SS (10 and 40 dynes/cm²) using parallel flow channels. Under these conditions, cells showed no changes in migration direction or speed (Supplementary Fig. 2D, E). These results demonstrate that spatial SSG, rather than SS of uniform constant magnitude, are required to induce reverse rheotaxis in μEC within the time frame examined ( < 40 h).

### μEC migration speed partially recovers after flow cessation, while rheotactic directionality persists

**Fig. 3:**
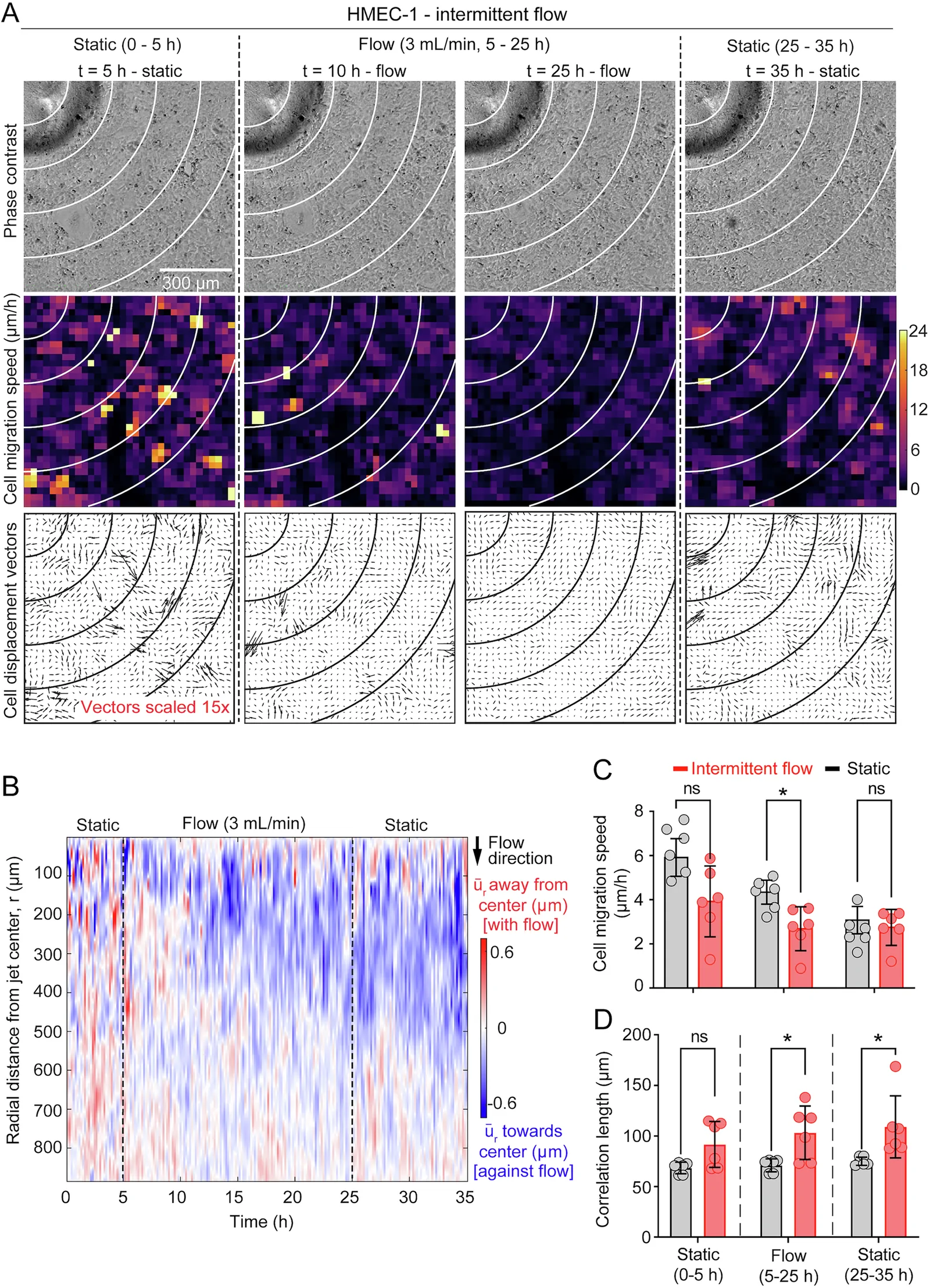
Flow exposure reduces μEC migration speed with gradual recovery after flow cessation. **A** Representative phase contrast images, cell migration speed (μm/h), and cell displacement vectors (scaled 15x) for HMEC-1 in monolayer at different time points. Cells were imaged without flow for ~5 h, followed by 20 h during flow (between dashed black lines) and an additional 10 h under static conditions after flow cessation. Jet center is positioned at the center of the field of view imaged, but only a quarter of the field of view is shown. White or black contours mark concentric regions spaced 185 μm apart from the jet orifice. Scale bar: 300 μm. **B** Kymograph of mean radial cell displacements *u*_*r*_ (μm) over radial distance *r* (μm), for the same representative HMEC-1 recording as shown in panel A. The 20 h flow period is marked by dashed black lines. **C** Bar plot showing the mean cell migration speed (y-axis, μm/h) of HMEC-1 maintained under static conditions throughout the experiment (gray) or exposed intermittently to flow (red). Averaging was performed over three time windows: 0–5 h after the start of recording, during which both groups were under static conditions; 5–25 h, during which the intermittent-flow group was exposed to flow; and 25–35 h, during which both groups were again under static conditions. Mean ± SD, *N* = 6 independent recordings. Superimposed circles show average values for independent recordings. WRST, *p* > 0.05: non-significant, ns; *p* < 0.05: *. **D** Same as (**C**), but showing mean correlation length (y-axis, μm) of cell motion. Mean ± SD, *N* = 6 independent recordings. Superimposed circles show average values for independent recordings. WRST, *p* > 0.05: non-significant, ns; *p* < 0.05: *.

To more unambiguously confirm the reduction in μEC migration speed upon flow exposure and to characterize μEC kinematics after flow cessation, we imaged HMEC-1 for 5 h under static conditions, followed by 20 h of flow exposure (3 mL/min), and then for at least 10 h after flow was ceased (Fig. 3A and Supplementary Video 3). This experimental design allowed us to track identical cells within the same field of view before, during, and after flow, excluding confounding effects from variations in cell density or generational differences. Consistent with our earlier observations, HMEC-1 migration speed decreased rapidly and steadily upon flow onset and cells migrated against the flow direction (Fig. 3B, C). Following flow cessation, migration speed partially recovered over time; however, cells continued to migrate directionally against the former flow direction, indicative of persistent reverse rheotaxis, as observed at 35 h, i.e., 10 h after flow cessation (Fig. 3B, C). Consistent with persistent rheotaxis, the correlation length (measure of coordinated motion among neighboring cells) increased during flow exposure and remained significantly higher than in control HMEC-1 never exposed to flow post-flow cessation (Fig. 3D). A similar behavior was observed in hCMEC/D3 (Supplementary Fig. 3A–D, Supplementary Video 3). Together, these results demonstrate that SSG acutely suppress μEC migration speed but induce a persistent rheotactic and collective migration state that outlasts flow exposure, suggesting that μEC retain a mechanical memory of prior flow exposure.

### μEC increase traction and monolayer stresses steadily in response to flow-driven SSG

To power their motility, adherent cells exert traction stresses on their extracellular matrix (ECM), which can be thought of as a proxy of how well their focal adhesions are organized and connected to the underlying contractile cytoskeleton^27^. To assess whether changes in traction stresses arise in flow-exposed EC, we applied traction force microscopy (TFM) on a monolayer of HMEC-1 seeded on a soft, physiologically relevant 3 kPa hydrogel embedded with fluorescent beads and performed videomicroscopy both before and after 20 h of flow exposure. To verify that tracer bead displacements reflected cell-generated traction rather than flow-induced deformation of the hydrogel, we performed acellular control experiments, which showed negligible bead displacement (Supplementary Video 1, part B).

**Fig. 4:**
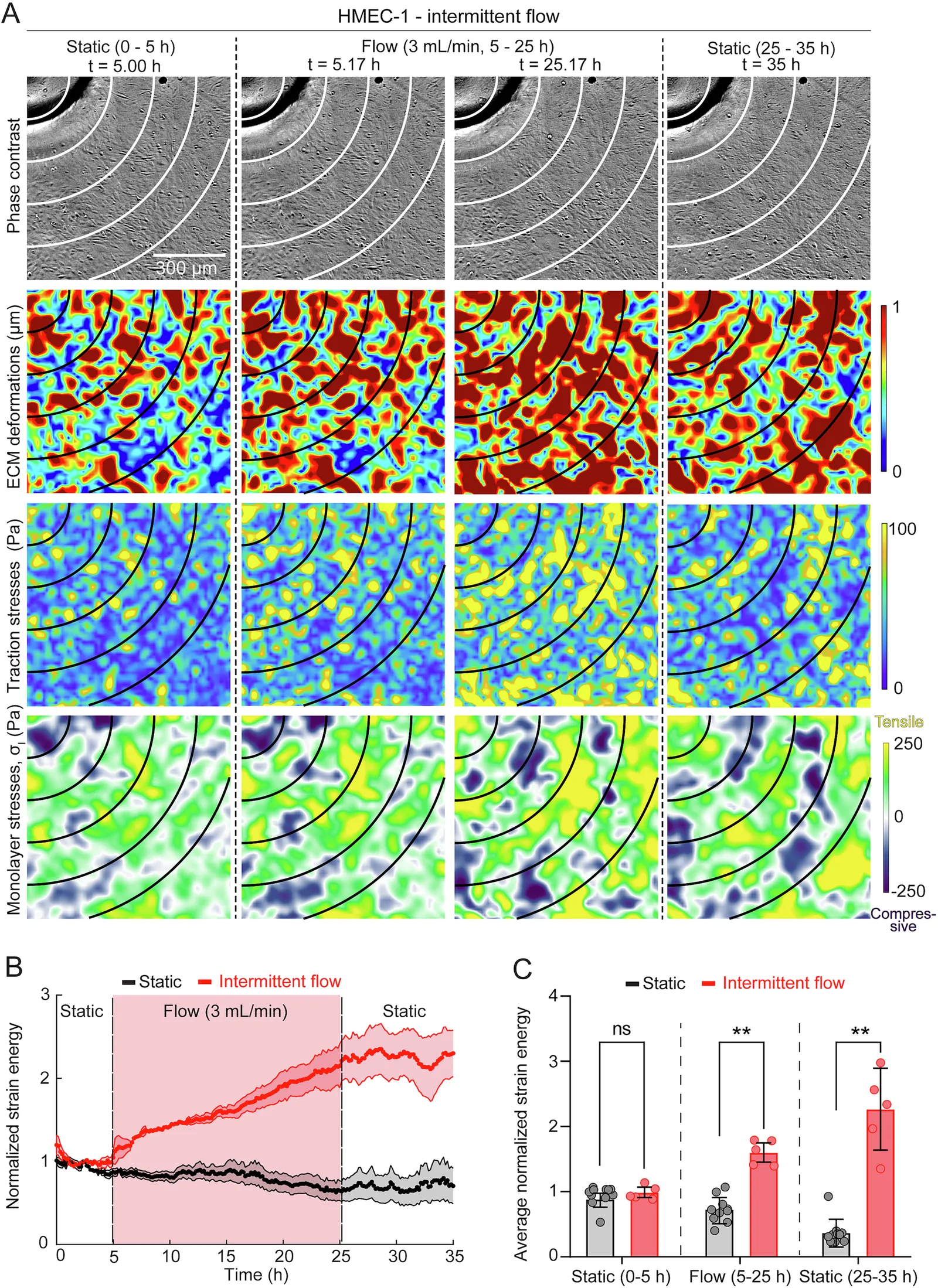
Flow induces a quasi-linear increase in cellular traction and monolayer stresses. **A** Representative images of HMEC-1 in monolayer at various stages: before flow (1st column), immediately after flow initiation (2nd), 20 h into flow (3rd), and 10 h post-flow cessation (4th). Rows show phase contrast (1st), ECM deformations (2nd, μm), cellular traction stresses (3^rd^, Pa), and monolayer tensile stresses, *σ*_*Ι*_ (4th, Pa). Cells on a 3 kPa matrix were exposed to flow using the impinging flow device. Jet center is positioned at the center of the field of view imaged, but only a quarter of the field of view is shown. White or black contours mark concentric regions spaced 185 μm apart from the jet orifice. Scale bar: 300 μm. **B** Normalized strain energy (i.e., mechanical work imparted by HMEC-1) over time. Red line (intermittent flow-exposed) refers to cells that were initially under static conditions (0–5 h), exposed to flow (5–25 h, red shading), and then under static conditions again (25–35 h). Black line refers to control cells never exposed to flow. Mean ± SEM, an average of n = 7 recordings were analyzed originating originating from N = 3 independent experiments. Strain energy was normalized to its value at 2.5 h after the start of the recording, corresponding to the midpoint of the 5-h static phase preceding flow exposure. **C** Bar plots of time-averaged normalized strain energy as in panel B, binned by experimental recording phase: pre-flow (0–5 h), flow (5–25 h), and post-flow (25–35 h). Red bars: intermittent flow-exposed cells; black bars: static controls. Mean ± SD, an average of n = 7 recordings were analyzed originating from *N* = 3 independent experiments. WRST, *p* > 0.05: ns; *p* < 0.01: **.

In the presence of cells, we discovered that upon flow exposure, HMEC-1 in a monolayer immediately increased their traction stresses and strain energy (mechanical work imparted by cells to deform their matrix) by ~ 20% (Fig. 4A-C and Supplementary Video 4). This was followed by a continuous, time-dependent linear increase in traction stresses and strain energy throughout the exposure to flow, with strain energy reaching ~80% higher values at the end of a 20-hour-long recording. Interestingly, after cessation of flow, HMEC-1 continued to exert elevated traction stresses (for at least the following 10 h that the recordings lasted), which suggest that either the cells have been mechanically reprogrammed and/or their matrix remodeled. A similar mechanical response was observed in hCMEC/D3, although post-flow strain energy returned to near pre-flow values (Supplementary Fig. 4A, B). Thus, although traction stresses increased during flow exposure in both EC types, the flow-outlasting dynamics were cell-type-dependent. In contrast to flow exposed EC, static control EC monolayers exhibited traction stresses and strain energy with minimal temporal fluctuations (steady) throughout the recordings (Fig. 4A–C, Supplementary Fig. 4B and Supplementary Video 4).

We also performed monolayer stress microscopy (MSM), to calculate EC monolayer stresses (i.e., inter- and intra-cellular stresses), a measure of endothelial barrier mechanics^28^. Monolayer tensile stresses also increased during flow exposure for both EC types but remained significantly elevated after flow cessation only for HMEC-1 (Fig. 4A and Supplementary Fig. 4A, C). Collectively, these data suggest that there is an intricate link between EC kinematics and dynamics during flow exposure. μΕC respond immediately to fluid flow-generated shear stress gradients by increasing their traction and monolayer stresses and decreasing their speed as they collectively attempt to reverse rheotaxis. In other words, it appears that for reverse rheotaxis to occur, cells need to “grab” strongly onto their ECM and move against the flow. Following flow cessation, however, the persistence of these mechanical changes differs between EC types, with HMEC-1 maintaining elevated traction and monolayer stresses and hCMEC/D3 showing substantial recovery to pre-flow levels.

### SSGupregulatese the expression of focal adhesion, cytoskeletal, and TGF-β1-related genes in μΕC

**Fig. 5:**
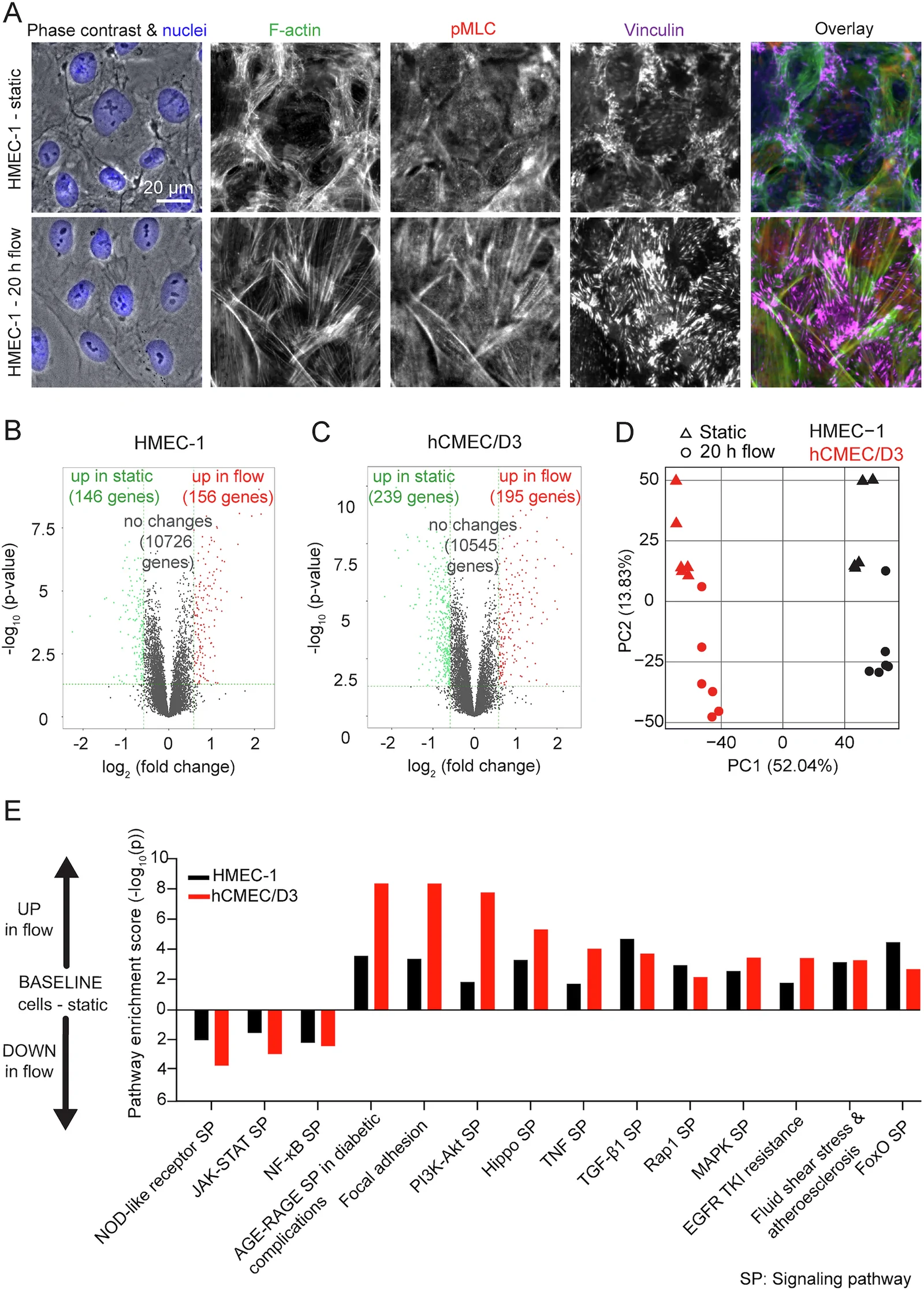
SSG induces alterations in the cytoskeleton and gene expression in μEC. **A** Immunofluorescence images of HMEC-1 cells following static culture (1st row) or 20 h flow exposure (2nd). Columns display: phase contrast (grayscale) overlaid with cell nuclei (blue); F-actin (green in overlay); phosphorylated myosin-light chain (pMLC, red in overlay), vinculin (magenta in overlay); and a composite overlay of these signals. Scale bar: 20 μm. **B-C** Volcano plots of DEGs for HMEC-1 (**B**) and hCMEC/D3 (**C**) exposed for 20 h to shear flow versus not (static condition). The -log_10_ p-values (y-axis) are plotted against the average log_2_-fold changes in expression (x-axis). For each cell type, upregulated genes in flow-exposed cells are shown in red, downregulated in green, and non-DEGs in gray. *N* = 6 biological replicates per condition. **D** PCA of top genes that have ANOVA *p*-value ≤ 0.05 on FPKM abundance estimations. PC1 versus PC2. HMEC-1: black; hCMEC/D3: red; cells under static conditions: triangle; and cells exposed to flow for 20 h: circle. **E** HMEC-1 (black) and hCMEC/D3 (red) cells exposed to shear flow for 20 h were compared with static (no-flow) controls based on their pathway enrichment score (–log_10_p). Shown are pathways that are commonly up- or down-regulated in both cell types with respect to cells under static conditions and have a mean enrichment score > 1.5.

The observed flow-induced increase in traction stresses suggested cytoskeletal reorganization involving focal adhesions and actomyosin contractility. Consistent with this, immunostaining revealed enhanced F-actin stress fibers, increased phosphorylated myosin light chain (pMLC) localization along stress fibers, and a redistribution of vinculin toward basal focal adhesions in flow-exposed μEC, compared with predominantly pericellular vinculin under static conditions (Fig. 5A and Supplementary Fig. 5A, B). To identify mechanotransduction pathways underlying these structural and mechanical changes, we performed RNA sequencing on HMEC-1 and hCMEC/D3 monolayers cultured under static conditions or exposed to flow for 20 h (six biological replicates per condition). Flow exposure induced substantial transcriptional remodeling in both cell types: HMEC-1 exhibited 156 upregulated and 146 downregulated genes, whereas hCMEC/D3 displayed 195 upregulated and 239 downregulated genes, indicating that hCMEC/D3 exhibit greater transcriptional sensitivity to flow than HMEC-1 (Fig. 5B, C).

Principal component analysis (PCA) revealed that the EC type was the primary determinant of transcriptomic variation, accounting for 52% of the total variance, while flow exposure was the second most influential factor, accounting for 13% of variance (Fig. 5D). Given the significant impact of flow on gene expression, we next sought to identify the major biological pathways altered by shear stress. Kyoto Encyclopedia of Genes and Genomes (KEGG) pathway analysis revealed multiple pathways commonly regulated by flow in both cell types (Fig. 5E and Supplementary Data 1). Among these were pathways directly related to endothelial biomechanics and cytoskeletal regulation, including focal adhesion signaling, cytoskeletal regulation pathways (PI3K-AKT and Hippo), and TGF-β1 signaling (Fig. 5E). Several metabolic pathways (AGE-RAGE, mTOR, FOXO), which can indirectly influence mechanotransduction, were also differentially regulated. In contrast, pathways associated with inflammatory responses, such as NF-κΒ, were suppressed during flow, reinforcing the notion that physiological flow exposure may actually be anti-inflammatory. Among the 142 commonly differentially regulated genes in response to flow, 11 encode proteins involved in ECM remodeling, and 23 are associated with TGF-β1 signaling (Supplementary Fig. 5C, D). TGF-β1 is a key cytokine in ECM dynamics that is secreted in a latent form and activated by integrin-mediated cell forces^29^. Immunostaining of TGF-β1 revealed a striated, predominantly pericellular distribution in HMEC-1, without an obvious qualitative increase following flow exposure (Supplementary Fig. 5E). While this staining does not allow us to distinguish between intracellular, extracellular, latent, or active TGF-β1, the transcriptomic data indicate upregulation of the TGF-β1 signaling pathway as a whole under flow conditions. Several mechanobiologically relevant DEGs support activation of this pathway, including THBS1, which mediates shear-dependent TGF-β1 activation^30, 31^, JUNB, a regulator of actomyosin contractility^32, 33^, PDGFB, a mechanosensitive growth factor induced by shear stress^34–36^, SMAD7 a regulator of TGFβ signaling^37^, IL11, a pleiotropic cytokine secreted in response to TGFβ signaling^38^, and PTGS2, an enzyme rich in breast tumors that expresses high levels of TGF-β1^39^. Conversely, CAV1 (Caveolin-1), which is involved in caveolae formation and mechanosensing, was downregulated, consistent with its suppression by TGF-β1 signaling^40^. Rac2, a small GTPase linked to actin cytoskeleton remodeling, was also downregulated^41^.

Collectively, the transcriptomic analysis suggests that focal adhesion remodeling, cytoskeletal reorganization, and TGF-β1 signaling may be central drivers of μEC adaptation to flow-induced SSG.

### FAK inhibition reverses μΕC rheotaxis, while ROCK inhibition only slows migration

**Fig. 6:**
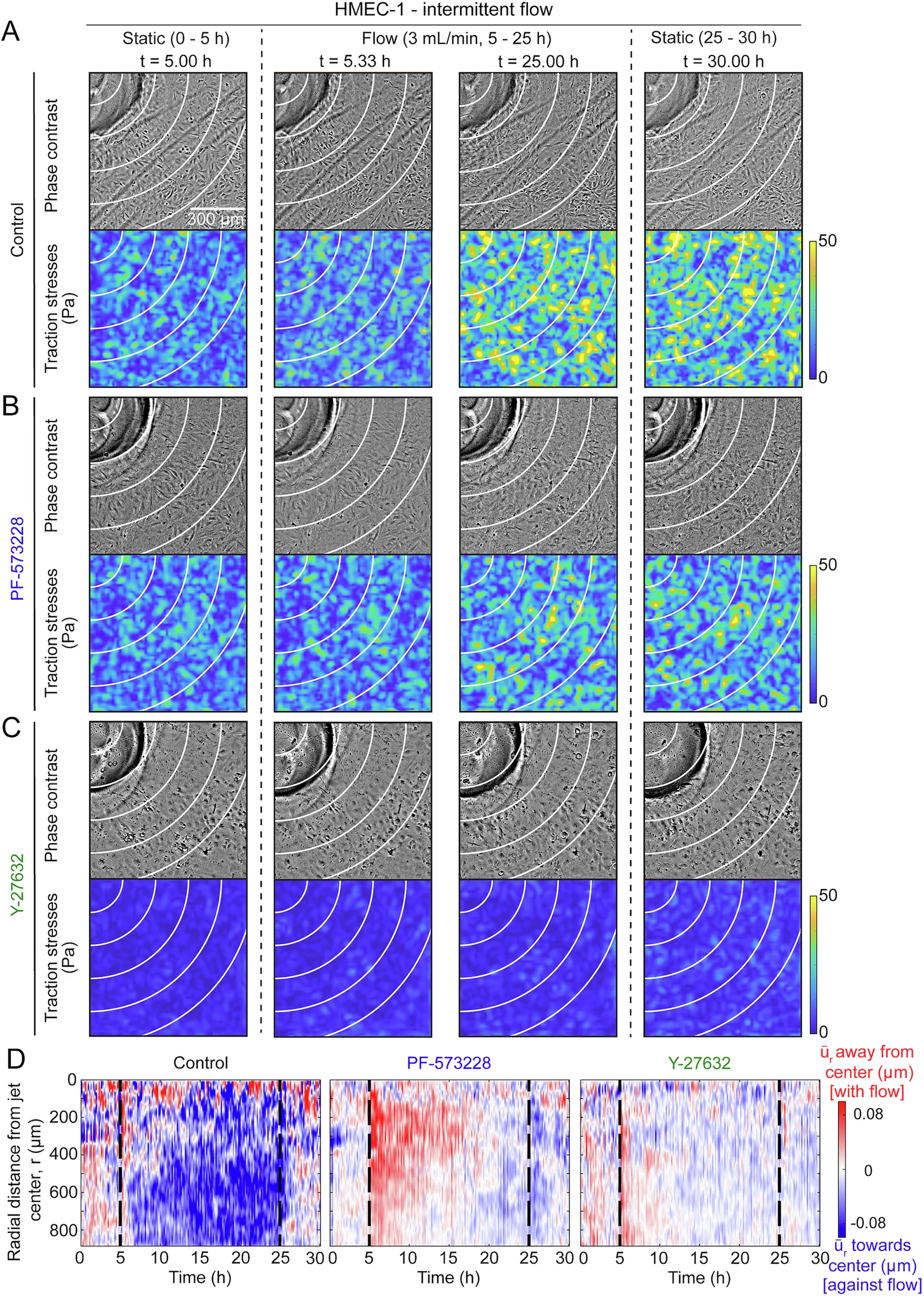
The FAK and ROCK signaling pathways regulate μΕC rheotaxis and dynamics. **A** Representative images of HMEC-1 in monolayer at various stages: before flow exposure (1st column), immediately after flow initiation (2nd), 20 h into flow (3rd), and 5 h post-flow cessation (4th). Rows show phase contrast (1st) and cellular traction stresses (2nd, Pa). Cells on a 3 kPa matrix were exposed to flow using the impinging flow device. Jet center is positioned at the center of the field of view imaged, but only a quarter of the field of view is shown. White contours mark concentric regions spaced 185 μm apart from the jet orifice. Scale bar: 300 μm. **B**,**C** Same as panel A but for cells treated with 5 μM PF-573228 (**B**) or 10 μM Y-27632 (**C**). **D** Kymograph of mean radial cell displacements *u*_*r*_ (μm) over radial distance *r* (μm), for HMEC-1 in monolayer for the representative recordings shown in (**A–C**). In the left kymograph, cells were treated with vehicle control, middle kymograph with PF-573228 and right kymograph with Y-27632. The flow exposure period is indicated between black dashed lines.

Based on the observed flow-induced remodeling of focal adhesions and the actomyosin cytoskeleton, we next examined their role in driving reverse rheotaxis and the gradual increase in EC strain energy and mechanistic coupling (Supplementary Fig. 6A). To perturb focal adhesion signaling, HMEC-1 were treated with the focal adhesion kinase (FAK) inhibitor PF-573228, a potent FAK inhibitor with substantially lower activity toward Pyk2 and other tested kinases^42^, two hours before initiation of videomicroscopy on cells exposed to intermittent flow. FAK inhibition suppressed neither traction force generation before flow exposure, with mean traction stresses remaining comparable to vehicle control, nor traction force increase upon flow exposure and linear increase thereafter (Fig. 6A, B, Supplementary Fig. 6E–G and Supplementary Video 5). However, traction adhesion turnover (i.e., the extent to which active focal adhesions bearing force renew) was significantly slower upon FAK inhibition (Supplementary Fig. 7A) and was accompanied by a decrease in cell migration speed (Supplementary Fig. 6D). Notably, PF-573228-treated cells failed to exhibit reverse rheotaxis and instead migrated in the direction of flow (Fig. 6D and Supplementary Fig. 6B, C). To strengthen the pharmacological evidence for FAK involvement, we additionally tested FAK-14, which similarly impaired reverse rheotaxis (Supplementary Fig. 7B–D)^43^. Thus, although FAK activity does not regulate the magnitude of traction forces under either static or flow conditions, it is required for dynamic traction adhesion remodeling and for maintaining upstream-directed migration under flow. Thus, these findings implicate FAK signaling in both adhesion dynamics and rheotactic directionality

To examine the contribution of actomyosin contractility, HMEC-1 were treated with the ROCK1/2 inhibitor Y-27632, which can also inhibit PKN/PRK2 and, at higher concentrations, other kinases such as PKC and PKA^44, 45^. Treatment of HMEC-1 with Y-27632 significantly reduced the magnitude of cell-generated traction stresses, consistent with previous studies (Fig. 6C and Supplementary Fig. 6E)^46, 47^. Upon flow exposure, an immediate increase in traction forces was still observed in Y-27632-treated cells, suggesting that ROCK-independent contractile mechanisms must regulate it (Supplementary Fig. 6F). However, traction magnitude remained constant over time during flow exposure in Y-27632-treated cells, unlike vehicle control-treated cells (Supplementary Fig. 6G and Supplementary Video 5). Traction adhesion turnover was also markedly reduced, to an extent comparable to or greater than that observed following FAK inhibition (Supplementary Fig. 7A), consistent with the established role of ROCK-dependent cytoskeletal tension in focal adhesion maturation and dynamics^48, 49^. Y-27632 treatment also reduced the mean migration speed by approximately 50%, similar to FAK inhibition (Supplementary Fig. 6D). However, unlike FAK-inhibited cells, ROCK-inhibited cells retained the ability to migrate against the direction of flow, albeit at a reduced speed (Fig. 6D and Supplementary Fig. 6B, C).

Together, these results demonstrate that FAK signaling and actomyosin contractility play distinct roles in μEC rheotaxis. Actomyosin contractility regulates the speed of reverse rheotaxis only, whereas FAK signaling is required for rheotaxis directionality. Importantly, neither the absolute magnitude of traction stresses nor reduced traction adhesion turnover alone is sufficient to explain rheotactic directionality, pointing instead to a specific requirement for FAK signaling in establishing or maintaining upstream-directed migration.

### TGF-β1 drives μΕC traction strengthening during flow independent of rheotactic response

**Fig. 7:**
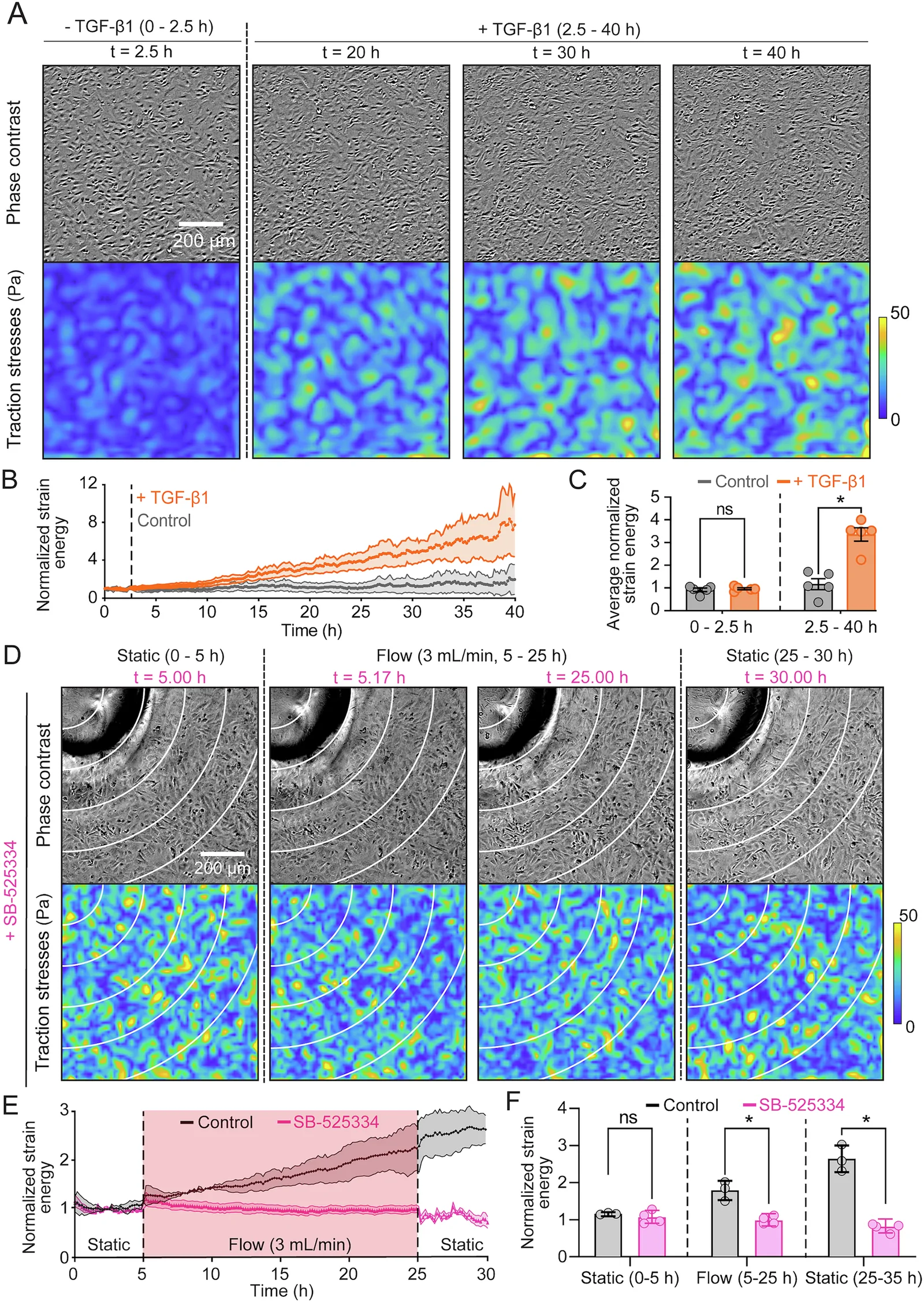
The increase in EC traction stresses upon flow exposure depends on TGF-β1. **A** Representative images of HMEC-1 in monolayer under static condition in the presence of 10 ng/mL TGF-β1. Note that TGF-β1 was added 2.5 h post-initiation of the microscopy recording. Columns show different time points. Rows show corresponding phase contrast image (top) and cellular traction stresses (bottom, Pa). Scale bar: 200 μm. **B** Normalized strain energy of HMEC-1 in monolayer under static conditions treated with vehicle control (gray) or 10 ng/mL TGF-β1 (orange) over time (h). Strain energy is normalized to the first value at the beginning of each recording. Mean ± SEM, *N* = 4 independent experiments. **C** Bar plot of average normalized strain energy of data shown in (**B**) binned by time periods of 0-2.5 h (no TGF- β1) and 2.5-40 h (addition of TGF- β1) for HMEC-1 treated with TGF-β1 (orange) or not (gray). Mean ± SD, N = 4 independent experiments. WRST, *p* > 0.05: ns; *p* < 0.05: *. **D** Representative images of HMEC-1 in monolayer treated with 10 μM SB-525334 at different instant of time, namely: before flow (1st column), immediately after flow initiation (2nd), 20 h into flow (3rd), and 5 h post-flow cessation (4th). Rows show phase contrast (1st) and cellular traction stresses (2nd, Pa). Cells on a 3 kPa matrix were exposed to flow using the impinging flow device. Jet center is positioned at the center of the field of view imaged, but only a quarter of the field of view is shown. White contours mark concentric regions spaced 185 μm apart from the jet orifice. Scale bar: 200 μm. **E** Normalized strain energy of HMEC-1 in monolayer treated with vehicle control (black) or 10 μM SB-525334 (magenta) over time (h). Strain energy was normalized to its value at 2.5 h after the start of the recording, corresponding to the midpoint of the 5-h static phase preceding flow exposure. Mean ± SEM, *N* = 3 and 4 independent TFM recordings, respectively. Time of flow exposure is shown in red shade. **F** Bar plot of average normalized strain energy of data shown in (**E**) binned by time periods of 0–5 h (static), 5–25 h (flow) and 25–30 h (static) for exposed to SB-525334 (magenta) or vehicle control (black). Mean ± SD, *N* = 3 and 4 independent TFM recordings, respectively. WRST, *p* > 0.05: ns; *p* < 0.05: *.

Since FAK but not ROCK inhibition failed to suppress the linear increase in traction stresses in flow-exposed μEC (Supplementary Fig. 6G), we next examined whether TGF-β1 signaling contributes to flow-induced traction strengthening. This was motivated by prior work showing that recombinant TGF-β1 enhances cellular traction forces in other cell types, while decreased traction of ROCK-inhibited cells abrogates the activation of TGF-β1 signaling^32^. Consistently, treatment of HMEC-1 monolayers with recombinant TGF-β1 under static conditions induced a progressive increase in traction force magnitude over time (Fig. 7A, B). Quantification revealed a ~ 300% increase in strain energy after 20 h of exposure (Fig. 7C). At later time points ( > 30 h), cells became highly elongated and exhibited nematic alignment across the monolayer (Supplementary Video 6), consistent with long-term EC cytoskeletal and matrix remodeling.

To examine how inhibition of TGF-β1 signaling impacts EC traction force generation during flow exposure and rheotaxis, we exposed HMEC-1 to flow in the presence of SB-525334, which inhibits preferentially the TGF-β type I receptor (TβRI), but also the closely related ALK4 receptor; thus, at the concentration used here, its effects cannot be attributed exclusively to inhibition of TβRI (alias ALK5)^50^. SB-525334 treatment did not alter the initial acute increase in traction stresses compared to vehicle-control-treated cells but abolished the linear increase in traction stresses during flow (Fig. 7D–F and Supplementary Fig. 8A). A similar suppression of progressive traction strengthening was observed with the structurally distinct TβRI inhibitor LY-364947 (Supplementary Fig. 8E, F), further supporting a requirement for TGF-β signaling in this response. These findings indicate that intact TGF-β1 signaling is required for the progressive traction strengthening but not the acute traction response to flow. In contrast, neither mean migration speed nor backward rheotaxis behavior was significantly altered compared with vehicle control-treated HMEC-1 (Supplementary Fig. 8B–D, G).

Together, these results demonstrate that TGF-β1 signaling is necessary and sufficient for the progressive increase in μEC traction forces observed under flow, but this increase is dispensable for reverse rheotaxis. Thus, traction force strengthening during flow exposure and rheotaxis are likely regulated by distinct mechanotransduction pathways.

## Discussion

We report that two distinct μEC types (dermal- and brain-derived) respond to SSG by acutely slowing down and increasing their motion coordination while exhibiting backward rheotaxis. This response is associated with an immediate, time-dependent linear increase in traction and monolayer stresses. Importantly, the flow-induced kinematic response persists in both μEC types for at least 10 h after flow cessation, with cells continuing to migrate in the direction established during flow exposure. In contrast, the persistence of the dynamics is cell-type-dependent: HMEC-1 maintain elevated traction stresses, whereas hCMEC/D3 substantially recover toward static levels following flow cessation. Thus, prior flow exposure leaves a persistent imprint on μEC behavior, although its manifestation at the level of cell dynamics differs between EC types.

The acute increase in traction stresses observed in flow-exposed μEC is independent of ROCK-mediated contractility or FAK-dependent adhesion reinforcement, motivating the question of what triggers this increase. One speculation is engagement of mechanosensitive elements such as the PECAM-1/VE-cadherin/VEGFR2 complex^11, 51^ or mechanosensitive ion channels (e.g., Piezo1)^52, 53^, both of which can alter intracellular tension via calcium influx or myosin light-chain-independent mechanisms, preceding canonical ROCK/FAK signaling^54^. Future studies could address this using Piezo1 inhibitors (e.g., GsMTx4) or genetic knockdown to determine whether mechanosensitive channel activity is required for the acute traction response. Alternatively, traction forces could increase through conformational changes in integrins or adapter proteins (e.g., talin unfolding) that enhance force transmission to the matrix at existing adhesion sites, bypassing FAK-dependent signaling^55^. In either case, the acute rise in traction stresses likely reflects an active mechanical stabilization of cell-substrate force transmission, enabling EC to rapidly accommodate the sudden and spatially heterogeneous mechanical loads imposed by SSG before slower cytoskeletal and adhesive remodeling takes place.

Concurrently, μEC exhibit an acute but sustained reduction in migration speed accompanied by increased coordination of collective motion as they undergo reverse rheotaxis in response to flow-driven SSG. These changes in cell kinematics occur immediately upon flow exposure, similar to the acute increase in traction stresses. Importantly, these rapid kinematic and dynamic responses are distinct from the slower morphological adaptations of EC to flow, which occur over longer timescales^11, 56^. Such rapid changes in migratory behavior raise the question of how EC sense and establish directionality in response to flow. Reverse rheotaxis has been reported only for cells exposed to uniform SS and for lymphatic EC subjected to SSG^10, 12^, raising the question of what mechanical and molecular signals dictate whether cells migrate with or against the flow direction. From a mechanical perspective, our experimental geometry does not allow us to determine whether the sign of the SSG contributes to this response, as SSG sign and magnitude cannot be independently varied. Resolving this question will require flow geometries that enable independent control of these parameters^6^. Similarly, although our data indicate that TGF-β1 signaling contributes to the flow-induced increase in traction stresses rather than providing the directional cue for reverse rheotaxis, we cannot exclude that spatial gradients of other flow-transported or secreted molecules contribute to directional migration. Resolving this possibility would require direct measurements of soluble-factor distributions or dedicated transport simulations within the impinging-flow geometry.

At the molecular level, some studies suggest that spatiotemporal activation of both Rho and Rac1 (through PECAM-1 mechanosensing) is required for alignment of EC in the direction of flow and establishment of polarity^54^. Other studies suggest that front-rear polarization in response to flow in varying contexts depends both on Rac1 and FAK signaling^57–59^. S1P/S1PR1 signaling has also been shown to activate FAK and promote focal adhesion remodeling and cytoskeletal reorganization in EC, thereby enhancing endothelial barrier function^60^. Importantly, upstream migration of lymphatic EC under flow has been reported to depend on S1PR1 signaling^61^, raising the possibility that S1PR1 may act upstream of FAK as part of a broader mechanotransduction pathway regulating directional endothelial migration. This is consistent with our findings, where pharmacological inhibition of FAK inhibits reverse rheotaxis, with cells exhibiting forward rheotaxis albeit with reduced speed. The mechanism by which FAK signaling encodes flow directionality remains, though unclear. One possibility is asymmetric focal adhesion turnover, with preferential dynamics on the upstream cell edge. Alternatively, direction may be sensed through other mechanisms (glycocalyx, Piezo1), with FAK acting downstream to execute directional migration. Interestingly, FAK inhibition does not impact the magnitude of traction stresses of cells under static or flow conditions compared to controls, consistent with FAK inhibition not eliminating the mechanochemical responses associated with force generation, as recently reported^62^. However, we find that FAK inhibition significantly reduces the turnover of active traction adhesions.

Our observation that ROCK-inhibited cells remain capable of rheotaxis despite exhibiting a ~ 10-fold reduction in strain energy compared to controls, together with the absence of a linear increase in traction stresses during prolonged flow exposure, indicates that the magnitude of traction forces per se does not determine whether reverse rheotaxis will occur. Moreover, this decoupling raises a related question: why do traction forces progressively increase during flow exposure, and what mechanisms regulate this response if it is dispensable for directional migration? Our transcriptomic and imaging analyses identify TGF-β1 signaling as a key driver of the flow-induced, time-dependent increase in traction stresses independently of reverse rheotaxis, consistent with previous observations in adult fibroblasts^63^.

Since this linear traction stress increase was suppressed upon ROCK inhibition, it is possible that ROCK-inhibited cells are unable to effectively activate latent TGF-β1, which in turn enhances actomyosin contractility and EC traction generation, further reinforcing TGF-β1 activation^64–66^. When contractility is suppressed, as under ROCK inhibition, traction levels may fall below the threshold required to sustain this feedback loop. This interpretation is consistent with previous reports showing shear-dependent activation of latent TGF-β1 via integrins, as well as shear-regulated TGF-β1 expression and signaling in vascular EC^64^ and mesenchymal progenitor cells^67^.

Interestingly, some flow-induced responses persisted after removal of the mechanical stimulus, although the extent of this persistence differed between EC types. HMEC-1 retained elevated traction stresses for at least 10 h after flow cessation, whereas hCMEC/D3 showed substantial recovery toward static levels, even though both EC types continued to migrate towards the jet center. These findings suggest that prior flow exposure can induce a persistent biomechanical state that varies across EC types. In HMEC-1, the prolonged elevation of traction stresses is consistent with a form of mechanical memory of prior flow exposure. Two, non-mutually exclusive, mechanisms could underlie this behavior. First, EC may undergo sustained intracellular reprogramming as a consequence of prior flow exposure, which could be addressed in the future by transcriptomic screening of EC at later times after flow cessation. Alternatively, flow may remodel the ECM of EC, thereby altering the mechanical context to which cells subsequently respond. This hypothesis is supported by our finding that flow upregulates genes related to ECM production. Mechanical memory effects on cells have been reported in other systems, including cells exposed to defined matrix stiffness histories or cyclic stretch^68, 69^, and mechanical preconditioning of EC is known to enhance barrier integrity, a principle leveraged in the development of implantable vascular grafts^70^. Moreover, transient exposure to low shear stress was recently shown to exert long-lasting effects on EC traction forces and alignment under subsequent high-shear conditions^71^. Together, these observations support the idea that transient extracellular physical cues can durably shape EC future behavior, while our findings indicate that the persistence of such effects varies substantially between EC types^71^.

Although not explored in depth here, differences between the two μΕC types were evident also in other responses. In particular, hCMEC/D3 were transcriptionally more sensitive to flow than HMEC-1 cells, yet, in contrast to HMEC-1, they rapidly returned their traction stresses to baseline following flow cessation. This divergence suggests vascular bed-specific differences in how μΕC integrate and respond to mechanical cues, which may reflect distinct mechanosensitive programs tailored to the physiological demands of each bed. A limitation of the present study is that both HMEC-1 and hCMEC/D3 are immortalized EC lines and may not fully recapitulate the junctional and barrier properties of primary endothelium, despite expressing canonical endothelial junctional markers; future studies using primary EC from different vascular beds will therefore be important to establish the generalizability of these responses^72, 73^.

The videomicroscopy recordings in this study were performed with cells residing either on glass or on compliant and more physiological soft 3 kPa substrates^74^. Interestingly, we found that HMEC-1 cells migrated more slowly on 3 kPa substrates than on glass (Fig. 3C and Supplementary Fig. 6D), highlighting the sensitivity of EC migratory behavior to the mechanical properties of their environment. Since matrix stiffness can modulate endothelial motility, mechanosensitivity to flow, and cytoskeletal organization, with FAK activation typically enhanced on stiffer matrices^75^ where cells exert increased traction stresses^27^, future studies may address how substrate stiffness modulates flow-guided directional migration. A further methodological limitation concerns MSM, which was performed on image regions that did not encompass the entire monolayer. Consequently, the boundary conditions required for stress reconstruction represent an approximation and may influence the reconstructed absolute stress values, particularly near the image boundaries^76^. Nevertheless, because identical imaging geometries and analysis parameters were applied across conditions, relative comparisons between conditions remain informative.

Finally, transcriptomic analyses revealed that the absence of flow is associated with the upregulation of pathways linked to inflammation and antiviral responses, including interferon signaling as well as NF-κB, JAK-STAT, and NOD-like receptor signaling pathways. These pathways have been implicated in increased endothelial permeability and barrier dysfunction^77–79^, underscoring the critical role of shear flow in maintaining endothelial homeostasis and the importance of incorporating physiologically relevant flow conditions in vitro.

In summary, our findings position spatial SSG as a critical determinant of μΕC behavior, capable of reprogramming both cell kinematics and dynamics, and, in an EC-type-dependent manner, inducing mechanical changes that persist beyond the period of flow exposure. These insights may help explain the spatial heterogeneity of endothelial phenotypes observed in vascular disease. Future studies could explore whether similar SSG-driven adaptations occur in vivo, such as at vessel bifurcations, high-curvature regions, or stenotic lesions, how pulsatile or oscillatory components modulate these responses, and whether interventions targeting FAK or TGF-β1 pathways can restore physiological flow responses in diseased states.

## Materials and methods

### Cell culture conditions

HMEC-1 (a generous gift from the Welch lab, University of California, Berkeley) were cultured in MCDB 131 medium (Gibco, 10372019) supplemented with 10% FBS (Gibco, 21083027), 10 ng/mL epidermal growth factor (Thermofisher, PHG0311), 1 μg/mL hydrocortisone (Fisher, 11444942), and 2 mM L-glutamine (AppliChem, A3704). hCMEC/D3 (a generous gift from the Morrison lab, University of Stuttgart) were cultured according to the manufacturer’s instructions (EndoGRO basal medium and EndoGRO MV supplement kit: Millipore, SCME-BM and SCME004-S) with an additional 1 ng/mL fibroblast growth factor (Sigma, GF003).

### Videomicroscopy recordings

For all videomicroscopy experiments, we acquired time-lapse phase contrast images of cells (power 15%, exposure time 100 ms) using an inverted Nikon Eclipse Ti2 epifluorescence microscope equipped with a Prime 95B camera (Teledyne Photometrics), and a 10x Plan Fluor DL objective with NA = 0.13. The time interval between subsequent image acquisition was always 10 min. The microscope was controlled by the NIS-Elements software (Nikon). Temperature was maintained at 37 °C throughout the experiment by surrounding the microscope with a box-type incubator (OKOlab). For the traction force microscopy recordings described below, we additionally acquired images of the 0.5 μm fluorescent tracer beads embedded within the hydrogel on which cells were seeded (FITC filter cube with 470/40 excitation and 525/50 emission filters). To inspect the radial direction of the fluid flow produced by the impinging flow device, we performed cell-free experiments with 3 μL of a 2% solution of 2.0 μm red fluorescent tracer beads (Invitrogen, F8826) added to 60 mL of cell culture medium used to prime the device. The flow speed was set up to 3 mL/min and images of the beads were taken every 30 ms for a total time of 12 s (mCherry filter cube with 562/40 excitation and 624/40 emission filters). The center of the jet was located in the center of the field of view imaged.

### Reagents for pharmacological perturbations

Pharmacological inhibitors used during imaging were: Y-27632 (target: ROCK inhibitor, concentration applied to cells: 10 μM, stock concentration: 30 mM in DMSO, source: Tocris, 1254/10); SB-525334 (target: TGF-Smad inhibitor, concentration applied to cells: 10 μM, stock concentration: 30 mM in DMSO, source: Biotechne, 3211); PF-573228 (target: FAK inhibitor, concentration applied to cells: 5 μM, stock concentration: 100 mM in DMSO, source: Sigma, PZ0117-5MG); FAK-14 (target: FAK inhibitor, concentration applied to cells: 5 μM, stock concentration: 10 mM in DMSO, source: Sigma-Aldrich, SML0837); LY-364947 (target: TGF-β Type I receptor kinase (ALK5) inhibitor, concentration applied to cells: 10 μM, stock concentration: 15 mM in DMSO, source: Sigma-Aldrich, L6293). All inhibitors in experiments involving flow exposure were added 2 h prior to initiation of videomicroscopy recordings. Recombinant TGF-β1 was added to the cells at the final concentration of 10 ng/mL 2 h prior to initiation of videomicroscopy recordings (stock concentration: 100 μg/mL in 4mΜ HCl containing 0.1% BSA, source: R&D systems, 240-B-002).

### Perfusion of impinging flow device

One day prior to the application of perfusion, endothelial cells were seeded onto a 20 mm 6-well plate with a 1.5 glass coverslip bottom (Cellvis, P06-20-1.5-N). The coverslips were precoated for 30 min at 37 °C with rat tail collagen I (Gibco, A10483-01) diluted 1:100 in PBS (Gibco, 10010023). Excess collagen was aspirated, and wells were washed once with PBS before plating 4 × 10^5^ cells per well (HMEC-1) or 3 × 10^5^ cells per well (hCMEC/D3) in 500 μL of the corresponding medium. Cells were allowed to adhere, and subsequently each well was filled with an additional 2 mL of medium. After reaching confluence, cells were transferred to CO_2_-independent Leibovitz’s L-15 medium (Gibco, 21083027) with the corresponding supplements and kept for 2 h at 37 °C to equilibrate. To acquire images of cells exposed to flow, the 6-well plate with the impinging flow jets on top (orifice located 1 mm above the glass coverslip) was placed on the insert stage of our epifluorescence microscope (Nikon Ti2 Eclipse), and perfusion was started with a flow rate of 3 mL/min (or static/no flow) and maintained for the duration of 20 h.

Each time the impinging flow jet device was used, priming was performed before starting cell perfusion experiments with cell culture medium to remove air bubbles and establish continuous flow^9–11^. For priming, the device was placed on a 6-well plate containing 10 mL of culture medium per well, and medium was circulated until all tubing was filled. The pump was then stopped, and any remaining air was removed through the evacuation port using a syringe. The primed impinging flow device was subsequently transferred onto the 6-well plate containing the cells. Unless otherwise indicated, experiments were performed at a flow rate of 3 mL/min. After each experiment, the impinging flow device was cleaned by sequentially perfusing two 6-well plates containing sterile water (10 min each), followed by one plate containing 10% hydrogen peroxide (30 min), and finally two additional plates containing sterile water (10 min each). Air bubbles were removed through the evacuation port before each perfusion step^9–11^. The simulation of flow pattern and fluid forces generated by the impinging flow device was performed in COMSOL Multiphysics. For the flow pattern simulation, the geometry and material modules were used^9^. For fluid force simulation, the modules for flow simulation, geometry reconstruction, and flow pattern calculation were used^9^.

### Image processing, analysis of cell kinematics and kymographs of cell displacements

Phase contrast images from videomicroscopy recordings were pre-processed with the Fiji/ImageJ software^80^ and analyzed using custom MATLAB (Mathworks) scripts. The codes for this analysis are available in our GitHub repository (see DOI: doi.org/10.5281/zenodo.7155531). Using MATLAB, background and flatfield corrections were applied using a pseudo-flatfield correction with a Gaussian filter and a standard deviation of 40 pixels. Cell displacements were then calculated over time using a custom script for particle image velocimetry (PIV) in MATLAB^81^. For PIV on the phase contrast images, we always used a window size of 48 px and an overlap of 24 px^82^. Displacements on the Cartesian coordinate system were calculated and plotted next to the corresponding phase contrast images using vector arrows. To spatially relate the analyzed cells to the local flow conditions, concentric contours centered on the stagnation point (r = 0) were overlaid on the microscopy images at radial intervals of 185 μm. These contours correspond directly to the radial positions indicated by the vertical dashed lines in the SS and SSG profiles in Fig. 1B, C, thereby allowing the local SS and SSG experienced by cells at different positions within the field of view to be inferred. The same spatial mapping was used throughout all analyses performed based on videomicroscopy (i.e., also for TFM recordings).

We then moved from a Cartesian coordinate system to a polar one, to be able to calculate radial displacements and depict mean radial displacements over time using a kymographic representation. Average radial displacements (u_r_) of the cells are shown as a function of distance from the jet orifice (which is always located at the center of the field of view imaged) and over time, with negative displacements indicating cell migration towards the jet center, that is, against the flow, and positive displacements indicating migration away from the center, that is, in the direction of the flow. Finally, using an additional custom-built script in MATLAB (MathWorks), we calculated correlation length and cell migration speeds. Correlation length was calculated as the spatial correlation function ${C}_{{dd}}\left(R\right)$ that averages the scalar product of all vector pairs separated by a distance R. The correlation values show an exponential decay over distance that can be fitted to the function ${C}_{{dd}}\left(R\right)=\exp (-R/R0)$, where 2R0 indicates the maximum non-random correlated distance^26, 83^. Cell migration speed was calculated as the median value of the distribution generated from the magnitude of the displacement vectors (through PIV) divided by the time between successive frames (10 min). Median values of cell migration speed for each time point and for multiple independent experiments were visualized using Graphpad Prism v10, showing the mean value for different experiments ± standard error of the mean (SEM). To assess how time and radial position impact the direction of cell migration of flow-exposed cells, the calculated kymographs were further processed. First, we calculated the sum of the mean radial cell displacements across all the radial positions for a given time point and plotted it as a function of time (Fig. 2A). A similar procedure was followed to quantify the impact of shear stress magnitude on cell migration direction (related to the radial position). In particular, all mean radial displacements across all time points were summed up and plotted as a function of radial position (Supplementary Fig. 2A). Finally, to enable statistical comparison of directional migration between experimental conditions and across different phases of the recording, radial cell displacements (*u*_*r*_) were integrated over radial distance and averaged within four consecutive 5-h time intervals: 0–5 h, 5–10 h, 10–15 h, and 15–20 h (Fig. 2D, E). The resulting values were used for statistical comparisons of e.g., the kinematic behavior between flow-exposed and static control cells.

### Perfusion of ibidi flow device and image analysis

We used a µ-Slide VI 0.4 glass bottom channel slide pre-coated with Collagen I (ibidi GmbH; 80606; channel volume 30 μL, height 0.4 mm, width 3.8 mm) to seed cells and subsequently expose them to constant magnitude shear stress. In each channel, we seeded 1×10^5^ cells in 30 μL (HMEC-1). The cells were let to adhere for ~ 30 min, and then additional 60 μL of cell culture medium was added to the inlet and outlet of each of the six channels. Twenty-four hours after cell seeding, cells had formed a monolayer, and the medium was changed to Leibovitz’s L-15 medium (Fisher, 11540556) with the corresponding supplements. Subsequently, the ibidi Pump System (ibidi GmbH, 10902) was assembled and connected to the µ-Slide, which was then placed at the insert stage of our epifluorescence microscope. Live cell imaging was conducted as described above, using a 10x Plan Fluor DL objective with NA = 0.13. In the acquired phase contrast images, flow direction was from the bottom to the top of the field of view. In most recordings, and unless otherwise indicated, cells were first kept under stationary conditions (1–3 h), then exposed to constant magnitude fluid shear stress of 10 dynes/cm^2^ for 20 h, and finally to constant magnitude fluid shear stress of 40 dynes/cm^2^ for additional 20 h. Kinematical analysis of cell motion was conducted as described above.

### Traction force and monolayer stress microscopy (TFM and MSM)

Polyacrylamide hydrogels were prepared by first incubating glass-bottom plates with 6 wells (Cellvis, P06-1.5H-N) for 30 min with 500 μL of 0.5 M NaOH^84, 85^. Glass-bottoms were then rinsed with distilled water, and incubated with 500 μL of 2% 3-aminopropyltriethoxysilane (Sigma, A3648) in 95% ethanol for 5 min. Following rinsing with water, 500 μL of 0.5% glutaraldehyde (Sigma-Aldrich, G6257) were added to each well for 30 min. Wells were rinsed with water again and dried at 60 °C. To prepare polyacrylamide hydrogels of 3 kPa, two mixtures were prepared containing 5% acrylamide (Sigma, A4058) and 0.1% bis-acrylamide (Fisher, BP1404-250)^84^, the second of which additionally contained 0.03% of 0.5 μm fluorescent tracer beads (Invitrogen, F8813) for TFM experiments. To initiate polymerization 0.06% ammonium persulfate (Fisher, BP17925) and 0.43% TEMED (Sigma-Aldrich, 110732) were added to the mixtures. First, 3.6 µL of the first mixture without the beads was added at the center of each well, capped with 18-mm untreated circular glass coverslips, and allowed to polymerize for 20 min. After coverslip removal, 3.6 µL of the mixture containing tracer beads was added and sandwiched again with an 18-mm untreated circular glass coverslip and allowed to polymerize for 20 min. After the coverslips were removed, 50 mM HEPES (Roth, HN78.3) at pH 8.5 was added to the wells, and coverslips were removed. Hydrogels were UV-sterilized for 1 h and then activated by adding 500 µL of 0.1% weight/volume heterobifunctional cross-linker Sulfo-SANPAH (ProteoChem, c1111) in 1% dimethyl sulfoxide (DMSO) (Sigma, D2650) and 50 mM HEPES, pH 8.5, on the upper surface of the hydrogels, followed by exposure to UV light for 10 min. Hydrogels were washed with 50 mM HEPES at pH 8.5 and coated with 500 µL of 0.25 mg/ml rat tail collagen I in 50 mM HEPES at pH 8.5 overnight at room temperature. The next morning, the collagen I-coated surfaces were washed with HEPES, and gels were stored in 50 mM HEPES solution.

TFM was performed as previously described^86, 87^. Briefly, in TFM, cells actively pull on the elastic hydrogel containing fluorescent tracer beads depending on how well their focal adhesions are organized and connected to the underlying cytoskeleton. The resulting traction stresses can be analytically calculated from the measured displacements of hydrogel-embedded beads^86, 88^. Prior to seeding the cells, hydrogels were equilibrated with cell medium for 30 min at 37 °C. Cells were then seeded at a specific concentration to yield 100% confluent monolayers 24 h later. Specifically, 24 h prior to live-cell imaging, HMEC-1 and hCMEC/D3 were seeded directly onto the hydrogels at a density of 4 × 10^5^ cells and 3 × 10^5^ cells per well, respectively. Cells were exposed to flow 2–5 h after the start of the microscopy recording acquisitions (as indicated in the corresponding figure legends) and were imaged under these conditions for a period of 20 h. Afterwards, flow was ceased, and cells were imaged for additional 10 h to evaluate their recovery. Multi-channel time-lapse sequences of fluorescence (to image the beads within the upper portion of the hydrogels) and phase contrast images (to image the cells) were acquired using an inverted Nikon Eclipse Ti2 with a Prime 95B sCMOS camera and a 10×0.13NA Plan Fluor air objective. The microscope was controlled by the NIS Elements software. Temperature was maintained at 37 °C throughout the experiment. Images were acquired every 10 min for approximately 30 h. Subsequently, at each time interval, we measured the 2D deformation of the substrate using an image correlation technique similar to particle image velocimetry. We calculated the local deformation vector by performing image correlation between each image and an undeformed reference image, which we acquired by adding 200 µL of 10% w/v sodium dodecyl sulfate (SDS) (Roth, 2326.2) at the end of each recording to detach the cells from the hydrogels. We used interrogation windows of 32 × 16 pixels (window size x overlap). Details on the calculations of the two-dimensional traction stresses that cell monolayers exert on the hydrogel have been described in detail elsewhere^87, 88^.

We calculated the strain energy (${U}_{s}$) as the mechanical work imparted by cells to deform the hydrogel as ${U}_{s}=\frac{1}{2}\,\int {{{\boldsymbol{t}}}}(z=h)\,\bullet {{{\boldsymbol{u}}}}(z=h){ds}$, where ${{{\boldsymbol{u}}}}\left(z=h\right)$ is the measured displacement vector field on the free surface of the hydrogel, ${{{\boldsymbol{t}}}}$ is the calculated traction stress vector field, ${\int }_{s}{ds}$ represents the surface integral, *h* is the height of the hydrogel and *z* the vertical coordinate. For each field of view originating from different wells, we normalized the strain energy value with that of time = 0 min, to avoid discrepancies due to slight differences in cell confluence among wells. To compare the relative increase in strain energy upon flow application of vehicle control-treated cells to cells treated with different pharmacological inhibitors, we averaged the normalized strain energy values one hour prior to flow application and one hour immediately after flow application and calculated their ratio (Supplementary Fig. 6F). To compare the relative increase in strain energy during the 20 h period of flow application of vehicle control-treated cells to cells treated with different pharmacological inhibitors, we averaged the normalized strain energy values one hour immediately after flow application and one hour before flow cessation and calculated their ratio (Supplementary Fig. 6G).

We used Monolayer Stress Microscopy (MSM) to infer the EC monolayer tensile and compressive stresses from the traction stresses EC exert on the hydrogel, as done previously^82, 89^. Monolayer stresses (σ) are calculated from the system of equations composed of the Beltrami differential equation and Newton’s equilibrium equations. This formulation only requires the traction stresses (τ) in both the x and y directions, the cell’s Poisson’s ratio (ν), and the monolayer thickness (h). This model considers the following assumptions: perfect cell-cell adhesions (monolayer is a continuum), perfect cell-substrate adhesions (traction forces are balanced with monolayer stresses), monolayer thickness is uniform and constant (uniform properties), cells are considered linear elastic isotropic materials, traction stresses are generated only in the plane of the monolayer and are negligible in the vertical direction (the normal component of traction forces is negligible), and time-dependent changes in monolayer configuration and strains are small (small strain assumption). Displacements along the edge of the monolayer/image were set to zero while Poisson’s coefficient and monolayer thickness were set to 0.48 and 10 μm, respectively. Finally, we transformed the monolayer stresses from the cartesian coordinate system (x,y) to the principal coordinate system ($I,{II}$). This new system does not consider the shear component of stress and allows for the computation of the maximum monolayer tension (${\sigma }_{I}$) and compression (${\sigma }_{{II}}$)^89^. We used a custom MATLAB (MathWorks) script with a finite element implementation of the mentioned model to calculate the monolayer stresses at each time point. The source code is provided at https://github.com/ebastoun/Monolayer-Stress-Microscopy (DOI: doi.org/10.5281/zenodo.7155523).

In all TFM experiments, cells were exposed to flow for 20 h. Prior to flow exposure, cells were imaged for 4-5 h under static conditions to establish a pre-flow baseline. Following the 20 h flow exposure, the duration of post-flow imaging varied according to the experimental aim. In experiments designed to characterize the persistence of flow-induced changes (Fig. 4 and Supplementary Fig. 4), EC were monitored for up to 10 h after flow cessation. In pharmacological perturbation experiments, which were primarily designed to identify the mechanisms underlying flow-induced rheotaxis and traction changes (Figs. 6 and 7), imaging was continued for an additional 5 h after flow cessation to assess whether the kinematic and dynamic responses, when not abolished by the respective inhibitor, persisted after removal of the flow stimulus as in vehicle control-treated HMEC-1 cells.

### Characterization of traction adhesion dynamics

To quantify how dynamic the active focal adhesions of EC are, we performed cross-correlation analysis on subsequent maps of EC traction stress magnitude. We assumed that less dynamic traction forces would result in successive images with high similarity (stable focal adhesions, high correlation), while highly dynamic traction forces would result in images with low similarity (dynamic focal adhesions, low correlation). The cross-correlation coefficient was calculated between a given frame and the next 20 successive frames (lag). Then, we created a kymographic representation of the magnitude of the correlation coefficient as a function of lag (y-direction) and time (x-direction). The cross-correlation analysis and the kymograph were performed using a custom MATLAB (MathWorks) script.

### Immunofluorescence microscopy, nuclear segmentation, and quantitative analysis of cytoskeletal organization

HMEC-1 residing on glass substrates were incubated with 1 μg/mL Hoechst to stain the cells’ nuclei for 10 min at 37°C prior to fixation. The following steps were carried out at room temperature. Cells were washed with PBS and fixed with 4% EM grade formaldehyde (Thermofisher, 28906) in PBS for 10 min. Following a wash with PBS, samples were permeabilized for 5 min in 0.2% Triton X-100 (Roth, 3051.4) in PBS and washed again with PBS. Samples were then blocked for 30 min with 5% BSA (AppliChem, A6588) in PBS and then incubated for 1 h with primary antibodies (pMLC: Cell signaling technology, 3671, RRID: AB_330248; vinculin: Sigma, V9131-2ML, RRID: AB_477629; TGF-β1, Thermofisher, MA1-169, RRID: AB_2536871) diluted 1:100 in PBS containing 2% BSA. For actin staining, we used 0.2 µM AlexaFluor488 phalloidin (Thermofisher, A12379). Samples were washed three times in PBS and then incubated for 1 h with secondary antibodies diluted 1:250 in PBS containing 2% BSA. We used goat anti-mouse Alex Fluor 647 (Thermo Fisher, A32728, RRID: AB_2633277) and goat anti-rabbit Alex Fluor 546 (Thermo Fisher, A11035, RRID: AB_2534093). Samples were washed three times in PBS and then stored in 2 mL PBS at 4 °C until imaging. For epifluorescence imaging, we used an inverted Nikon Eclipse Ti2 with a Prime 95B sCMOS camera, 40×0.60NA Plan Fluor air or a 20×0.75NA Plan Apo air objective, and NIS-Elements software. For automated nuclear cell segmentation, we used the cellC software run in MATLAB (MathWorks)^90^. For quantification of fluorescence intensity of either vinculin, F-actin or pMLC per cell, a custom-built code in MATLAB (MathWorks) was used.

### RNA isolation and RNA sequencing

#### Sample preparation

HMEC-1 and hCMEC/D3 cells were placed at a concentration of 2 × 10^5^ and 1 × 10^5^ cells/well on 20 mm collagen I-coated glass coverslips of 6-well plates for 48 h until they formed a confluent monolayer. Subsequently, they were exposed for 20 h to flow (3 mL/min, using the impinging flow jet device) or not for the static controls. Cells were then harvested and lysed using the QIAshredder Kit (Qiagen; 79656). We analyzed *N* = 6 samples per condition, originating from three independent experiments, with two independent wells analyzed per condition in each experiment. So we analyzed a total of 24 samples. mRNA was harvested using the RNeasy Plus Micro Kit (Qiagen; 74004) and eluted in 30 μL RNase-free water. A NanoDrop ND-1000 spectrophotometer was used to determine concentration (abs 260) and purity (abs260/abs230) of total RNA samples. Agarose gel electrophoresis was used to check the integrity of total RNA samples (performed by Arraystar Inc.).

#### RNA library preparation and RNA sequencing

The total RNA was depleted of rRNAs using the Arraystar rRNA Removal Kit. We used Illumina kits for the RNA-seq library preparation, which included RNA fragmentation, random hexamer-primed first-strand cDNA synthesis, dUTP-based second-strand cDNA synthesis, end-repairing, A-tailing, adapter ligation, and library PCR amplification. The resulting RNA-seq libraries were qualified using Agilent 2100 Bioanalyzer and quantified using the qPCR absolute quantification method. Sequencing was performed using Illumina NovaSeq 6000 and the High-Output Kit with 2×150 read length. We had an average of 40 million reads per sample. The DNA fragments in well-mixed libraries were denatured with 0.1 M NaOH to generate single-stranded DNA molecules, loaded onto channels of the flow cell at 8 pM concentration, and amplified in situ using TruSeq SR Cluster Kit v3-cBot-HS (#GD-401-3001, Illumina). Sequencing was carried out by running 150 cycles on the Illumina X- ten/NovaSeq according to the manufacturer’s instructions.

#### Sequencing data analysis

The raw sequencing data that passed the Illumina quality filter were used for the following analysis. Trimmed reads (trimmed 5’, 3’-adapter bases) were aligned to the reference genome (GRCh37). Based on alignment statistical analysis (mapping ratio, rRNA/mtRNA content, fragment sequence bias), we determined whether the results were suitable for subsequent data analysis. To examine the sequencing quality, the quality score plot of each sample was plotted. The quality score Q is logarithmically related to the base calling error probability (P): Q = −10log_10_(P). For example, Q30 means the incorrect base-calling probability to be 0.001 or 99.9% base-calling accuracy. After quality control, the fragments were 5’, 3’-adapter trimmed and filtered ≤ 20 bp reads with Cutadapt software. The trimmed reads were aligned to the reference genome with Hisat 2 software. In a typical experiment, it is possible to align 40 to 90% of the fragments to the reference genome. However, this percentage depends on multiple factors, including sample quality, library quality and sequencing quality. Sequencing reads are classified into the following classes: (1) mapped: reads aligned to the reference genome (including mRNA, pre-mRNA, poly-A tailed lncRNA and pri-miRNA); (2) mtRNA and rRNA: fragments aligned to rRNA and mtRNA; and (3) unmapped: reads that are not aligned.

Differentially expressed genes (DEGs) and differentially expressed transcripts were calculated^85^. Novel genes and transcripts were also predicted. The expression level (FPKM value) of known genes and transcripts was calculated using ballgown through transcript abundances estimated with StringTie. The number of identified genes and transcripts per group was calculated based on the mean of FPKM in group ≥ 0.5. Fragments per kilobase of transcript per million mapped reads (FPKM) were calculated with the formula: ${FPKM}=\frac{C\times {10}^{6}}{L\times N}$, where C is the number of fragments that map to a certain gene/ transcript, L is the length of the gene/transcript in Kb and N is the number of fragments that map to all genes/transcripts. Differentially expressed gene and transcript analyses were performed with R package ballgown, and both p- and q-values were calculated. Because the DEG list identified was used for pathway enrichment analysis as discussed below, a p-value cutoff was used. Fold change (cutoff 1.5), *p*-value (≤ 0.05), and FPKM ( ≥ 0.5 mean in one group) were used for filtering differentially expressed genes and transcripts.

### Pathway enrichment analysis

Kyoto Encyclopedia of Genes and Genomes (KEGG) pathway analysis of the whole data set of DEGs was performed using the R package GAGE “Generally Acceptable Gene set Enrichment” (GAGE v.2.22.0) implemented in R. Briefly, default parameter settings were used for comparisons of log-scaled gene set expression (i.e., enrichment) data between different flow conditions (q-value < 0.01). This is standard practice for pathway enrichment pipelines. Gene sets were defined using annotations obtained from GAGE v2.22.0, go.db v3.2.2, and kegg.db v3.2.2. The R package “Pathview” v.1.12.0 and KEGGGraph v1.30.0 were used to visualize gene set expression data in the context of functional pathways.

### Determination of shared DEGs and pathways across cell types in response to flow

Differentially expressed genes (DEGs) and enriched pathways identified for each cell type in response to flow were compiled together with their corresponding fold changes and pathway enrichment scores (Supplementary Data 1). A custom script implemented in R v4.3.3 was then used to identify DEGs and pathways shared across cell types. For shared DEGs, the distribution of fold changes across cell types was visualized using histograms. Similarly, pathway enrichment scores were plotted to compare the magnitude and direction of pathway-level responses across cell types. Data visualization was performed using ggplot2 v3.5.1.

### Assessment of cell proliferation through Ki-67 immunostaining and flow cytometry

To qualitatively assess whether the proliferation rate of HMEC-1 cells changed due to exposure to fluid flow as compared to stationary conditions, we first performed immunostaining for the proliferation marker Ki-67. To that end, cells were fixed directly after flow priming for 10 h, 20 h or not (static condition) using 4% formaldehyde in PBS for 10 min at room temperature. The cells were then permeabilized with 0.2% Triton X-100 in PBS for 5 min and washed with PBS. Subsequently, samples were blocked with 5% BSA in PBS for 30 min and incubated with the primary anti-Ki-67 rabbit antibody (Invitrogen, MA514520, RRID: AB_10979488) at a 1:100 dilution in 2% BSA in PBS for 1 h. The samples were washed three times with PBS and blocked again with 5% BSA for 30 min. Finally, the cells were incubated with a goat anti-rabbit-488 antibody (1:250) (Thermofisher, A-11008, RRID: AB_143165) and DAPI (1:1000) for 45 min. After washing three times with PBS, the samples were stored in PBS and imaged using an inverted Nikon Eclipse Ti2 epifluorescence microscope with a Prime 95B sCMOS camera, a 40×0.60NA Plan Fluor air objective, and the NIS Elements software. For quantitative assessment, nuclear regions of interest (ROIs) were generated in Fiji (ImageJ) based on DAPI staining. After background correction, z-stacks were maximum intensity projected, and mean nuclear Ki-67 fluorescence intensity was measured for each segmented nucleus. To define Ki-67 positivity, mean nuclear Ki-67 intensity values from pooled static samples were used to calculate the 80th percentile, resulting in a fixed threshold. The percentage of Ki-67-positive nuclei per image was calculated as the ratio of nuclei with a mean Ki-67 intensity above a fixed threshold, set to distinguish positive staining from background, to the total number of nuclei. This threshold was applied consistently across all conditions.

Flow cytometry analysis on whole-cell samples primed with flow for 20 h or not (static condition) was also performed in sextuplicate. To that end, HMEC-1 cells were first washed with PBS and then incubated with 0.25% trypsin-EDTA (Thermofisher; 25200056) for 15 min to detach cells from the glass-bottom dishes. Trypsin was neutralized with the addition of MCDB medium containing FBS, and cell suspensions were pipetted up and down six times to disperse cell clumps. Cells were strained into 5 mL polystyrene test tubes through a cell strainer snap cap with a 35 μm nylon mesh (Fisher Scientific; 352235), and flow cytometry analysis was performed on a BD LSRFortessa X-20 in high acquisition mode with a flow rate of 60 μL/min. To obtain a pure cell population (and not debris), events were gated based on the SSC vs FSC scatter plot, with a total of 10,000 events recorded per sample. The population was plotted using FSC against time, and the cell count passing through the flow cell within 10 s was determined using the FlowJo software (BD Life Sciences). Cell density was calculated for each sample assuming a flow rate of 60 μL/min. The values were normalized to the mean of the static samples for each experiment.

### Statistics and reproducibility

Statistical analyses were performed with GraphPad Prism 10 (RRID: SCR_002798). Differences in data distributions were determined to be statistically significant when *p* < 0.05: *, *p* < 0.01: **, *p* < 0.001: *** and *p* < 0.0001: **** by running a non-parametric Wilcoxon rank-sum test (WRST, denoted as such in the corresponding figure legends) or, in case of multiple comparisons, a Kruskal-Wallis test with Dunn’s multiple comparison test (KWTD, denoted as such in the corresponding figure legends), as indicated. Statistical parameters are reported in the figure legends. Experiments shown in main figures were performed at least three independent times. The total numbers of images or videomicroscopy recordings analyzed are specified in the corresponding figure legends, together with whether these originated from independent wells. For bulk RNA sequencing, we analyzed N = 6 samples per condition across three independent experiments, with two independent wells per condition in each experiment. Bar plots with error bars show mean and standard deviation (SD) or standard error of the mean (SEM), circles represent mean values of independent microscopy recordings for the corresponding time intervals of each bin (e.g., see Fig. 4C).

## Supplementary information

Transparent Peer Review file

Supplementary Information

Supplementary data 1

Supplementary data 2

Supplementary Video 1

Supplementary Video 2

Supplementary Video 3

Supplementary Video 4

Supplementary Video 5

Supplementary Video 6

## Data Availability

The RNA sequencing data (FASTq files) generated during this study and subsequent analysis are available at the Gene Expression Omnibus (GEO) database (Accession ID: GSE206571). All differential expression analysis results of this study are included as Supplementary Data 1. All source data, i.e., the numerical values underlying the graphs and charts presented in the main figures, are provided as Supplementary Data 2. Each sheet of the table corresponds to a single figure panel and is named accordingly (e.g., Fig. 1E). Due to their large file size, the raw microscopy images (.tif or .nd2 files) generated in this study were not deposited in a public repository but are available from the lead contact upon reasonable request. Any additional information required to reanalyze the data reported in this paper is available from the lead contact upon request.

## Code availability

Codes generated and used in this study are publicly available as described below: (1) Cell kinematics analysis (DOI: doi.org/10.5281/zenodo.7155531); (2) Construction of kymographs and additional analysis based on those (DOI: doi.org/10.5281/zenodo.19386424); and (3) Monolayer stress microscopy (DOI: doi.org/10.5281/zenodo.7155523).
