# Supplementary material for: FAK- and TGF-β1-dependent biomechanical alterations in microvascular endothelial cells exposed to fluid shear stress gradients
Source: Commun Biol. 2026 Nov 3;9:1451. doi: 10.1038/s42003-026-11283-9 (PMC13731646; doi:10.1038/s42003-026-11283-9)
Supplement: Supplementary file 1 [file 42003_2026_11283_MOESM1_ESM.pdf]

nature portfolio

Peer Review File

FAK- and TGF-β1-dependent biomechanical alterations in microvascular endothelial cells exposed to fluid shear stress gradients

Corresponding Author: Professor Effie Bastounis

This file contains all reviewer reports in order by version, followed by all author rebuttals in order by version.

Version 0:

Reviewer comments:

Reviewer #1

(Remarks to the Author)
The manuscript by Rendon et al. employs a previously developed impinging flow device to study the effect of shear stress gradients on migration speeds, coordinated motion and rheotaxis behavior of dermal and brain-derived endothelial cells (ECs). It was determined that ECs traction forces increase upon flow onset and these biomechanical adaptations persist even after flow cessation suggesting that ECs retain mechanical memory of prior flow exposure. Moreover, the effect of inhibition of FAK, ROCK and TGF-B on ECs behavior was also investigated. Overall, I recommend minor revisions before the manuscript is considered for publication. Comments below for the authors' consideration.
1. On page 5, authors mention "...with a progressive decrease in migration speed over time..." It is suggested that the authors show the time evolution of number of cell nuclei positive for Ki-67 in static condition to further substantiate this claim.
2. On page 5, the spelling of "Supplementary" should be corrected everywhere.
3. Figure 1E shows that at t = 0, for flow case, the median of PIV data for cell migration speed is two-fold less than t = 0 for static case. This suggests that fluid flow has an instantaneous effect on cell migration speed. Similarly for coordinated motion. This is surprising as studies on the effect of fluid flow on cell elongation suggest that it takes several hours for cells to morphologically respond to a flow field. It is suggested that the authors comment on this instantaneous change.
4. On page 6, authors mention "...with HMEC-1 responding faster and showing stronger rheotaxis than hCMEC/D3..." It is suggested that the authors include quantification of the rheotaxis strength and timescales in this sentence.
5. In Figure 2, panel A, on the rightmost figure, the authors should correct the spelling of "mean" in the chart title.
6. Supplementary Figure 2B shows representative phase contrast images at different x locations with x = 7.6 mm as the largest x location. Figure 1A (and all other main figures) shows 2 mm as the largest radial location. It is not clear why Supplementary Figure 2B alone incorporates data at the well edge. Moreover, the x location of fluidic outlet is not specified. It is suggested that the authors specify the location of the fluidic outlet.
7. According to Supplementary Figure 2A, ring 1 (having the maximum shear stress gradient) does not show reverse "strong" rheotaxis. Moreover, ring 2 and ring 4 both have negative SSG and similar values of SSG. Both these rings also show similar profiles for the mean radial displacement with time. Could it be that negative values of shear stress gradients are necessary to induce reverse rheotaxis? It is suggested that the authors comment on these observations.
8. The static migration speeds for HMEC-1 in Figure 3C, hCMEC/D3 for 5-25 h differs from the static migration speeds of these cells in Figure 1E. It is suggested that the authors clarify this.
9. On page 7, it is suggested that the authors define TFM at its first occurrence.
10. In Figure 4, the authors utilized the displacement of 0.5 um beads in hydrogels to quantify traction stresses. It is suggested that the authors comment on whether they performed acellular experiments to check if the shear stresses due to the flow field itself has any effect on the displacement of these beads.
11. The authors should correct the spelling of "energy" in the y axis title of Figure 4C.
12. In the first and last paragraph of section titled "FAK inhibition...slows migration", it is suggested that the authors mention that PF-537228 also reduced migration speeds, similar to Y-27632 as shown in Supplementary Figure 6C.
13. On page 10, in the sentence "...and was accompanied by a decrease in cell migration speed (Figure 6C)", the authors should refer to Supplementary Figure 6C instead of Figure 6C.
14. On page 12, the authors should remove "past" from the last line.

Reviewer #2

(Remarks to the Author)
I co-reviewed this manuscript with one of the reviewers who provided the listed reports. This is part of the Communications Biology initiative to facilitate training in peer review and to provide appropriate recognition for Early Career Researchers who co-review manuscripts.

Reviewer #3

(Remarks to the Author)
The authors use an impinging-flow device to examine how spatial shear stress gradients (SSG) microvascular endothelial cells (µEC). Working with two immortalized EC lines from distinct vascular beds (HMEC-1 and hCMEC/D3), they report that flow-driven SSG produce an immediate, sustained reduction in migration speed, enhanced coordination of collective motion, and reverse rheotaxis. These kinematic changes are accompanied by an acute and then quasi-linear increase in traction stresses and monolayer stresses that persists after flow cessation. Combining tfm, msm, RNA sequencing, and pharmacological perturbation, the authors propose that FAK-dependent focal-adhesion dynamics govern rheotactic directionality, whereas TGF-β1 signaling and actomyosin contractility drive the progressive strengthening of traction, and that these two axes are mechanistically separable.

Overall, this reviewer finds the work proposed here to be a valuable study. The focus on microvascular EC under spatial SSG sheds light on a less studied vascular bed, which is needed. Furthermore, few laboratories can observe cells under sustained fluid flow long-term using time lapse microscopy and get the quality of results presented here. However, there are major and minor concerns that need to be addressed as follows:

Major Concerns

1. Near the stagnation point, flow velocity approaches zero and secreted autocrine factors accumulate, whereas at the periphery they are cleared. Taken into account that the authors' proposed underlying mechanism implicates a secreted, latent factor (TGF-β1), there most likely exist a unique spatial distribution of TGF-β1 itself and TGF-β1 concentration is most likely graded or non-uniform as well. However, in the ibidi chamber the spatial distribution of any secreted specifies would be uniform. This leads this reviewer to suspect that the reverse rheotaxis could in part be due to some type of chemotactic response as well. Addressing this point in some way such as by a CFD scalar-transport simulation or additional experiments to remove the confounding factor of a chemical species gradient would help strengthen the claims made here.

2. The current metrics supporting the notion of flow leading to TGF-β1 activation are transcriptomic, and assembled from downstream and parallel readouts. While evidence of transcription is great, transcription does not always lead to translation as the cell has multiple mechanisms that could lead to the decoupling of transcription and translation. With that being said, direct evidence showing that flow itself activates TGF-β1 signaling at the protein level is not demonstrated here. A demonstration of changes in protein levels via immunoblots or addressing this point in some way in the manuscript is critical if the authors want to make this claim.

3. Each of the pharmacological inhibitors used rests on a single molecule whose selectivity profile and in some cases off target effects should be acknowledged, with appropriate citations. Y-27632, beyond ROCK1/2, inhibits the PKC-related kinase PRK2/PKN (IC50 ≈ 600 nM) and, at higher concentrations, PKC and PKA (Ishizaki et al., Mol Pharmacol 2000, 57:976; independent selectivity profiling in Davies et al., Biochem J 2000, 351:95). PF-573228 is, to its credit is roughly 50–250-fold selective for FAK (IC50 ≈ 4 nM) over Pyk2 and kinases such as CDK1/7 and GSK-3β (Slack-Davis et al., J Biol Chem 2007, 282:14845) so its liability is residual Pyk2/off-kinase activity at higher doses rather than frank dual inhibition; the authors could cite this selectivity as a strength of their choice. SB-525334, though ALK5-selective, is only about 4-fold more potent against ALK5 (IC50 ≈ 14 nM) than against the closely related activin receptor ALK4 (IC50 ≈ 59 nM), while being inactive against ALK2/3/6 (Grygielko et al., J Pharmacol Exp Ther 2005, 313:943); at the 10 µM working dose used here it therefore acts as an effective ALK5/ALK4 (TGF-β/activin) dual inhibitor. Since activin/nodal signaling also converges on SMAD2/3, the SB-525334 result does not cleanly isolate the TGF-β type I receptor, and this point should be acknowledged in the manuscript where appropriate.

4. As a follow-up to point 3, a second FAK inhibitor or FAK/PTK2 knockdown and/or where feasible, orthogonal confirmation for the TGF-β portion would substantially harden the mechanistic conclusions.

5. The active-focal-adhesion-turnover data complicate the framing that FAK specifically governs adhesion turnover: by the authors' own description, Y-27632 (ROCK) reduces turnover most strongly, with PF-573228 having a lesser effect on the same readout. The discussion should reconcile this, since it bears on how cleanly the FAK and ROCK contributions can be separated.

6. The directional flip is only shown as representative kymographs and does not appear to be accompanied by any explicit statistical testing contrasting forward versus reverse migration for any of the directional data. Proving statistical significance would help definitively formalize the "true polarity reversal" claim made here.

7. HMEC-1 and hCMEC/D3 are both immortalized lines that carry well-documented junctional and barrier abnormalities, which is relevant given that monolayer stress microscopy is framed as a readout of barrier-integrity strength. This point could be improved by verifying that these cell lines express the expected traditional barrier molecules, utilizing primary

microvascular endothelial cells, or at the bare minimum acknowledging this point in the manuscript as a limitation.

8. A major issue exist regarding monolayer stress microscopy calculations. As acknowledged by the authors, MSM sets displacements to zero at the image boundary, but only a small portion of the monolayer boundary can be seen and the rest of the monolayer most likely extends well beyond the field of view shown here. Therefore, a zero-displacement boundary condition at regions where the monolayer boundary extends beyond the field of view is incorrect as this is physically impossible. Furthermore, such assumptions will bias inferred intercellular stresses, and this effect most likely will be amplified in a radially graded field. This is an inherent limitation of MSM when done with monolayers that expand beyond the field of view, which is enhanced here. This point needs to be addressed explicitly in the manuscript as this is a significant limitation and source of error for the msm calculations.

9. The authors should consider adding qualifiers or reframing their claim of “mechanical memory” as this claim appears to be somewhat partially weakened within the paper. For example, Supp. Fig. 3 notes only “minor recovery” for hCMEC/D3, and Supplementary Fig. 4B shows hCMEC/D3 strain energy post-flow returning to a level not significantly different from static controls, whereas HMEC-1 traction remains elevated. If this truly is a conserved behavior this reviewer would expect comparable recovery levels regardless of cell type, but this is not the case. “Mechanical memory” appears to potentially be cell-type-specific, and not a general behavior. This is in addition to the point mentioned above that both cell lines are immortalized as well. This point needs to be addressed in the manuscript.

Minor Concerns

10. Supplementary Fig. 4 is titled and structured for hCMEC/D3, yet panel 4C presents HMEC-1 monolayer-stress data (explicitly referencing main Fig. 4A). Either the label is wrong or the panel is misplaced; in either case the HMEC-1 monolayer-stress quantification that supports main Fig. 4 should not be buried in the hCMEC/D3 figure without a clear cross-reference.

11. Throughout the main manuscript and supplementary document the FAK inhibitor is referred to as “PF-573228,” “PF537228,” and “PF573228”. Please use one consistent name to refer to this inhibitor.

12. Multiple typos exits and extensive proofreading is needed. For example,“Supplementarymentary,” – line 27, line 132, “progression.ostrowski” – line 64, RNA “integrality”, “mapp.i.ng,”, and “Signs-Aldrich,”. However, there are too many to put them all and more exist.

13. Post-flow observation is 10 h in Figs. 3–4, but 5 h in Figs. 6–7; this matters for cross-figure comparison of the persistence claim and should be reconciled or explained.

14. Although line, plots of shear stress and SSG are provided, figures of the spatial shear stress gradient (or color plots) are not provided. This is important as it would provide a visual of where the SSG are in relation to the cells shown in the phase images and in relation to migration, traction data, etc.

15. As a follow up to point 14, It also is not clear where the cells, ECM displacement data, traction data, etc. are physically located in relation to the radial distance. Looking at the plots provided the shear stress seems to approach zero at a radial distance of < 1mm (although the chamber itself has a max radial distance of 2 mm). With that being said, some kind of schematic or illustration showing where the cells are located would be helpful. In other words, something showing what location in the chamber the cells are located and where this data subsequently corresponds to would be helpful.

Version 1:

Reviewer comments:

Reviewer #1

(Remarks to the Author)
We thank the authors for addressing all our comments. We now recommend accepting the manuscript.

Reviewer #2

(Remarks to the Author)
I co-reviewed this manuscript with one of the reviewers who provided the listed reports. This is part of the Communications Biology initiative to facilitate training in peer review and to provide appropriate recognition for Early Career Researchers who co-review manuscripts.

Reviewer #3

(Remarks to the Author)
All comments have been sufficiently addressed and I have no additional comments.

Open Access This Peer Review File is licensed under a Creative Commons Attribution 4.0 International License, which permits use, sharing, adaptation, distribution and reproduction in any medium or format, as long as you give appropriate credit to the original author(s) and the source, provide a link to the Creative Commons license, and indicate if changes were made.
In cases where reviewers are anonymous, credit should be given to 'Anonymous Referee' and the source.
The images or other third party material in this Peer Review File are included in the article's Creative Commons license, unless indicated otherwise in a credit line to the material. If material is not included in the article's Creative Commons license and your intended use is not permitted by statutory regulation or exceeds the permitted use, you will need to obtain permission directly from the copyright holder.
To view a copy of this license, visit https://creativecommons.org/licenses/by/4.0/

We thank the editorial team and Reviewers for comments and suggestions on our manuscript entitled “FAK- and TGF-β1-dependent biomechanical alterations in microvascular endothelial cells exposed to spatial fluid shear stress gradients" (Manuscript No. COMMSBIO-26-4892-A). We have carefully modified and revised the manuscript by addressing concerns raised by both the editorial team (see online Table) and the Reviewers.

In particular, we performed several new experiments to strengthen the mechanistic conclusions of our study. Specifically, (1) we further validated the roles of FAK and TGF-β1 signaling in rheotaxis by performing additional experiments using a second FAK inhibitor (FAK-14) and an independent TGFβR-1 receptor inhibitor (LY-364947), respectively. (3) We examined whether the flow-induced increase in TGF-β1 mRNA translates into increased protein expression by performing immunostaining of HMEC-1 cell monolayers exposed to flow versus static conditions. Finally, (3) we confirmed that fluid flow does not artifactually displace the fluorescent beads embedded on the hydrogels used for traction force microscopy by performing control experiments on cell-free 3 kPa hydrogels exposed to the same flow conditions (3 mL/min). These experiments demonstrate that the measured bead displacements arise exclusively from cell-generated forces and not from fluid flow itself.

These additional experiments substantially strengthen the conclusions of the manuscript and further support the proposed mechanistic model. Below, please find a point-by-point response to Reviewers' comments. The comments are in *blue italics,* and our responses are in black. Note that revisions made according to the requests of the editorial team are outlined in the right-hand column of the online table together with relevant notes.

Reviewer #1:

The manuscript by Rendon et al. employs a previously developed impinging flow device to study the effect of shear stress gradients on migration speeds, coordinated motion and rheotaxis behavior of dermal and brain-derived endothelial cells (ECs). It was determined that ECs traction forces increase upon flow onset and these biomechanical adaptations persist even after flow cessation suggesting that ECs retain mechanical memory of prior flow exposure. Moreover, the effect of inhibition of FAK, ROCK and TGF-B on ECs behavior was also investigated. Overall, I recommend minor revisions before the manuscript is considered for publication. Comments below for the authors' consideration.

We thank the Reviewer for appreciating our work and its novelty. Below, we indicated how we addressed one by one the minor issues raised.

Minor points

1. On page 5, authors mention "...with a progressive decrease in migration speed over time..." It is suggested that the authors show the time evolution of number of cell nuclei positive for Ki-67 in static condition to further substantiate this claim.

In revised Supplmentary Fig. 1C-D we now include quantification of Ki-67-positive nuclei at both 10 h and 20 h after flow exposure, rather than only at a single time point (previously at 10 h post-flow exposure). For each time point, we compare Ki-67 fluorescence intensity of cells exposed to flow relative to static controls. These data clearly support our conclusion that flow exposure reduces cell proliferation relative to static controls.

2. On page 5, the spelling of "Supplementary" should be corrected everywhere.

The spelling has been corrected everywhere accordingly.

3. Figure 1E shows that at t = 0, for flow case, the median of PIV data for cell migration speed is two-fold less than t = 0 for static case. This suggests that fluid flow has an instantaneous effect on cell migration speed. Similarly for coordinated motion. This is surprising as studies on the effect of fluid flow on cell elongation suggest that it takes several hours for cells to morphologically respond to a flow field. It is suggested that the authors comment on this instantaneous change.

The reviewers brings up an interesting point, which is now explicitily discussed in the revised discussion section (p. 13, lines 27-31). In this study, we discovered that the kinematics (i.e., migration speed and directionality of motion) as well as dynamics (i.e., traction forces) of µEC change immediately upon flow exposure. Yet this does not mean that cell shape changes occur instantaneously. That is something we did not characterize herein, and definitely something we did not observe. Cell shape changes occur at longer time scales, as has been described in other works of cells exposed to constant shear stresses (Remuzzi et al., 1984) or gradients (Ostrowski et al., 2014).

4. On page 6, authors mention "...with HMEC-1 responding faster and showing stronger rheotaxis than hCMEC/D3..." It is suggested that the authors include quantification of the rheotaxis strength and timescales in this sentence.

We have now included boxplots in revised Fig. 2D-E to better quantify differences in rheotactic behavior of the two cell types over time. HMEC-1 respond within the first 5 h to flow, while it takes longer for hCMEC/d3 to respond, and their rheotactic response is less strong. These findings are now presented in the corresponding results section (p. 6, lines 12-15).

5. In Figure 2, panel A, on the rightmost figure, the authors should correct the spelling of "mean" in the chart title.

The spelling has been corrected accordingly.

6. Supplementary Figure 2B shows representative phase contrast images at different x locations with x = 7.6 mm as the largest x location. Figure 1A (and all other main figures) shows 2 mm as the largest radial location. It is not clear why Supplementary Figure 2B alone incorporates data at the well edge. Moreover, the x location of fluidic outlet is not specified. It is suggested that the authors specify the location of the fluidic outlet.

We thank the Reviewer for this comment. The purpose of Supplementary Fig. 2B is different from that of the main figures (e.g., Fig. 1). While the main figures focus on the region directly influenced by the impinging jet, where spatial fluid shear stress gradients are substantial (up to a radial distance of approximately 2 mm), the experiments in Supplementary Fig. 2B were performed to determine whether the reduction in cell numbers under flow (a assessed via flow cytometry and shown in Supplementary Fig. 1B) was spatially restricted or extended across the entire area occupied by the cells. For this analysis, we acquired images over a much larger region of the well, extending to a radial distance of 7.6 mm. Although shear stresses beyond the central region are very low (approximately 0.05 Pa), they are not zero. We have now clarified these points in revised legends of Fig. 1B-C and Supplementary Fig. 2B, and also explain the different spatial ranges shown in the main figures versus Supplementary Fig. 2B. In addition, as suggested by the Reviewer, we now indicate the location of the fluidic outlet in Supplementary Fig. 2B.

7. According to Supplementary Figure 2A, ring 1 (having the maximum shear stress gradient) does not show reverse "strong" rheotaxis. Moreover, ring 2 and ring 4 both have negative SSG and similar values of SSG. Both these rings also show similar profiles for the mean radial

displacement with time. Could it be that negative values of shear stress gradients are necessary to induce reverse rheotaxis? It is suggested that the authors comment on these observations.

The reviewer brings up a very interesting topic. The behavior of Ring 1 is difficult to interpret because cells located immediately beneath the impinging jet are already positioned at the center of the flow field and therefore have very limited capacity for further inward migration. Consequently, the displacement measured in this region cannot be directly compared with that in the surrounding rings. We also agree that Rings 2 and 4, which both experience negative SSG, exhibit similar negative radial displacements. While this observation raises the possibility that negative SSG may contribute to reverse rheotaxis, our current experimental configuration does not allow us to isolate the effect of the sign of the SSG explicitily. Addressing this question would require microfluidic devices capable of independently controlling the sign (and magnitude) of the SSG while remaining compatible with long-term live-cell imaging (Dolan et al., 2013). We have now included this discussion in the revised manuscript (p. 14, lines 1-4).

8. The static migration speeds for HMEC-1 in Figure 3C, hCMEC/D3 for 5-25 h differs from the static migration speeds of these cells in Figure 1E. It is suggested that the authors clarify this.

We thank the Reviewer for pointing out this error. In the original Fig. 3C, the migration speeds of HMEC-1 cells cultured on 3 kPa hydrogels were inadvertently plotted instead of the migration speeds of HMEC-1 cells on glass, which are shown throughout the remainder of the figure. Because HMEC-1 cells migrate significantly more slowly on 3 kPa hydrogels than on glass, this resulted in the observed discrepancy. We have corrected this error in the revised Fig. 3C by replacing the incorrect dataset with the corresponding migration speeds measured on glass. In addition, we now discuss the differences in the migratory behavior of cells cultured on glass versus 3 kPa hydrogels in the revised discussion (p. 16, lines 8-15).

9. On page 7, it is suggested that the authors define TFM at its first occurrence.

In the revised manuscript, we do now define TFM at its first occurrence (p. 7, line 20).

10. In Figure 4, the authors utilized the displacement of 0.5 um beads in hydrogels to quantify traction stresses. It is suggested that the authors comment on whether they performed acellular experiments to check if the shear stresses due to the flow field itself has any effect on the displacement of these beads.

To address this concern, we performed acellular control experiments in which flow was applied to 3 kPa polyacrylamide hydrogels embedded with 0.5 µm fluorescent beads, in the absence of cells. We found that flow did not induce measurable bead displacements (revised Supplemental Video 1, part B and revised results section p. 7, lines 22-25). The very small displacements detected by PIV were comparable before and after flow initiation and are therefore attributable to measurement noise rather than fluid-induced substrate deformation. To further document these control experiments, we have included Supplementary Video 1 (revised Supplemental Video 1, part B) in the revised manuscript.

11. The authors should correct the spelling of "energy" in the y axis title of Figure 4C.

We have corrected the spelling of "energy" in the revised figure.

12. In the first and last paragraph of section titled "FAK inhibition...slows migration", it is suggested that the authors mention that PF-537228 also reduced migration speeds, similar to Y-27632 as shown in Supplementary Figure 6C.

Per Reviewer's recommendation in the revised results section, we now state: "ROCK inhibition also reduced the mean migration speed by approximately 50%, similar to FAK inhibition (Supplementary Fig. 6C). However, unlike FAK-inhibited cells, ROCK-inhibited cells retained the ability to migrate against the direction of flow, albeit at a reduced speed (Fig. 6D and Supplementary Fig. 6B,C)."

13. On page 10, in the sentence "...and was accompanied by a decrease in cell migration speed (Figure 6C)", the authors should refer to Supplementary Figure 6C instead of Figure 6C.

We corrected the mistake accordingly.

14. On page 12, the authors should remove "past" from the last line.

We removed the word past.

Reviewer #3

The authors use an impinging-flow device to examine how spatial shear stress gradients (SSG) microvascular endothelial cells (µEC). Working with two immortalized EC lines from distinct vascular beds (HMEC-1 and hCMEC/D3), they report that flow-driven SSG produce an immediate, sustained reduction in migration speed, enhanced coordination of collective motion, and reverse rheotaxis. These kinematic changes are accompanied by an acute and then quasi-linear increase in traction stresses and monolayer stresses that persists after flow cessation. Combining tfm, msm, RNA sequencing, and pharmacological perturbation, the authors propose that FAK-dependent focal-adhesion dynamics govern rheotactic directionality, whereas TGF-β1 signaling and actomyosin contractility drive the progressive strengthening of traction, and that these two axes are mechanistically separable. Overall, this reviewer finds the work proposed here to be a valuable study. The focus on microvascular EC under spatial SSG sheds light on a less studied vascular bed, which is needed. Furthermore, few laboratories can observe cells under sustained fluid flow long-term using time lapse microscopy and get the quality of results presented here. However, there are major and minor concerns that need to be addressed as follows:

We thank the Reviewer for appreciating the novelty and quality of our work. Below, we addressed the major and minor comments raised.

Major comments

1. Near the stagnation point, flow velocity approaches zero and secreted autocrine factors accumulate, whereas at the periphery they are cleared. Taken into account that the authors' proposed underlying mechanism implicates a secreted, latent factor (TGF-β1), there most likely exist a unique spatial distribution of TGF-β1 itself and TGF-β1 concentration is most likely graded or non-uniform as well. However, in the ibidi chamber the spatial distribution of any secreted specifies would be uniform. This leads this reviewer to suspect that the reverse rheotaxis could in part be due to some type of chemotactic response as well. Addressing this point in some way such as by a CFD scalar-transport simulation or additional experiments to remove the confounding factor of a chemical species gradient would help strengthen the claims made here.

We thank the Reviewer for raising this important point. We agree that, in principle, the impinging-flow geometry could generate spatially non-uniform distributions of secreted autocrine factors through the interplay of secretion, convection, and diffusion. At present, however, we do not directly measure the spatiotemporal distribution of endogenous TGF-β1 and therefore cannot determine whether a soluble TGF-β1 gradient forms under our experimental conditions.

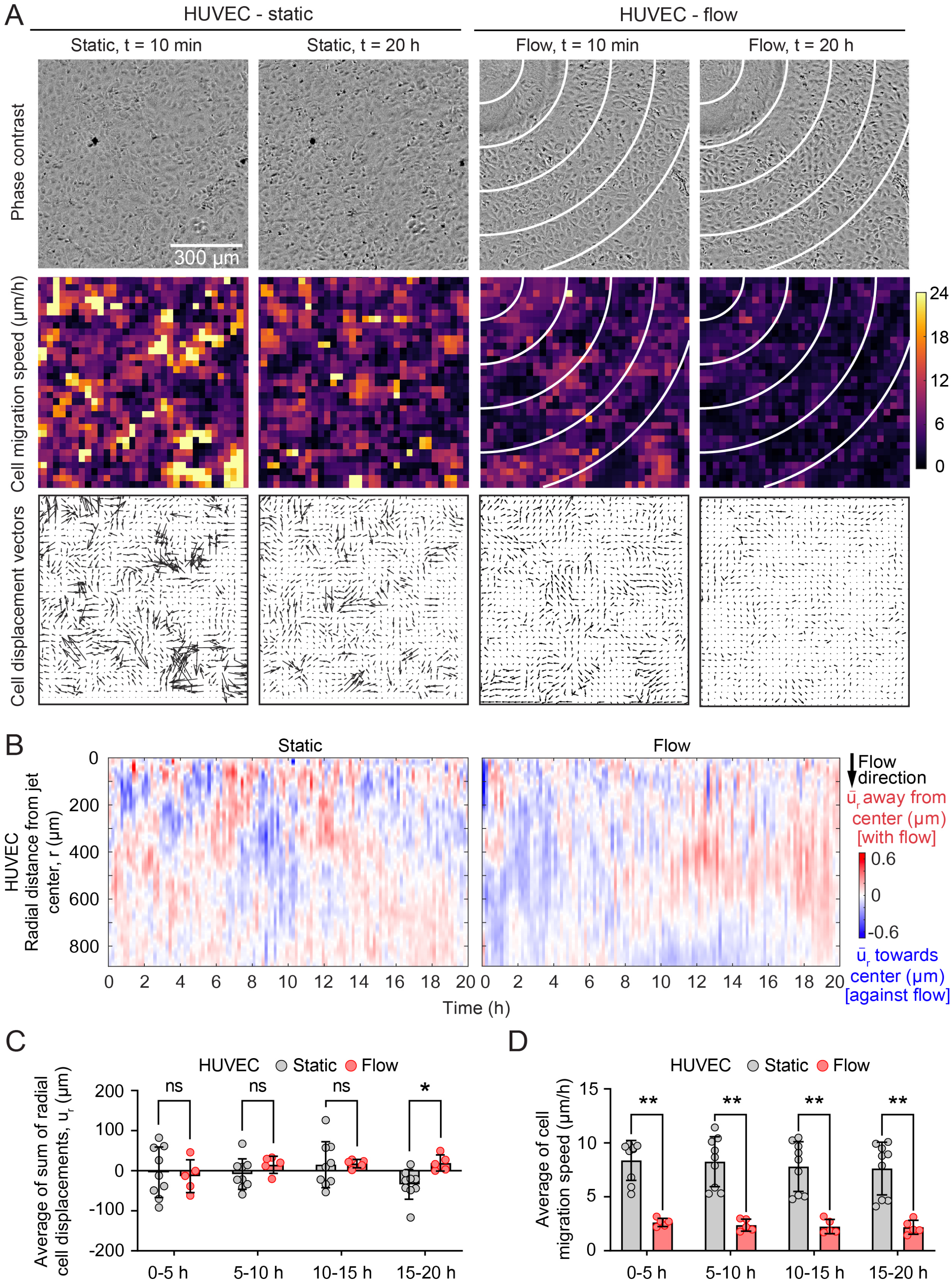

A
HUVEC - static
HUVEC - flow
Static, t = 10 min
Static, t = 20 h
Flow, t = 10 min
Flow, t = 20 h
Phase contrast
300 µm
Cell migration speed (µm/h)
24
18
12
6
0
Cell displacement vectors
B
Static
Flow
HUVEC
Radial distance from jet center, r (µm)
Time (h)
Flow direction
ū_r away from center (µm) [with flow]
0.6
0
-0.6
ū_r towards center (µm) [against flow]
C
HUVEC Static Flow
Average of sum of radial cell displacements, u_r (µm)
ns
ns
ns
*
0-5 h
5-10 h
10-15 h
15-20 h
D
HUVEC Static Flow
Average of cell migration speed (µm/h)
**
0-5 h
5-10 h
10-15 h
15-20 h

Figure R1: A Representative phase contrast images, cell migration speed (µm/h), and cell displacement vectors (calculated from consecutive frames acquired 10 min apart) for HUVEC in monolayer at t=10 min and t=20 h under static (columns 1, 2) or flow exposed conditions (columns 3, 4). Jet center is positioned at the center of the field of view imaged, but only a quarter of the field of view is shown. White or black contours mark concentric regions spaced 185 µm apart from the jet orifice. Cell displacements are scaled 15x and 30x larger for static and flow-exposed cells, respectively. Scale bar: 300 µm. **B** Kymograph of the mean radial cell displacements, u_r (in µm, see colorbar) for HUVEC in monolayer under static (left) or flow (right) conditions. The center of the polar coordinate system is at the center of the field of view (where the orifice of the jet is for flow exposed cells). Kymographs are representative of n=10 recordings on average from at least N=3 independent experiments. **C** Bar plots of the integral of radial HUVEC displacements, u_r (µm), over radial distance, averaged over the indicated time periods, for HUVEC exposed to flow for 20 h or maintained under static conditions. Data were binned according to the experimental recording phase: 0–5 h, 5–10 h, 10–15 h, and 15–20 h. Flow was imposed as soon as the recordings were initiated. Superimposed circles refer to average values of independent recordings. An average of n=7 recordings from at least N=3 independent experiments was analyzed. Mean±SD. WRST, $p>0.05$: non significant, ns; $p<0.05$: *. **D** Same as in panel C but bar plots are showing average cell migration speed (µm/h) for HUVEC exposed to flow for 20 h or maintained under static conditions. WRST, $p<0.01$: **.

Nevertheless, several observations argue against a model in which a soluble TGF-β1 gradient is sufficient to explain reverse rheotaxis. First, macrovascular HUVEC upregulate TGF-β1 in response to flow but do not exhibit reverse rheotaxis under the same experimental conditions (Fig. R1; RNA-seq data are available under Accession ID GSE206571, access token: ahsraiwmltarfkp). We decided not to include these HUVEC data in the revised manuscript because the study focuses primarily on the responses of microvascular endothelial cells, and a systematic comparison of the molecular and mechanical differences between macrovascular and microvascular endothelial cells falls beyond the scope of the present work. More importantly, our functional perturbation experiments indicate that TGF-β1 signaling contributes to the mechanical response to flow rather than acting as the directional guidance cue. Recombinant TGF-β1 increased endothelial traction stresses under static conditions, while pharmacological inhibition of TGF-β1 signaling prevented the flow-induced increase in traction forces. However, inhibition of TGF-β1 signaling did not alter reverse rheotaxis, a finding that we now confirm using an additional pharmacological inhibitor (revised Supplementary Fig. 8). Together, these results indicate that TGF-β1 signaling regulates flow-induced traction generation but is not sufficient to determine migration direction.

Because TGF-β1 is commonly secreted as a latent complex that can become tethered to the extracellular matrix rather than remaining freely soluble (Hinz, 2015), we further examined its localization by immunostaining permeabilized HMEC-1 cells under static and flow conditions. Despite the increased *TGFB1* transcript levels detected by RNA sequencing, total TGF-β1 fluorescence intensity was comparable between flow-exposed and static cells (revised

Supplementary Fig. 5E). This apparent discrepancy is not unexpected, as transcriptional upregulation of *TGFB1* does not necessarily translate into an increase in total cellular TGF-β1 protein, particularly given the additional regulation of TGF-β1 through secretion, extracellular sequestration, and activation. Indeed, previous studies have reported increased *TGFB1* mRNA in endothelial cells exposed to shear stress, along with changes in the biologically active fraction of TGF-β1 (Ohno et al., 1995, Egorova et al., 2011).

Interestingly, flow-exposed cells exhibited a more striated pattern of TGF-β1 staining, with prominent pericellular localization. Additional spinning-disk confocal microscopy suggested that part of this signal may be associated with the extracellular matrix (data not shown). However, because our immunostaining does not specifically distinguish latent, matrix-bound, and active TGF-β1, we refrain from assigning this localization to a particular molecular pool. Importantly, our conclusion that TGF-β1 signaling is enhanced by flow does not rely solely on the increase in TGFB1 transcript abundance. Rather, pathway enrichment analysis of the RNA sequencing data identified broader upregulation of the TGF-β1 signaling pathway, which, together with our functional perturbation experiments, supports increased involvement of TGF-β1 signaling in the endothelial response to flow. We have revised the Results section to make this distinction explicit (p. 9, lines 18-23).

We also visualized fluid transport using 2.0 μm fluorescent beads. The beads were carried radially away from the point of impingement and there was no visible accumulation near the stagnation region (revised Supplementary Video 1, see part A). However, it should be noted that although this provides a qualitative visualization of convective transport within the device, the beads differ substantially from soluble proteins in size, diffusivity, and surface interactions and therefore cannot be used to infer the spatial distribution of cellular secreted factors directly.

Taken together, our data indicate that TGF-β1 signaling contributes to the flow-induced increase in endothelial traction forces but does not function as the directional cue underlying reverse rheotaxis. We cannot exclude the possibility that gradients of other secreted molecules contribute to the migratory response, and we now explicitly acknowledge this limitation in the revised discussion (p. 14, line 4-9). Definitive determination of the spatial distribution of soluble signaling molecules within the impinging-flow geometry would require dedicated transport simulations or direct molecular measurements, which lie beyond the scope of the present study.

2. The current metrics supporting the notion of flow leading to TGF-β1 activation are

transcriptomic, and assembled from downstream and parallel readouts. While evidence of transcription is great, transcription does not always lead to translation as the cell has multiple mechanisms that could lead to the decoupling of transcription and translation. With that being said, direct evidence showing that flow itself activates TGF-β1 signaling at the protein level is not demonstrated here. A demonstration of changes in protein levels via immunoblots or addressing this point in some way in the manuscript is critical if the authors want to make this claim.

Please see our response above. In brief, we agree that transcriptional changes alone do not demonstrate increased TGF-β1 protein abundance or activation, and we have revised the manuscript to avoid making this inference. To further address this point, we performed immunostaining for TGF-β1 in HMEC-1 under static and flow conditions. TGF-β1 exhibited a striated, predominantly pericellular distribution, with no obvious qualitative difference between static and flow conditions (new Supplementary Fig. 5E and p. 9, lines 18-23). Because this staining does not distinguish between intracellular, extracellular, latent, and active TGF-β1, we do not interpret these data as evidence for increased TGF-β1 activation at the protein level. Importantly, our transcriptomic evidence is not based solely on increased *TGFB1* expression. Pathway enrichment analysis identified broader regulation of TGF-β1 signaling in response to flow, with multiple genes associated with this pathway being differentially expressed. We have revised the Results to make clear that these data support flow-induced regulation of the TGF-β1 signaling pathway, rather than directly demonstrating increased TGF-β1 protein abundance or activation.

3. Each of the pharmacological inhibitors used rests on a single molecule whose selectivity profile and in some cases off target effects should be acknowledged, with appropriate citations. Y-27632, beyond ROCK1/2, inhibits the PKC-related kinase PRK2/PKN (IC50 ≈ 600 nM) and, at higher concentrations, PKC and PKA (Ishizaki et al., Mol Pharmacol 2000, 57:976; independent selectivity profiling in Davies et al., Biochem J 2000, 351:95). PF-573228 is, to its credit is roughly 50–250-fold selective for FAK (IC50 ≈ 4 nM) over Pyk2 and kinases such as CDK1/7 and GSK-3β (Slack-Davis et al., J Biol Chem 2007, 282:14845) so its liability is residual Pyk2/off-kinase activity at higher doses rather than frank dual inhibition; the authors could cite this selectivity as a strength of their choice. SB-525334, though ALK5-selective, is only about 4-fold more potent against ALK5 (IC50 ≈ 14 nM) than against the closely related activin receptor ALK4 (IC50 ≈ 59 nM), while being inactive against ALK2/3/6 (Grygielko et al., J Pharmacol Exp Ther 2005, 313:943); at the 10 μM working dose used here it therefore acts as an effective ALK5/ALK4 (TGF-

β/activin) dual inhibitor. Since activin/nodal signaling also converges on SMAD2/3, the SB-525334 result does not cleanly isolate the TGF-β type I receptor, and this point should be acknowledged in the manuscript where appropriate.

We thank the Reviewer for pointing out that the selectivity profiles and potential off-target activities of the pharmacological inhibitors should be acknowledged. We have therefore revised the manuscript to include a discussion of the known selectivity and limitations of these inhibitors and the suggested references for Y-27632 (Ishizaki et al., 2000, Davies et al., 2000), PF-573228 (Slack-Davis et al., 2007), and SB-525334 (Grygielko et al., 2005) (p. 10, lines 6-7; p. 10, lines 22-24; p. 11, lines 23-27). Moreover, as also pointed to comment #4, we have performed experiments with additional pharmacological inhibitors of FAK (namely FAK-14 (Golubovskaya et al., 2008)) and TGF-β1R (namely LY-364947 (Singh et al., 2003)) as orthogonal confirmations to the claims made and included references for potential off-target activities for these as well (p. 10, lines 16-18; p. 11, lines 29-31; revised Supplementary Fig 7B-D and revised Supplementary Fig 8E-G).

4. As a follow-up to point 3, a second FAK inhibitor or FAK/PTK2 knockdown and/or where feasible, orthogonal confirmation for the TGF-β portion would substantially harden the mechanistic conclusions.

We thank the Reviewer for this suggestion. To provide orthogonal pharmacological validation of the role of FAK, we performed key experiments using a second structurally distinct FAK inhibitor, FAK-14 (Golubovskaya et al., 2008), in addition to PF-573228. Treatment of HMEC-1 with FAK-14, similar to PF-573228, did not alter singificanlty the generated traction stresses but reduced overall cell migration speed and inhibited reverse rheotaxis (revised Supplementary Fig. 7B-D), supporting the conclusion that FAK signaling is required for this response and reducing the likelihood that the observed phenotype arises from off-target effects of a single inhibitor.

Moreover, inhibition of TGF-β signaling with the structurally distinct ALK5 inhibitor LY-364947 (Singh et al., 2003), recapitulated the effects observed with SB-525334 (Supplementary Fig. 8E-G), namely no increase in traction forces was observed during exposure of HMEC-1 to flow while rheotaxis remained unaffected. This now provides orthogonal confirmation of the involvement of TGF-β1 in regulating the increase in HMEC-1 traction stresses in response to flow.

5. The active-focal-adhesion-turnover data complicate the framing that FAK specifically governs

adhesion turnover: by the authors' own description, Y-27632 (ROCK) reduces turnover most strongly, with PF-573228 having a lesser effect on the same readout. The discussion should reconcile this, since it bears on how cleanly the FAK and ROCK contributions can be separated.

We thank the Reviewer for this insightful comment. We agree that our focal adhesion turnover data indicate that ROCK inhibition has an equal or even greater effect than FAK inhibition on adhesion dynamics (revised Supplementary Figure 7A). This observation is consistent with previous studies showing that ROCK regulates not only actomyosin contractility but also focal adhesion maturation and turnover (Bhadriraju et al., 2007, Srinivasan et al., 2019). We have revised accordingly the manuscript (p. 10, lines 32-33 and p. 11, lines 7-10) to clarify that ROCK and FAK regulate overlapping aspects of focal adhesion dynamics through distinct but interconnected mechanisms. ROCK primarily controls actomyosin-generated tension, which is required for focal adhesion maturation and mechanical stabilization, whereas FAK functions as a mechanosensitive signaling hub that coordinates adhesion assembly and disassembly. Consequently, the stronger effect of Y-27632 on focal adhesion turnover likely reflects the broad suppression of cytoskeletal tension, while PF-573228 primarily disrupts FAK-dependent adhesion signaling. We have therefore modified the text to avoid implying that FAK is the sole regulator of focal adhesion turnover. Rather, our data support complementary roles for ROCK and FAK in regulating adhesion dynamics, with FAK emerging as the critical regulator of flow-directed migration.

To further place our findings in context with what is known regarding endothelial mechanotransduction and rheotaxis, we have also expanded the discussion to include the following paragraph: "S1P/S1PR1 signaling has also been shown to activate FAK and promote focal adhesion remodeling and cytoskeletal reorganization in EC, thereby enhancing endothelial barrier function (Belvitch and Dudek, 2012). Importantly, upstream migration of lymphatic EC under flow has been reported to depend on S1PR1 signaling (Surya et al., 2016), raising the possibility that S1PR1 may act upstream of FAK as part of a broader mechanotransduction pathway regulating directional endothelial migration."

6. The directional flip is only shown as representative kymographs and does not appear to be accompanied by any explicit statistical testing contrasting forward versus reverse migration for any of the directional data. Proving statistical significance would help definitively formalize the "true polarity reversal" claim made here.

Per the Reviewer's recommendation, we have added boxplots in revised Fig. 2D-E and revised Supplementary Fig. 6C to enable statistical comparison of the rheotactic behavior across the different endothelial cell types, including cells treated with pharmacological inhibitors. To generate these boxplots, the cumulative (integrated) radial displacement of endothelial cells as a function of distance from the jet center was calculated from the corresponding kymographs for each recording (as illustrated in Supplementary Fig. 2A). These values were then averaged over four consecutive time intervals following flow onset (0-5 h, 5-10 h, 10-15 h, and 15-20 h). The resulting mean radial displacements for each condition and time interval were compared statistically using either the Wilcoxon rank-sum test (WRST) or one-way ANOVA with the appropriate multiple-comparison correction, as indicated.

7. HMEC-1 and hCMEC/D3 are both immortalized lines that carry well-documented junctional and barrier abnormalities, which is relevant given that monolayer stress microscopy is framed as a readout of barrier-integrity strength. This point could be improved by verifying that these cell lines express the expected traditional barrier molecules, utilizing primary microvascular endothelial cells, or at the bare minimum acknowledging this point in the manuscript as a limitation.

We thank the Reviewer for this comment. We agree that HMEC-1 and hCMEC/D3 are immortalized endothelial cell lines and therefore do not fully recapitulate the junctional and barrier properties of primary endothelial cells. We have now explicitly acknowledged this limitation in the revised discussion (p. 16, lines 3-7). Nevertheless, both cell lines have been extensively used and validated as endothelial models that retain key aspects of endothelial morphology, organization, and barrier function (Weksler et al., 2013, Ades et al., 1992). Indeed, when we examined canonical endothelial junctional markers looking at our RNA-sequencing dataset, we found that both HMEC-1 and hCMEC/D3 express CDH5, PECAM1, TJP1, ESAM, OCLN, and CLDN5 like do primary HUVEC (Fig. R2). Nevertheless, while TJP1 and OCLN expression was comparable across HMEC-1, hCMEC/D3, and HUVECs, CDH5, PECAM1, ESAM, and CLDN5 were more highly expressed in HUVEs, highlighting quantitative differences between these endothelial models (or between these two microvascular endothelial cell lines and primary HUVEC) (Fig. R2). Moreover, per the reviewer's recommendation we revised the results section refering to what monolayer stress microscopy measure and now state: "We also performed monolayer stress microscopy (MSM) to calculate EC monolayer stresses (i.e., inter- and intra-cellular stresses), a measure of endothelial barrier mechanics."

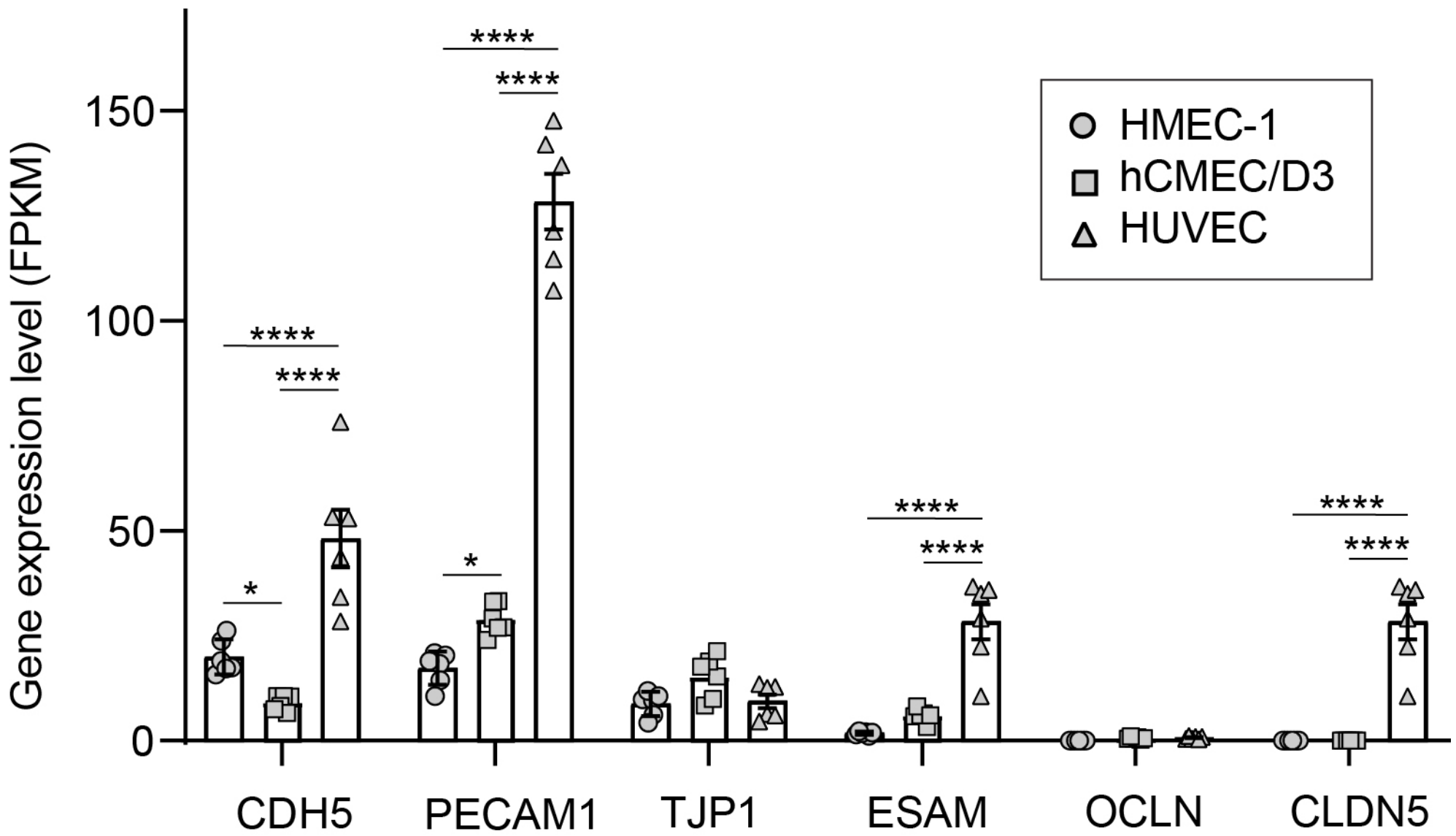

Figure R2: Boxplots of gene expression level (FPKM, fragments per kilobase of transcript per million mapped reads) for HMEC-1, hCMEC/d3, and HUVEC in monolayer under static conditions. RNA sequencing was performed in sextuplicates and individual values of samples are superimposed. Boxplots show mean and standard error of the mean (SEM). Genes shown are CDH5 (encoding VE-cadherin), PECAM1 (encoding PECAM-1), TJP1 (encoding ZO-1), ESAM (encoding ESAM), OCLN (encoding occludin), and CLDN5 (encoding claudin-5). ANOVA with Šídák's multiple comparisons was perofrmed: *: $p<0.05$; ****: $p<0.0001$.

8. A major issue exist regarding monolayer stress microscopy calculations. As acknowledged by the authors, MSM sets displacements to zero at the image boundary, but only a small portion of the monolayer boundary can be seen and the rest of the monolayer most likely extends well beyond the field of view shown here. Therefore, a zero-displacement boundary condition at regions where the monolayer boundary extends beyond the field of view is incorrect as this is physically impossible. Furthermore, such assumptions will bias inferred intercellular stresses, and this effect most likely will be amplified in a radially graded field. This is an inherent limitation of MSM when done with monolayers that expand beyond the field of view, which is enhanced here. This point needs to be addressed explicitly in the manuscript as this is a significant limitation and source of error for the msm calculations.

We thank the Reviewer for this important comment. MSM does require assumptions regarding boundary conditions and analyzing only a finite region of a larger monolayer represents an inherent limitation of the method. As previously demonstrated, the boundary assumptions used in classical MSM predominantly introduce artifacts near the optical edges, whereas stresses within the interior of the analyzed region are substantially less affected (Tambe et al., 2013). Nevertheless, because our experiments involve a radially graded flow field and the endothelial

monolayer extends beyond the field of view, we acknowledge that these assumptions may introduce additional uncertainty in the reconstructed absolute stress values. Because all experimental conditions were acquired using identical imaging geometry and analyzed with the same computational pipeline and boundary assumptions, any systematic bias is expected to affect all conditions similarly, such that the relative differences underlying our conclusions continue to hold (e.g., that monolayer stresses weaken with addition of Y-27632, or that flow leads to an increase in magnitude of monolayer stresses). We have now explicitly acknowledged this limitation in the revised discussion (p. 16, lines 15-21) as follows: "A further methodological limitation is that MSM was performed on image regions that did not encompass the entire monolayer. Consequently, the boundary conditions required for stress reconstruction represent an approximation and may influence the reconstructed absolute stress values, particularly near the image boundaries (Tambe et al., 2013). Nevertheless, because identical imaging geometries and analysis parameters were applied across conditions, relative comparisons between conditions remain informative."

9. The authors should consider adding qualifiers or reframing their claim of "mechanical memory" as this claim appears to be somewhat partially weakened within the paper. For example, Supp. Fig. 3 notes only "minor recovery" for hCMEC/D3, and Supplementary Fig. 4B shows hCMEC/D3 strain energy post-flow returning to a level not significantly different from static controls, whereas HMEC-1 traction remains elevated. If this truly is a conserved behavior this reviewer would expect comparable recovery levels regardless of cell type, but this is not the case. "Mechanical memory" appears to potentially be cell-type-specific, and not a general behavior. This is in addition to the point mentioned above that both cell lines are immortalized as well. This point needs to be addressed in the manuscript.

We thank the Reviewer for this thoughtful comment and agree that our original wording overstated the generality of the proposed "mechanical memory" response. While both hCMEC/D3 and HMEC-1 cells continue to exhibit rheotaxis after flow cessation (i.e., similar kinematical response), their dynamics differ: HMEC-1 cells maintain elevated traction forces for at least 10 h after flow removal, whereas hCMEC/D3 cells show a substantial recovery toward static levels immediately following flow cessation. This finding suggests that the persistence of traction forces is cell type-dependent rather than a universal endothelial response, and we now discuss this distinction explicitly in the revised results and discussion sections (p.8, lines 3-4; p. 13, lines 5-10; p. 15, lines 11-17). As also pointed out in response to comment #7, in the revised discussion section we

now acknowledge the limitation regarding the use of cell lines rather than primary cells (p. 16, lines 3-7).

Minor comments

10. Supplementary Fig. 4 is titled and structured for hCMEC/D3, yet panel 4C presents HMEC-1 monolayer-stress data (explicitly referencing main Fig. 4A). Either the label is wrong or the panel is misplaced; in either case the HMEC-1 monolayer-stress quantification that supports main Fig. 4 should not be buried in the hCMEC/D3 figure without a clear cross-reference.

We thank the Reviewer for identifying this labeling error. We have corrected the title and labeling of the revised Supplementary Fig. 4 to accurately reflect the content of the figure and clearly indicate that panel C presents the HMEC-1 monolayer stress analysis corresponding to the main Fig. 4.

11. Throughout the main manuscript and supplementary document the FAK inhibitor is referred to as "PF-573228," "PF537228," and "PF573228". Please use one consistent name to refer to this inhibitor.

We have corrected the name of the inhibitor to be consistent.

12. Multiple typos exits and extensive proofreading is needed. For example,"Supplementarymentary," – line 27, line 132, "progression.ostrowski" – line 64, RNA "integrality", "mapp.i.ng,", and "Signs-Aldrich,". However, there are too many to put them all and more exist.

We thank the Reviewer for bringing these errors to our attention. The manuscript has been carefully proofread, and all identified typographical, spelling, and formatting errors have been corrected throughout the text.

13. Post-flow observation is 10 h in Figs. 3–4, but 5 h in Figs. 6–7; this matters for cross-figure comparison of the persistence claim and should be reconciled or explained.

We thank the Reviewer for this observation. The different post-flow observation periods reflect the distinct aims of these experiments. In Figs 3 and 4, we specifically investigated the persistence

of the flow-induced biomechanical phenotype following flow cessation and therefore monitored the cells for up to 10 h after flow was stopped. In contrast, Figs 6 and 7 were designed to investigate the biomechanical mechanisms underlying rheotaxis and increases in EC traction during flow and pharmacological treatment. While these experiments could have been terminated immediately after flow cessation, we intentionally continued imaging for an additional 5 h to determine whether the observed rheotactic behavior and increase in EC traction persisted after removal of the flow stimulus (in the cases where it was not blocked), thereby providing an internal control for the reversibility of the response. We have clarified this rationale in the revised Methods section (p. 25, lines 18-27).

14. Although line, plots of shear stress and SSG are provided, figures of the spatial shear stress gradient (or color plots) are not provided. This is important as it would provide a visual of where the SSG are in relation to the cells shown in the phase images and in relation to migration, traction data, etc.

The spatial relationship between the shear stresses and gradient and the analyzed cells was represented in Fig. 1 and throughout the subsequent figures of the first version of the manuscript. We acknowledge, however, that this might not have been clearly explained. Fig. 1A shows a schematic of the impinging-flow device, including the position of the endothelial monolayer and the fluid-velocity field. Fig. 1B shows the magnitude of the wall shear stress as a function of radial distance from the center of the jet, while Fig. 1C shows the corresponding shear stress gradient as a function of the same radial distance. The plotted range extends to approximately 1 mm from the jet center, matching the dimensions of the microscopy field of view; at larger radial distances, the wall shear stress is negligible (~0.05 Pa). In Fig. 1B-C, the vertical dashed lines indicate equally spaced radial positions from the jet center. These positions correspond directly to the white concentric rings overlaid on all subsequent microscopy images. For example, in Fig. 1D, only the lower-right quadrant of the field of view is shown under flow conditions (and this is the case in most videomicroscopy data shown in this study, for clarity reasons). The jet center therefore lies at the upper-left corner of the image, and each white ring corresponds to one of the radial positions indicated by the dashed lines in Fig. 1B-C. The same spatial correspondence is used in all subsequent analyses, including the migration field, traction stresses and monolayer stresses. We have revised the methods section (p. 21, lines 17-23) and the legend of Fig. 1 to better explain this correspondence.

15. As a follow up to point 14, It also is not clear where the cells, ECM displacement data, traction data, etc. are physically located in relation to the radial distance. Looking at the plots provided the shear stress seems to approach zero at a radial distance of < 1mm (although the chamber itself has a max radial distance of 2 mm). With that being said, some kind of schematic or illustration showing where the cells are located would be helpful. In other words, something showing what location in the chamber the cells are located and where this data subsequently corresponds to would be helpful.

We explained this in the point above (14).

References

ADES, E. W., CANDAL, F. J., SWERLICK, R. A., GEORGE, V. G., SUMMERS, S., BOSSE, D. C. & LAWLEY, T. J. 1992. HMEC-1: Establishment of an Immortalized Human Microvascular Endothelial Cell Line. *Journal of Investigative Dermatology,* 99**,** 683-690.

BELVITCH, P. & DUDEK, S. M. 2012. Role of FAK in S1P-regulated endothelial permeability. *Microvascular Research,* 83**,** 22-30.

BHADRIRAJU, K., YANG, M., ALOM RUIZ, S., PIRONE, D., TAN, J. & CHEN, C. S. 2007. Activation of ROCK by RhoA is regulated by cell adhesion, shape, and cytoskeletal tension. *Exp Cell Res,* 313**,** 3616-23.

DAVIES, S. P., REDDY, H., CAIVANO, M. & COHEN, P. 2000. Specificity and mechanism of action of some commonly used protein kinase inhibitors. *Biochem J,* 351**,** 95-105.

DOLAN, J. M., KOLEGA, J. & MENG, H. 2013. High wall shear stress and spatial gradients in vascular pathology: a review. *Ann Biomed Eng,* 41**,** 1411-27.

EGOROVA, A. D., VAN DER HEIDEN, K., VAN DE PAS, S., VENNEMANN, P., POELMA, C., DERUITER, M. C., GOUMANS, M. J., GITTENBERGER-DE GROOT, A. C., TEN DIJKE, P., POELMANN, R. E. & HIERCK, B. P. 2011. Tgfβ/Alk5 signaling is required for shear stress induced klf2 expression in embryonic endothelial cells. *Dev Dyn,* 240**,** 1670-80.

GOLUBOVSKAYA, V. M., NYBERG, C., ZHENG, M., KWEH, F., MAGIS, A., OSTROV, D. & CANCE, W. G. 2008. A small molecule inhibitor, 1,2,4,5-benzenetetraamine tetrahydrochloride, targeting the y397 site of focal adhesion kinase decreases tumor growth. *J Med Chem,* 51**,** 7405-16.

GRYGIELKO, E. T., MARTIN, W. M., TWEED, C., THORNTON, P., HARLING, J., BROOKS, D. P. & LAPING, N. J. 2005. Inhibition of gene markers of fibrosis with a novel inhibitor of transforming growth factor-beta type I receptor kinase in puromycin-induced nephritis. *J Pharmacol Exp Ther,* 313**,** 943-51.

HINZ, B. 2015. The extracellular matrix and transforming growth factor-β1: Tale of a strained relationship. *Matrix Biol,* 47**,** 54-65.

ISHIZAKI, T., UEHATA, M., TAMECHIKA, I., KEEL, J., NONOMURA, K., MAEKAWA, M. & NARUMIYA, S. 2000. Pharmacological properties of Y-27632, a specific inhibitor of rho-associated kinases. *Mol Pharmacol,* 57**,** 976-83.

OHNO, M., COOKE, J. P., DZAU, V. J. & GIBBONS, G. H. 1995. Fluid shear stress induces endothelial transforming growth factor beta-1 transcription and production. Modulation by potassium channel blockade. *J Clin Invest,* 95**,** 1363-9.

OSTROWSKI, MAGGIE A., HUANG, NGAN F., WALKER, TRAVIS W., VERWIJLEN, T., POPLAWSKI, C., KHOO, AMANDA S., COOKE, JOHN P., FULLER, GERALD G. & DUNN, ALEXANDER R. 2014. Microvascular Endothelial Cells Migrate Upstream and Align Against the Shear Stress Field Created by Impinging Flow. *Biophys. J.,* 106**,** 366-374.

REMUZZI, A., DEWEY, C. F., JR., DAVIES, P. F. & GIMBRONE, M. A., JR. 1984. Orientation of endothelial cells in shear fields in vitro. *Biorheology,* 21**,** 617-30.

SINGH, J., CHUAQUI, C. E., BORIACK-SJODIN, P. A., LEE, W.-C., PONTZ, T., CORBLEY, M. J., CHEUNG, H. K., ARDUINI, R. M., MEAD, J. N., NEWMAN, M. N., PAPADATOS, J. L., BOWES, S., JOSIAH, S. & LING, L. E. 2003. Successful shape-Based virtual screening: The discovery of a potent inhibitor of the type I TGFβ receptor kinase (TβRI). *Bioorganic & Medicinal Chemistry Letters,* 13**,** 4355-4359.

SLACK-DAVIS, J. K., MARTIN, K. H., TILGHMAN, R. W., IWANICKI, M., UNG, E. J., AUTRY, C., LUZZIO, M. J., COOPER, B., KATH, J. C., ROBERTS, W. G. & PARSONS, J. T. 2007. Cellular characterization of a novel focal adhesion kinase inhibitor. *J Biol Chem,* 282**,** 14845-52.

SRINIVASAN, S., DAS, S., SURVE, V., SRIVASTAVA, A., KUMAR, S., JAIN, N., SAWANT, A., NAYAK, C. & PURWAR, R. 2019. Blockade of ROCK inhibits migration of human primary keratinocytes and malignant epithelial skin cells by regulating actomyosin contractility. *Scientific Reports,* 9**,** 19930.

SURYA, V. N., MICHALAKI, E., HUANG, E. Y., FULLER, G. G. & DUNN, A. R. 2016. Sphingosine 1-phosphate receptor 1 regulates the directional migration of lymphatic endothelial cells in response to fluid shear stress. *J R Soc Interface,* 13.

TAMBE, D. T., CROUTELLE, U., TREPAT, X., PARK, C. Y., KIM, J. H., MILLET, E., BUTLER, J. P. & FREDBERG, J. J. 2013. Monolayer Stress Microscopy: Limitations, Artifacts, and Accuracy of Recovered Intercellular Stresses. *PLOS ONE,* 8**,** e55172.

WEKSLER, B., ROMERO, I. A. & COURAUD, P. O. 2013. The hCMEC/D3 cell line as a model of the human blood brain barrier. *Fluids Barriers CNS,* 10**,** 16.
