# Supplementary material for: FAK- and TGF-β1-dependent biomechanical alterations in microvascular endothelial cells exposed to fluid shear stress gradients
Source: Commun Biol. 2026 Nov 3;9:1451. doi: 10.1038/s42003-026-11283-9 (PMC13731646; doi:10.1038/s42003-026-11283-9)
Supplement: Supplementary file 2 [file 42003_2026_11283_MOESM2_ESM.pdf]

SUPPLEMENTARY INFORMATION

SUPPLEMENTARY FIGURES AND FIGURE LEGENDS

SUPPLEMENTARY FIGURE 1

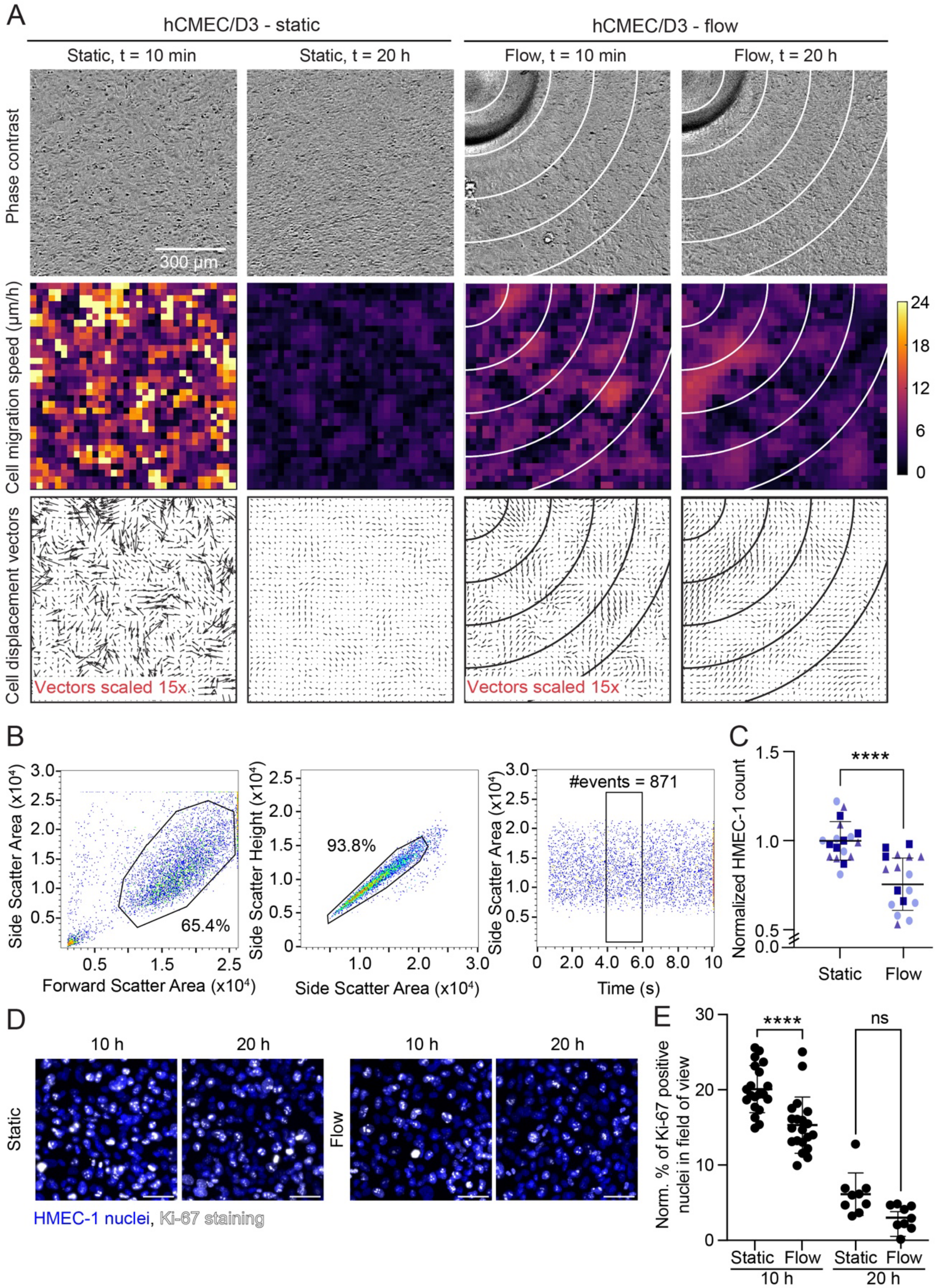

Supplementary Fig. 1: SSG slows hCMEC/D3 migration speed while enhancing motion coordination.

A Representative images of hCMEC/D3 in monolayer at 10 min and 20 h under static conditions (columns 1, 2) or exposed to flow (columns 3, 4) using the impinging flow device at 3 mL/min flow rate. Rows show phase contrast images (1st), cell migration speed (µm/h, 2nd) and cell displacement vectors (3rd). Flow was initiated at t=0 min. Jet center was positioned at the center of the field of view imaged, but only a quarter of the field of view is shown. White or black contours mark concentric regions spaced 185 µm apart from the jet orifice. Cell displacements in the 3rd row are scaled 15x for all conditions. **B** Flow cytometry scatter plots illustrating gating hierarchy used to quantify HMEC-1 cell concentration per well per well following 20 h of exposure to flow or static conditions (controls). (Left) Cell population is identified and gated based on the forward scatter area (FSC-A) versus side scatter area (SSC-A) plot, with HMEC-1 cells enclosed within the black "scatter" gate. (Middle) Singlet events are plotted against acquisition time (SSC-A versus time). Events acquired during a stable 2 s interval (black box; n = 871 events) are converted to events per min and normalized to the sample flow rate to calculate the final cell concentration (cells/mL). **C** Normalized cell counts per well for HMEC-1 under static conditions or exposed to flow for 20 h, as indicated above. Numbers of cells per well were measured via flow cytometry. For each experiment, values have been normalized to the mean cell count for cells under static conditions. Mean±SD. n=18 samples analyzed originating from N=3 independent experiments denoted by different shapes. WRST, p<0.0001: ****. **D** Representative immunofluorescence images of fixed HMEC-1 in monolayer previously under static conditions or exposed to flow for 10 h (left) or 20 h (right). Images show cell nuclei (blue) superimposed with Ki-67 fluorescence (white, proliferation marker) for cells under static conditions (top) and exposed to 3 mL/min flow (bottom). Scale bar: 50 µm. **E** Normalized percentage of Ki-67 positive nuclei in immunofluorescence images of fixed HMEC-1 cells in monolayer under static conditions or exposed to flow for 10 h or 20 h. For each time point, values were normalized to the mean of the corresponding static control. Mean±SD. An average of n=15 images were analyzed from N=4 independent wells. WRST, p<0.01: **; p<0.0001: ****.

SUPPLEMENTARY FIGURE 2

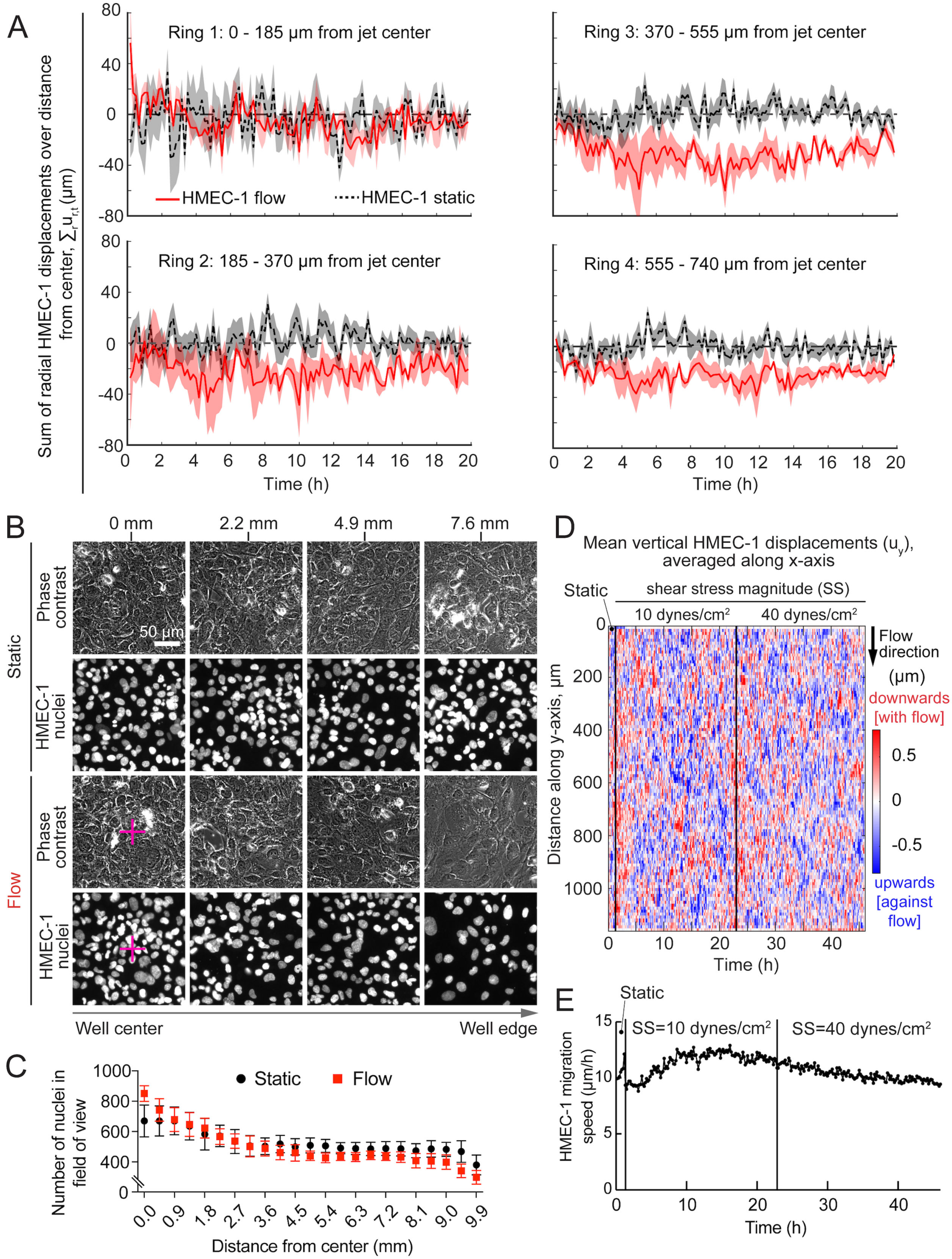

Supplementary Fig. 2: SSG triggers collective μEC migration against the flow direction.

A Integral of radial HMEC-1 displacement, u_r (y-axis, μm) over radial distance plotted as a function of time (x-axis, h), for HMEC-1 in monolayer under static conditions (controls, black) or exposed to flow (red) using the impinging flow device at 3 mL/min flow rate. Displacements' integrals are binned by distance from the jet center in 185 μm intervals, each corresponding to a distinct region of the SS profile: 0–185 μm (top-left), positive SSG; 185–370 μm (bottom-left), region of maximum SS and negative SSG; 370–555 μm (top-right), minimum SSG; and 555–740 μm (bottom-right), negative SSG. Mean±SEM of n=10±4 recordings from at least N=3 independent experiments. Negative (positive) values indicate cell migration toward (away from) the jet center. Note that most negative values (indicating most reverse rheotaxis) are seen at Ring 3. **B** Representative images of HMEC-1 monolayers acquired at increasing radial distances from the center of the well (x-axis, mm), which coincides with the center of the impinging jet for flow-exposed cells. Images were acquired over an extended radial range (up to 7.6 mm) to assess whether flow-induced changes in cell density were restricted to the region directly influenced by the impinging jet or extended across the cell-covered area of the well. Rows show phase-contrast images (1, 3) and corresponding images of cell nuclei (2, 4). Under static conditions, cell/nuclei density remains similar across radial positions. In contrast, following flow exposure, cell/nuclei density is higher near the center of the well, consistent with cells undergoing reverse rheotaxis toward the jet center, and lower at larger radial distances. The jet center (r = 0) is indicated by a magenta cross. **C** Number of HMEC-1 nuclei across the field of view, plotted as a function of distance from the well center to the well edge, for cells under static conditions (black) and after 20 h of flow exposure at 3 mL/min (red). Data represent the mean±95% confidence interval from N=3 independent experiments. **D** Kymograph of mean vertical displacement u_y of HMEC-1 cells, averaged along the x-axis, plotted as a function of time (x-axis, h) and vertical position (y-axis, μm). Cells were under static conditions from 0–1 h, exposed to constant shear stress of 10 dynes/cm² from 1 to 23 h, and then to 40 dynes/cm² from 23–46 h. Shear flow was applied vertically, from top to bottom (along the y-axis of the field of view). HMEC-1 did not exhibit any consistent directional migration under either magnitude of constant shear stress. **E** HMEC-1 migration speed (y-axis, μm/h) over time (x-axis, h) for the same recording as in panel D.

SUPPLEMENTARY FIGURE 3

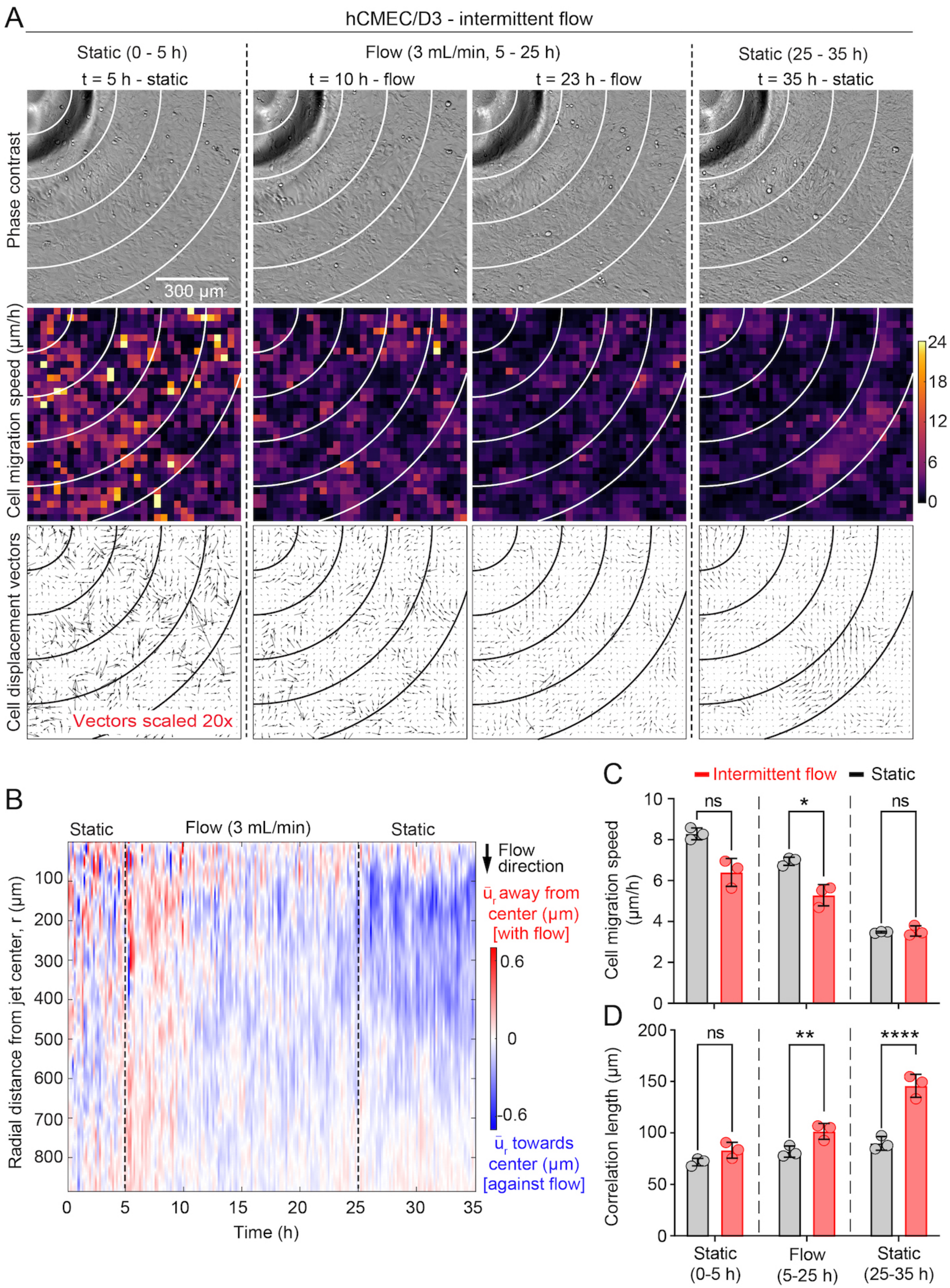

Supplementary Fig. 3: Flow exposure reduces hCMEC/D3 migration speed with minor recovery occurring after flow cessation.

A Representative phase contrast images, cell migration speed (μm/h), and cell displacement vectors (scaled 20x) for hCMEC/D3 in monolayer at different time points. Cells were imaged without flow for ~5 h, followed by 20 h during flow (between dashed black lines) and an additional 10 h under static conditions after flow cessation. Jet center was positioned at the center of the field of view imaged, but only a quarter of the field of view is shown. White or black contours mark concentric regions spaced 185 μm apart from the jet orifice. **B** Kymograph of mean radial cell displacements u_r (μm) as a function of radial distance r (μm) and time (h) for the same recording as in panel A. **C** Bar plot showing the mean cell migration speed (y-axis, μm/h) of hCMEC/D3 maintained under static conditions throughout the experiment (gray) or exposed intermittently to flow (red). Averaging was performed over three time windows: 0–5 h after the start of recording, during which both groups were under static conditions; 5–25 h, during which the intermittent-flow group was exposed to flow; and 25–35 h, during which both groups were again under static conditions. Mean±SD, N=3 independent recordings. Superimposed circles show average values for independent recordings. WRST, $p>0.05$: non-significant, ns; $p<0.05$: *; $p<0.01$: **; $p<0.0001$: ****. **D** Same as panel C, but showing mean correlation length (y-axis, μm) of cell motion.

SUPPLEMENTARY FIGURE 4

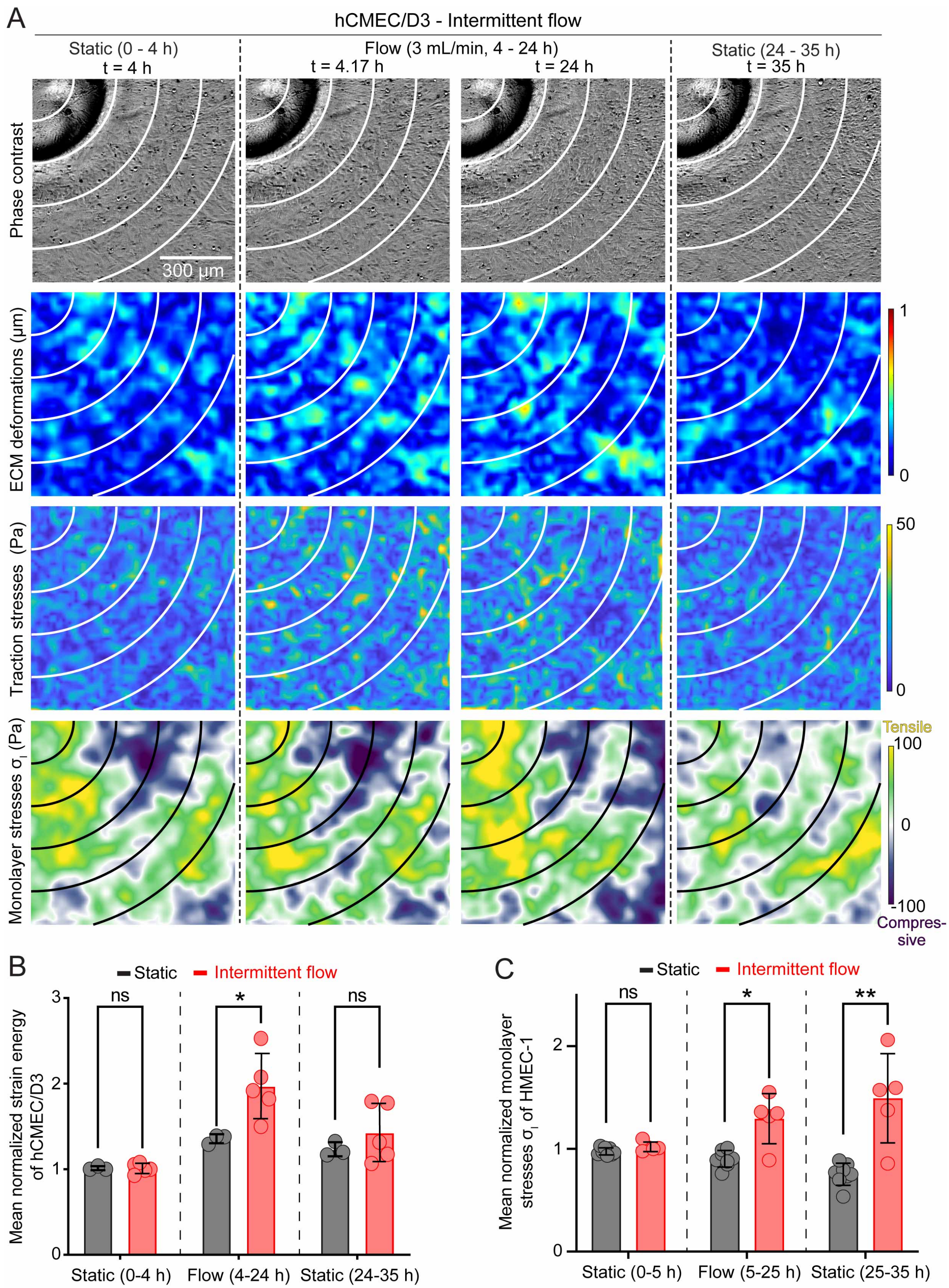

Supplementary Fig. 4: In contrast to HMEC-1, flow in hCMEC/D3 induces a sustained increase in traction and monolayer stresses, which return toward static levels after flow cessation.

A Representative images of hCMEC/D3 monolayer at various stages: before flow (1st column), immediately after flow initiation (2nd), 20 h into flow (3rd), and 10 h post-flow cessation (4th). Rows show phase contrast (1st), ECM deformations (2nd, μm), cellular traction stresses (3rd, Pa), and monolayer tensile stresses, σ_l (4th, Pa). Cells on a 3 kPa matrix were exposed to flow using the impinging flow device. Jet center was positioned at the center of the field of view imaged, but only a quarter of the field of view is shown. White or black contours mark concentric regions spaced 185 μm apart from the jet orifice. **B** Bar plots of time-averaged normalized strain energy of hCMEC/D3 residing on 3 kPa hydrogel and exposed to intermittent flow or not (as shown in panel A), binned by experimental recording phase: pre-flow (0–4 h), flow (4–24 h), and post-flow (24–35 h). Red bars: intermittent flow-exposed cells; black bars: static controls. Strain energy was normalized to its value at 2 h after the start of the recording, corresponding to the midpoint of the 4-h static phase preceding flow exposure. Superimposed circles refer to average values of independent experiments. Mean±SD, N=5 independent recordings. WRST, $p>0.05$: non-significant, ns; $p<0.05$: *. **C** Bar plots of time-averaged normalized monolayer stresses of HMEC-1 residing on 3 kPa hydrogel and exposed to intermittent flow or not (as shown in Fig. 4A). Binning was performed with respect to experimental recording phase: pre-flow (0–5 h), flow (5–25 h), and post-flow (25–35 h). Red bars: intermittent flow-exposed cells; black bars: static controls. Superimposed circles refer to average values of independent experiments. Strain energy was normalized to its value at 2.5 h after the start of the recording, corresponding to the midpoint of the 5-h static phase preceding flow exposure. N=6 independent recordings were analyzed on average. Mean±SD. WRST, $p>0.05$: non-significant, ns; $p<0.05$: *; $p<0.01$: **.

SUPPLEMENTARY FIGURE 5

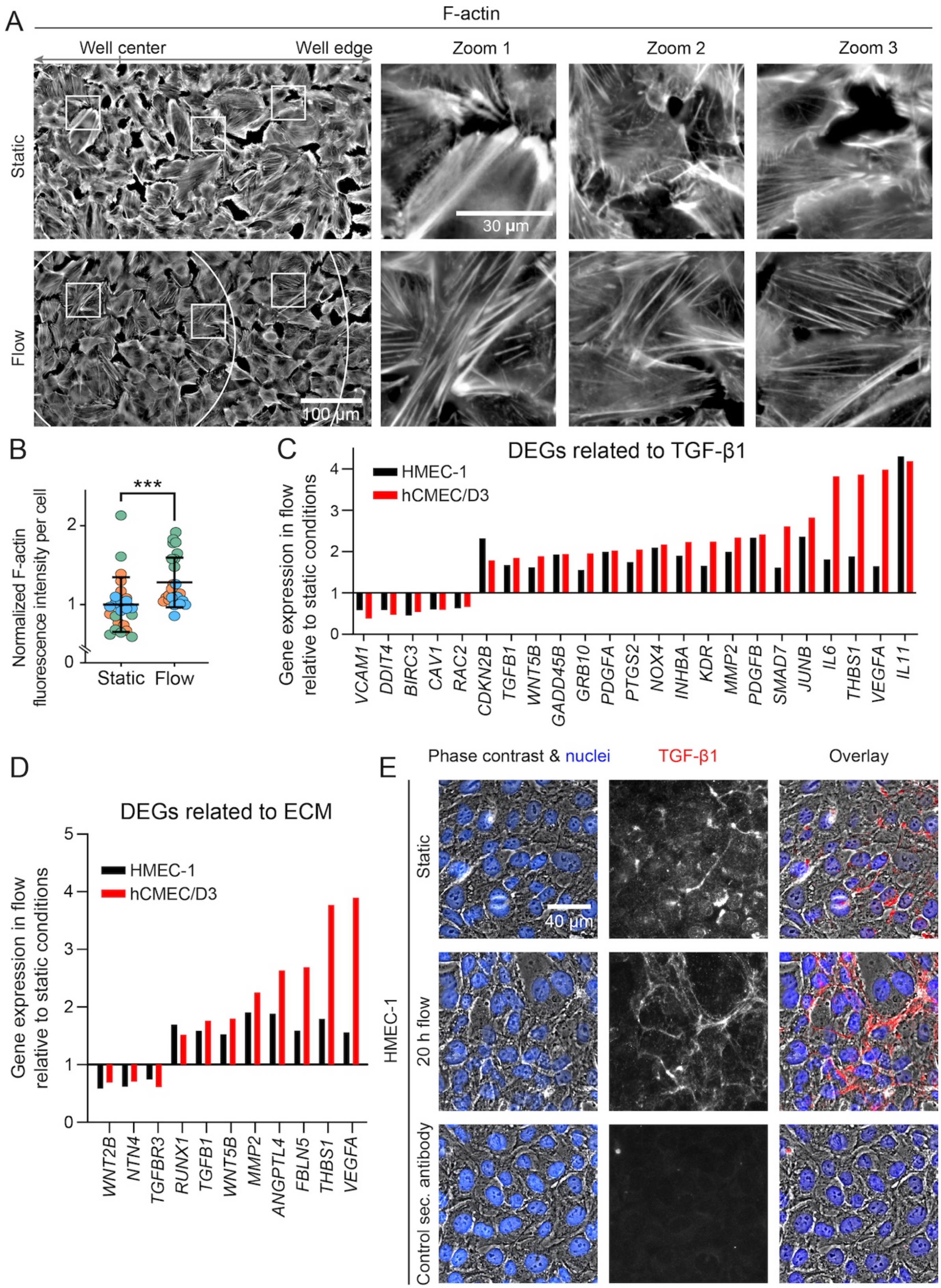

Supplementary Fig. 5: Alterations in the cytoskeleton and transcriptome of µEC under flow versus static conditions.

A F-actin immunofluorescence for HMEC-1 in monolayer under static conditions (top row) or after 20 h of flow exposure (bottom row). Maximum intensity projection images are shown. The left panels show fields of view covering three regions of the shear stress (SS) profile (flow-exposed regions marked by white rings in the bottom row). White concentric rings are spaced 185 µm apart, with the jet center corresponding to the well center indicated. Right panels show 10,000-µm² zoomed-in sections from the indicated SS profile regions (columns 2-4). **B** Plot showing the normalized mean fluorescence intensity per cell for F-actin (phalloidin staining). Immunostained samples of HMEC-1 cells under static conditions or exposed to flow for 20 h were imaged using epifluorescence microscopy as shown in Fig. 5A and quantitation was performed using basic image processing on images acquired. Normalization done with respect to the mean fluorescence intensity value of static cell samples. Each circle shows the mean fluorescence intensity per cell from a whole field of view imaged. N=900-1200 cells were analyzed for each condition at each independent experiment (three independent experiments shown in different color). WRST, $p<0.001$: ***. **C** Bar plots showing gene expression in HMEC-1 (black) and hCMEC/D3 (red) after 20 h of flow relative to static controls. Depicted are DEGs commonly up- or down-regulated in both cell lines in flow-exposed samples versus static controls and associated with the TGF-β1 signaling pathway. N=6 biological replicates per condition. **D** Same as in panel C but showing DEGs commonly up- or down-regulated in both cell lines in flow-exposed samples versus static controls and associated with ECM proteins. **E** Representative immunofluorescence images of HMEC-1 cells cultured under static conditions (top row) or exposed to flow for 20 h (middle row). Columns display phase contrast images (grayscale) overlaid with nuclei (blue), TGF-β1 immunofluorescence (grayscale), and overlay of all three signals. The bottom row shows static HMEC-1 cells stained with the secondary antibody only, serving as a negative control. Scale bar: 40 µm.

SUPPLEMENTARY FIGURE 6

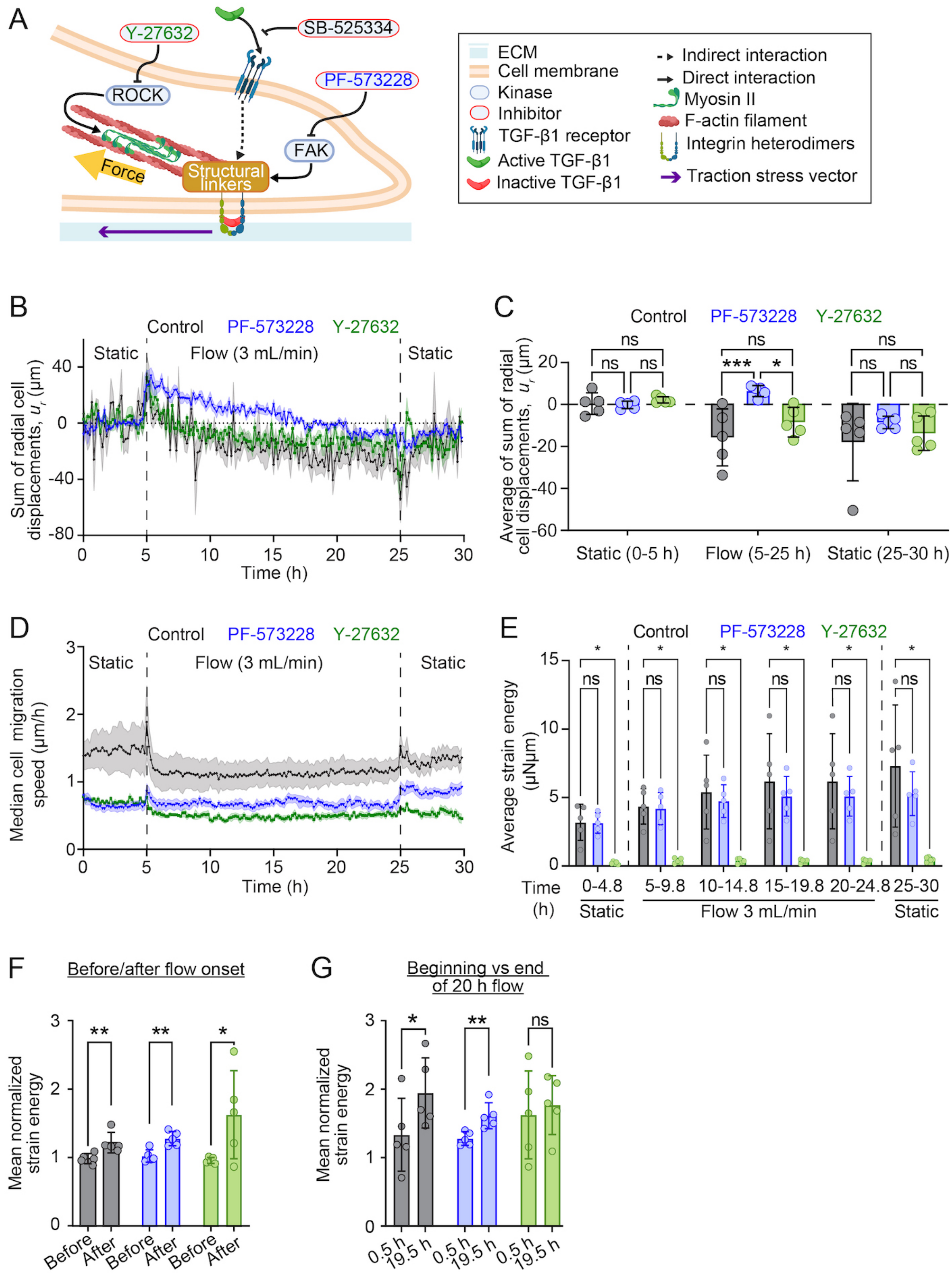

Supplementary Fig. 6: Actomyosin contractility and focal adhesion dynamics regulate µEC rheotaxis.

A Schematic illustrating the molecular targets of pharmacological inhibitors used in this study: SB-525334 (TGF-β1 receptor inhibitor) prevents active TGF-β1 binding to its receptor, which can indirectly influence focal adhesion dynamics; Y-27632 (ROCK inhibitor) inhibits Rho-associated kinase (ROCK), reducing actomyosin contractility; PF-573228 (FAK inhibitor) blocks FAK autophosphorylation, which disrupts focal adhesion maturation. **B** Integral of radial cell displacements u_r (y-axis, µm) over time (x-axis, h) for HMEC-1 cells treated with 5 µM PF-573228 (blue), 10 µM Y-27632 (green) or vehicle control (black) during a 30 h TFM recording. Cells were under static conditions from 0-5 h, exposed to flow from 5-25 h, and flow was ceased from 25-30 h. Positive (negative) values indicate movement away from (towards) the center of the jet. Time of flow exposure is shown between vertical dashed black lines. Mean±SEM, N=5 independent recordings. **C** Bar plot showing the mean integral (i.e., sum) of radial cell displacements (u_r) over the indicated time intervals for HMEC-1 monolayers treated with vehicle control (black), 5 µM PF-573228 (blue), or 10 µM Y-27632 (green). Individual recording means are overlaid as circles. Mean±SD, N=5 independent recordings. KWTD, $p>0.05$: non-significant, ns; $p<0.05$: *; $p<0.001$: ***. **D** Same as panel B but showing cell migration speed (y-axis, µm/h). **E** Bar plot of average strain energy (y-axis, µNµm) imparted by HMEC-1 in monolayer treated with vehicle control (black), or 5 µM PF-573228 (blue), 10 µM Y-27632 (green). Strain energy values are binned by time periods corresponding to microscopy recording conditions: 0–4.8 h (static), 5–9.8 h (flow), 10–14.8 h (flow), 15–19.8 h (flow), 20–24.8 h (flow), and 25–30 h (static). Note that flow was applied between 5 to 25 h post-initiation of the recording. Superimposed circles refer to average value of independent experiments. Mean±SD, N=5 independent recordings. KWTD, $p>0.05$: non-significant, ns; $p<0.05$: *. **F** Bar plot of average normalized strain energy (y-axis, µNµm) imparted by HMEC-1 in monolayer treated with vehicle control (black), or 5 µM PF-573228 (blue), 10 µM Y-27632 (green). For each condition, left bar plot refers to normalized strain energy values averaged over 1 h prior to flow exposure, while right bar plot refers to values averaged over 1 h immediately after flow exposure. Superimposed circles refer to average value of independent experiments. Mean±SD, N=5 independent recordings. WRST, $p>0.05$: non-significant, ns; $p<0.05$: *; $p<0.01$: **. **G** Same as panel F but showing normalized strain energy values averaged over 1 h immediately after flow exposure and 1 h before flow cessation (i.e., from 19 to 20 h after flow initiation).

SUPPLEMENTARY FIGURE 7

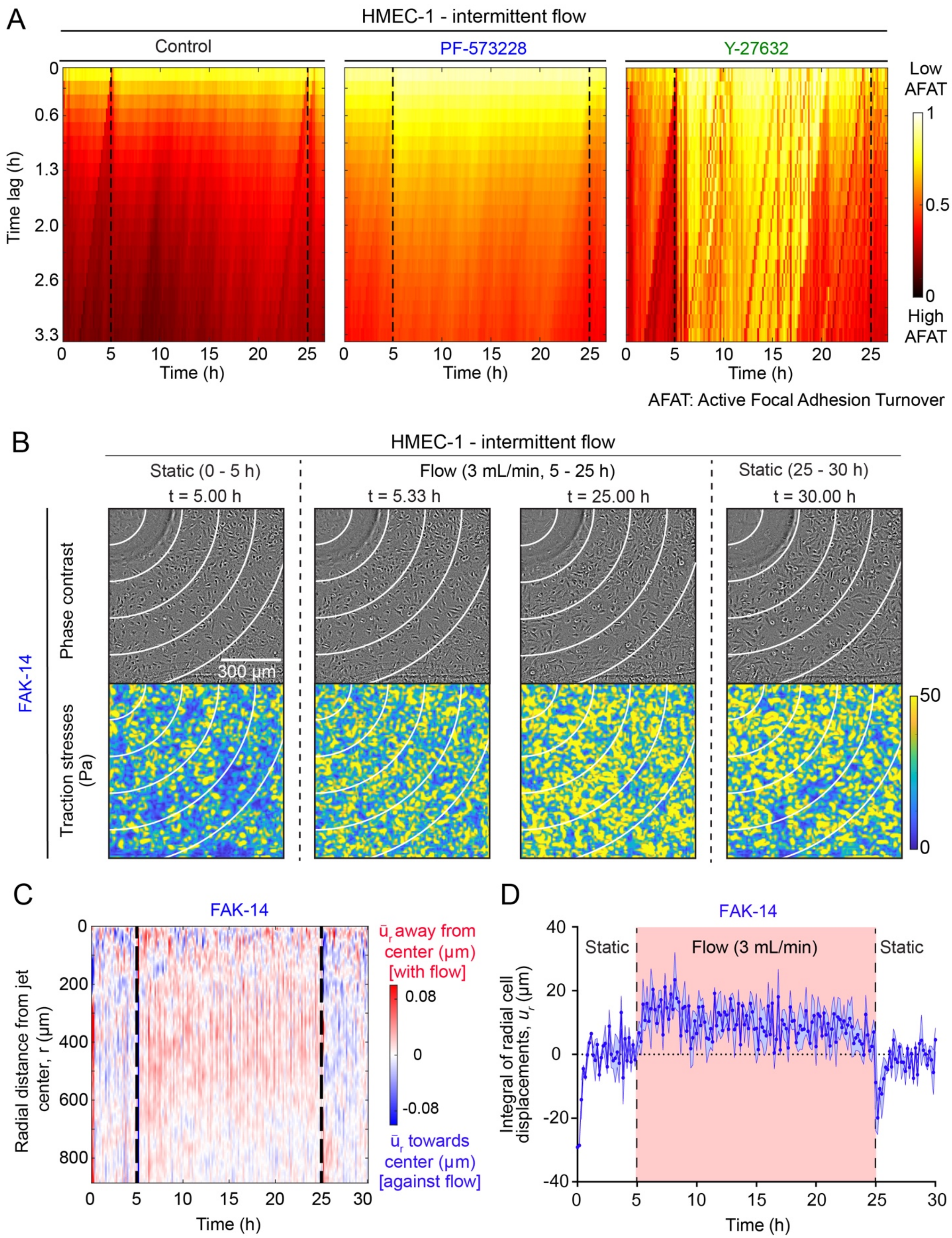

Supplementary Fig. 7: Focal adhesion dynamics and signaling regulate µEC rheotaxis.

A Visualization of traction adhesion dynamics using kymographs of the cross-correlation coefficient (colorbar) of sequential matrix deformation maps. The y-axis represents time lag (in h), showing correlations over subsequent intervals, and the x-axis represents time (in h). The more yellow values indicate decreased traction adhesion turnover. HMEC-1 in monolayer were exposed to intermittent flow and either treated with vehicle control (left), 10 µM Y-27632 (middle), or 5 µM PF-573228 (right). Cells were under static conditions from 0-5 h, exposed to flow from 5-25 h, and returned to static conditions from 25-30 h. Black lines indicate initiation and cessation of flow, respectively. Note that treatment with Y-27632 in particular, and to a lesser degree with PF-573228, significantly decreases the turnover of traction adhesions (i.e., sequential matrix deformation maps are very similar to each other). Flow exposure also appears to decrease the turnover of traction adhesions as compared to static conditions, for all three cases examined. **B** Representative images of HMEC-1 in monolayer treated with 5 µM FAK-14 at various stages: before flow exposure (1st column), immediately after flow initiation (2nd), 20 h into flow (3rd), and 5 h post-flow cessation (4th). Rows show phase contrast (1st) and cellular traction stresses (2nd, Pa). Cells on a 3 kPa matrix were exposed to flow using the impinging flow device. Jet center was positioned at the center of the field of view imaged, but only a quarter of the field of view is shown. White or black contours mark concentric regions spaced 185 µm apart from the jet orifice. **C** Kymograph of mean radial cell displacements u_r (µm) over radial distance r (µm), for HMEC-1 in monolayer treated with 5 µM FAK-14. Kymograph corresponds to representative recording shown in panel B. The flow exposure period is indicated between black dashed lines. **D** Integral of radial cell displacements u_r (y-axis, µm) over time (x-axis, h) for HMEC-1 cells treated with 5 µM FAK-14 (blue) during a 30 h TFM recording. Cells were under static conditions from 0-5 h, exposed to flow from 5-25 h, and flow was ceased from 25-30 h. Positive (negative) values indicate movement away from (towards) the center of the jet. Time of flow exposure is shown in red shade. Mean±SEM. For panels B-D, data originate from n=3 independent recordings originating from one independent experiment.

SUPPLEMENTARY FIGURE 8

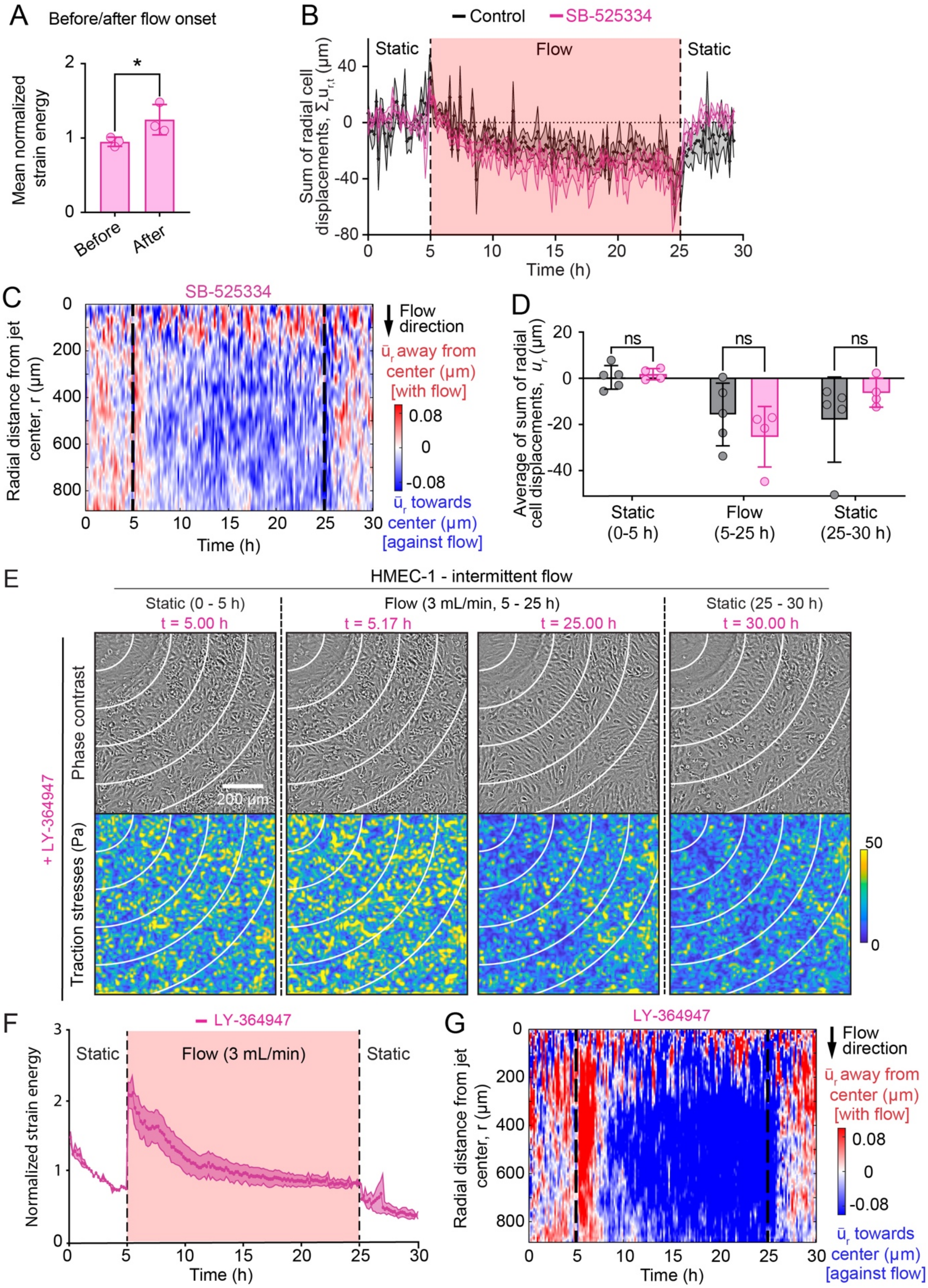

Supplementary Fig. 8: µEC treated with SB-525334 or LY-364947 do not increase their traction forces in response to flow.

A Bar plot of average normalized strain energy (y-axis, µNµm) imparted by HMEC-1 in monolayer treated with 10 µM SB-525334 (magenta) before and after flow exposure. Left bar plot refers to normalized strain energy values averaged over 1 h prior to flow exposure, while right bar plot refers to values averaged over 1 h immediately after flow exposure. Superimposed circles refer to average value of N=3 independent TFM recordings. Mean±SD. WRST, $p<0.05$: *. **B** Radial displacements u_r (y-axis, µm) integrated over radial distance as a function of time (x-axis, h) for HMEC-1 cells treated with 10 µM SB-525334 (magenta) or vehicle control (black) during a 30 h-long microscopy recording. Cells were under static conditions from 0–5 h, exposed to flow from 5–25 h, and then returned to static conditions from 25–30 h. Positive values indicate movement away from, and negative values movement toward, the center of the jet. Flow period is indicated by the red shading. Mean ± SEM, N = 4 independent experiments. **C** Representative kymograph of mean radial displacement u_r (in µm, see colorbar) as a function of radial distance r (y-axis) and time (x-axis, h) for HMEC-1 cells treated with 10 µM SB-525334. The polar coordinate system was centered at the field of view center (jet orifice). Flow occurred between 5–25 h, as marked by dashed vertical lines. **D** Bar plot showing the mean integral (i.e., sum) of radial cell displacements (u_r) over the indicated time intervals for HMEC-1 monolayers treated with vehicle control (black) or 10 µM SB-525334 (magenta). Individual recording means are overlaid as circles. Mean±SD, N=5 independent recordings. KWTD, $p>0.05$: non-significant, ns. **E** Representative images of HMEC-1 in monolayer treated with 10 µM LY-364947 at various stages: before flow exposure (1st column), immediately after flow initiation (2nd), 20 h into flow (3rd), and 5 h post-flow cessation (4th). Rows show phase contrast (1st) and cellular traction stresses (2nd, Pa). Cells on a 3 kPa matrix were exposed to flow using the impinging flow device. Jet center positioned at the center of the field of view imaged, but only a quarter of the field of view is shown. White or black contours mark concentric regions spaced 185 µm apart from the jet orifice. **F** Normalized strain energy (i.e., mechanical work imparted by HMEC-1) treated with 10 µM LY-364947 over time. Red line (intermittent flow-exposed) refers to cells that were initially under static conditions (0–5 h), exposed to flow (5–25 h, red shading), and then under static conditions again (25–35 h). Black line refers to control cells never exposed to flow. Mean±SEM, n=3 recordings originating from independent wells were analyzed originating from N=1 independent experiment. Strain energy was normalized to its value at 2.5 h after the start of the recording, corresponding to the midpoint

of the 5-h static phase preceding flow exposure. **G** Kymograph of mean radial cell displacements u_r (µm) over radial distance r (µm), for HMEC-1 in monolayer treated with 10 µM LY-364947. Kymograph corresponds to representative recording shown in panel B. The flow exposure period is indicated between black dashed lines. The flow exposure period is indicated between black dashed lines.

SUPPLEMENTARY VIDEO LEGENDS

Supplementary Video 1: Advection of tracer beads suspended in culture medium within the impinging flow jet device and traction force microscopy (TFM) recording on a cell-free hydrogel exposed to flow.

The first part of the movie shows the movement of 2.0 µm diameter carboxylated latex tracer beads suspended in HMEC-1 culture medium and circulating through the impinging flow jet device in the absence of cells. The flow rate is 3 mL/min. Time and scale bars are shown. The movement of the tracer beads from the jet orifice (center of field of view) outwards, due to advection is evident.

The second part of the movie shows a TFM recording acquired from a cell-free 3 kPa polyacrylamide hydrogel with the impinging flow jet device mounted on top, while the flow rate was sequentially changed from 0 mL/min (static) to 3 mL/min, then to 4.5 mL/min, and finally returned to 0 mL/min (static). From left to right, the panels display: (1) fluorescence images of tracer beads embedded within the hydrogel; (2) calculated ECM deformations (µm) using the same color scale (c-axis) as that used for TFM experiments on cells; (3) ECM deformations (µm) displayed using a reduced color scale to visualize small displacements; and (4) the temporal evolution of the mean ECM deformation (µm) over time (min). The shaded regions indicate the applied flow conditions: white, 0 mL/min; pink, 3 mL/min; and red, 4.5 mL/min. Time and scale bars are shown on the movie.

Supplementary Video 2: Time-lapse movie showing reduced cell motility for µEC in monolayer under flow as compared to static conditions.

Left column shows phase contrast images of µEC under static condition (top) or exposed to flow (bottom) for 20 h using the impinging flow device set at 3 mL/min. Middle column shows cell displacement (µm). Right column shows cell displacement vectors (black arrows) scaled 15x.

Note the frame rate is 10 min. The first part of the movie refers to HMEC-1, followed by hCMEC/D3. One quarter of the field of view is shown, with white (or black) contours marking concentric regions spaced 185 μm from the jet orifice. Scale bar (μm) and time post-flow initiation (h) are indicated on the movie.

Supplementary Video 3: μEC exhibit an acute and sustained decrease in migration speed in response to flow, with partial recovery of their speed after flow cessation.
Left column shows phase contrast images of μEC under static condition (top) or exposed to flow (bottom) for 20 h using the impinging flow device set at 3 mL/min. Cells exposed to intermittent flow were under static conditions from 0-5 h, exposed to flow from 5-25 h, and then monitored post-flow cessation from 25-35 h. Middle column shows cell displacement (μm), and the inset plot shows mean cell speed (μm/h) at each instance of time (h). Right column shows cell displacement vectors (black arrows) scaled 20x, and the inset plot shows mean correlation length of motion (μm) at each instance of time. In the inset plots, the period of flow exposure is indicated by red shading. Frame rate is 10 min. The first part of the movie refers to HMEC-1, followed by hCMEC/D3. One quarter of the field of view is shown, with white (or black) contours marking concentric regions spaced 185 μm from the jet orifice. Scale bar (μm) and time post-flow initiation (h) are indicated on the movie.

Supplementary Video 4: μEC increase their traction stresses during flow exposure.
Traction force microscopy on HMEC-1 cells under static conditions (top) or exposed to flow using the impinging flow device (bottom). Cells exposed to intermittent flow were under static conditions from 0-5 h, exposed to flow from 5-25 h, and then monitored post-flow cessation from 25-35 h. Columns show: phase contrast image; ECM deformations imparted by the cells (color indicates magnitude in μm); traction stresses exerted by cells (color indicates magnitude in Pa); and tensile monolayer stresses, σ_l (Pa). One quarter of the field of view is shown, with white contours marking concentric regions spaced 185 μm from the jet orifice. Frame rate is 10 min. The first part of the movie refers to HMEC-1, followed by hCMEC/D3 in the second part. Inset plot in column 3 shows the evolution of cells' strain energy (mechanical work, μNμm) as a function of time (h). Inset plot in column 4 shows the evolution of cells' mean tensile monolayer stresses (Pa) as a function of time (h). The period of flow exposure is indicated by red shading in the inset plots. Scale bar (μm) and time (h) are indicated on the movie.

Supplementary Video 5: FAK inhibition blocks reverse endothelial rheotaxis, while ROCK inhibition attenuates it.

Representative traction force microscopy recording of HMEC-1 exposed to intermittent flow using the impinging flow device and treated with vehicle control (top), 10 μM Y-27632 (middle, ROCK inhibitor), or 5 μM PF-573228 (bottom, FAK inhibitor). Cells were under static conditions from 0-5 h, exposed to flow from 5-25 h, and then monitored post-flow cessation from 25-30 h. Columns show: phase contrast image; cell displacement vectors (blue arrows, scaled 30x); traction stresses exerted by cells (color indicates magnitude in Pa). One quarter of the field of view is shown, with white (or black) contours marking concentric regions spaced 185 μm from the jet orifice. Inset plot in column 2 shows the evolution of cells' migration speed (μm/h) as a function of time (h). Inset plot in column 3 shows the evolution of cells' strain energy (mechanical work, μNμm) as a function of time (h). The period of flow exposure is indicated by red shading in the inset plots. Scale bar (μm) and time (h) are indicated on the movie.

Supplementary Video 6: Treatment with recombinant TGF-β1 leads to a gradual increase in HMEC-1 traction stresses.

Representative traction force microscopy recording of HMEC-1 under static conditions treated with vehicle control (top) or 10 ng/mL recombinant TGF-β1 (bottom). Note that TGF-β1 was added 2.5 h post-initiation of the microscopy recording. Columns show: phase contrast image; ECM deformations imparted by the cells (color indicates magnitude in μm); traction stresses exerted by cells (color indicates magnitude in Pa). Inset plot in column 3 shows the evolution of cells' strain energy (mechanical work, μNμm) as a function of time (h). Scale bar (μm) and time (h) are indicated on the movie.
